# Design of A3B-Porphyrin Conjugates with Terpyridine as Potential Theranostic Agents: Synthesis, Complexation with Fe(III), Gd(III), and Photodynamic Activity

**DOI:** 10.3390/pharmaceutics15010269

**Published:** 2023-01-12

**Authors:** Kseniya A. Zhdanova, Anastasia V. Ivantsova, Fedor Yu. Vyalba, Maxim N. Usachev, Margarita A. Gradova, Oleg V. Gradov, Natalia Yu. Karpechenko, Natal’ya A. Bragina

**Affiliations:** 1 Institute of Fine Chemical Technologies, MIREA—Russian Technological University, 119571 Moscow, Russia; 2 N.N. Semenov Federal Research Center for Chemical Physics, Russian Academy of Sciences, Kosygin Street 4, 119991 Moscow, Russia; 3 N.N. Blokhin National Medical Research Center of Oncology, Ministry of Health of Russia, 115522 Moscow, Russia; 4 Ministry of Health of Russia, Pirogov National Research Medical University, 117997 Moscow, Russia

**Keywords:** theranostics, PDT, diagnostics, porphyrins, terpyridine, conjugates, porphyrin metal complexes, Pluronic F127, polymer micelles

## Abstract

This paper reports on the design and synthesis of new multifunctional porphyrin-based therapeutic agents for potential therapeutic and diagnostic applications. Zinc complexes of A3B-type *meso*-arylporphyrins containing OH- and COOH- groups were modified with chelating ligands based on 4′-(4-methylphenyl)-2,2′:6′,2″-terpyridine derivatives in high yields. Novel complexes with Gd(III), Fe(III) were obtained for these conjugates. Aggregation behaviour in solutions of different solubilisers was studied to inform the selection of the optimal solubilising platform for the porphyrins obtained; their photophysical and photochemical properties were also characterised. Micellar Pluronic F127 formulation was found to be the most effective solubiliser for stabilising the fluorescence-active monomolecular form of the photosensitisers (PS). In vitro cytotoxicity of the compounds was studied on the HEP-2 cell line with and without irradiation for 1.5 and 24 h. As a result, the IC50 of compounds **12** and **14** at an irradiation dose of 8.073 J/cm^2^ was shown to be 1.87 ± 0.333 and 1.4 ± 0.152 μM, respectively; without irradiation, the compound had no toxic effect within the studied concentration range (1.5 h). A test for the inhibition of metabolic cooperation or promoter activity was also performed for the abovementioned compounds, showing the efficacy and safety of the conjugates obtained. Preliminary data have indicated the high potential of the new type of PS to be promising molecular theranostic agents.

## 1. Introduction

Combining diagnostic and therapeutic approaches to overcome oncological diseases currently seems to be a promising strategy for cancer treatment [1,2,3]. The integration of two methods, such as photodynamic therapy (PDT) and magnetic resonance imaging (MRI), can provide an effective cancer treatment [4,5,6,7]. PDT has several important advantages, namely low invasiveness, low systemic toxicity, locally targeted irradiation, and the possibility of repeated use with low cumulative toxicity and drug resistance. This approach provides good efficacy in cancer cell ablation and, importantly, significantly fewer side effects than traditional methods. MRI is a non-invasive technique and can provide detailed information in vivo on tissue anatomy, function, and metabolism, with excellent anatomical (high spatial) resolution [8]. The relevance of this method is its low invasiveness in addition to its high sensitivity and efficiency. Currently, non-specific contrast agents (CA) based on paramagnetic metals (Gd(III), Mn(II), Fe(III)) are used to improve sensitivity in clinical diagnostics in approximately 25–30% of all MRI scans [9,10]. The use of such CA is associated with increased nephrotoxicity risk due to the metal ion release from the complex; therefore, stable chelation is one of the most important tasks in the design of contrast agents. 

Natural porphyrins, such as chlorophyll and blood haem, and their numerous derivatives, have been extensively studied in various fields of medicine and engineering [11,12,13]. Synthetic porphyrins are characterised by the simplicity of synthesis, ease of chemical modification, and chemical stability. The wide variety of porphyrin derivatives and their modification pathways makes them promising compounds, with potential multimodal applications. Porphyrins exhibit good metal chelating properties, which enables their use in photodiagnostics [14,15]. It was previously shown that metalloporphyrins have a minimal background signal from cellular autofluorescence [16]. However, despite the widely known coordination abilities of porphyrins, there are limitations to obtaining stable complexes with large-radius paramagnetic metals, such as Gd(III) ions [15]. The formation of stable complexes with large atomic radius metals is limited for porphyrins and their hydrogenated derivatives, such as chlorins and bacteriochlorins [17]. This problem can be resolved by the introduction of an external chelating fragment into the macrocycle [18,19]. 

Substituted terpyridines (Tpy) are excellent ligands in coordination chemistry [20]. Terpyridine forms coordination chelates of the tridentate pincer (claw-shaped) type, due to the presence of three conjugated nitrogen atoms in the molecule [21,22]. Tpy can provide stable chelation with various metal cations, including large-radius metal ions (e.g., Gd(III)), in an almost flat geometry [23,24]. The combination of two heterocycles, namely, porphyrins and terpyridines, makes them suitable for various photobiological applications, because of the valuable photophysical and coordination properties of the resulting conjugates. Such conjugation can provide additional benefits for the PS amphiphilicity and improve proton relaxation, to increase MRI efficiency.

To date, several studies on the synthesis of conjugates based on tetrapyrroles and terpyridine derivatives and their external complexation are known. For example, a radioactive pharmaceutical agent for SPECT was developed [25]. Authors showed coordination of Re(I) by terpyridine moiety in porphyrin, which was regarded as the tpy-99mTc(I) complex. The work of the authors was dedicated to the synthesis of dyads and triads based on Tpy and porphyrin molecules coordinated with Rh(II) and Ru(II) metals and the study of the electron transfer in such systems [26]. Photoinduced charge separation over nanometer distances in a triad containing a zinc porphyrin (electron donor) and a gold(III) porphyrin (electron acceptor) appended to an Ir(III) bisterpyridine complex was shown [27].

This paper reports on the development of a rational design of A3B-type porphyrins bearing terpyridine moiety as potential multifunctional agents for imaging (MRI) and therapy (PDT). A suggestion is made, here, for a synthetic strategy for such conjugates, their complexation with Gd(III) and Fe(III) ions and preliminary in vitro experiments on Hep-2 cells. The strategy of creating bimodal agents within a single molecular object can increase the hydrophilicity of the PS in biological media. In addition, the presence of a lipophilic core in the molecule will increase the selectivity of absorption and accumulation of the theranostic agent in tumour tissues [28].

## 2. Materials and Methods

The used reagents were received commercially, solvents were purified according to standard procedures. Pyrrole was distilled over CaH_2_. Column chromatography was carried out on silica gel G60 (Macherey-Nagel, 230–400 mesh). Thin-layer chromatography was performed using aluminium sheets coated with silica gel 60 F254 (Macherey-Nagel, Düren, Germany). Compounds **7** and **11** were synthesised according [29].

NMR (^1^H and ^13^C) spectra of the synthesized compounds were recorded in CDCl_3_ using Bruker MSL-300 pulse Fourier transform spectrometer with TMS as an external standard. IR spectra (4000–500 cm^−1^) were received on Infralum FT 02 Fourier spectrometer (Lumex) with a resolution of 1 cm^−1^ (KBr tablets). DLS particle distribution was received using Zetasizer Nano ZS (Malvern). EAS spectra were recorded using Shimadzu UV-1800 spectrophotometer at room temperature in quartz cells with an optical path length of 10 mm. Steady-state fluorescence spectra were recorded using Cary Eclipse (Agilent) luminescence spectrometer in 1 cm^−1^ quartz cells with the excitation 415 nm (Soret band region) at room temperature.

MALDI mass spectra were registered on a Bruker autoflex speed time-of-flight (TOF) mass spectrometer (Bruker Daltonics Inc., Bremen, Germany) equipped with a solid-state UV laser with λ = 355 nm (frequency of 1 kHz, 1000 pulses for each sample) and a reflectron in the mode of positively charged ions registration. HPLC and HRMS specrta were conducted on a Vanquish ultrahigh performance liquid chromatography system coupled with a Q-Exactive HF-X hybrid high-resolution mass spectrometer with electrospray ionization. Chromatographic separation was carried out on a Hypersil Gold C8 reversedphase column 50 cm long with internal diameter of 2.1 mm, sorbent particles of 1.9 μm in diameter (Thermo Scientific, Germany, catalog no. 10238700). Milli Q Deionized water with specific resistance of 18.2 Ohm/cm was used as a component A of mobile phase. Isopropyl alcohol for HLPC (Scharlau, Sentmenat, Spain, catalog no. 603-117-00-0) was used as a component B of mobile phase. Volume speed of mobile phase flow is 0.25 mL/min. Column temperature is 40 °C. Volume of aliquot applied on column is 3 μL. Gradient elution mode. Gradient of mobile phase composition: 0.0–1.0 min, component B, 5%; 1.0–12.0 min, linear increase in component B up to 95%; 12.0–14.0 min, component B, 95%; 14.0–14.1 min, stepwise decrease of component B down to 5%; 14.1–15.0 min, component B, 5%. Ionization conditions: voltage on spraying capillary of ±4.0 kV (depending on polarity); spraying gas flow rate of 35 arb. units; auxiliary gas flow rate of 15 arb. units; drying gas flow rate of 5 arb. units; temperature of spraying capillary of 200 °C; temperature of input capillary of mass spectrometer is 350 °C; temperature of auxiliary gas is 200 °C; voltage on input lens of ion optics is 50 V. 

*Aggregation behavior, photophysical characterization and solubilisation studies*. Photophysical properties of zinc complexes of amphiphilic *meso*-arylporphyrins and their conjugates with terpyridine were studied in DMF and aqueous solutions of different solubilisers. Stock solutions of 1 mM of the studied compounds in DMF were added upon stirring to a micellar aqueous solution of a solubiliser (3 mL). The final concentration of the porphyrins was 3.5 µM. UV-vis absorption spectra were recorded using HACH DR-4000V spectrophotometer (Hach-Lange, Loveland, CO, USA) within the wavelength range of 320–800 nm with a step of 1 nm in quartz cells (10 mm) at room temperature. Steady-state fluorescence spectra were recorded using Perkin Elmer LS-50 (USA) luminescent spectrometer under similar conditions with the Soret band maximum excitation. Fluorescence quantum yields (Φ*_F_*) for Zn(II) complexes in DMF were measured according to the procedure [30,31] using ZnTPP as a standard (Φ*_F_* = 0.033).

*Photochemical experiments for singlet oxygen quantum yield determination*. Porphyrin-containing samples were irradiated in quartz cells at room temperature in air-saturated DMF solutions. Light source consisted of halogen lamp (150 W), thermal and UV filters, a three-lens spherical condenser with a reflector, and a cut-off filter (λ ≥ 500 nm). The light flux power was 10 mW/cm^2^. The efficiency of photosensitised ^1^O_2_ generation in porphyrin solutions was estimated from the dynamics of the absorbance decrease at 415 nm, corresponding to the absorption maximum of the selective ^1^O_2_ acceptor-1,3-diphenylisobenzofuran (DPBF). DPBF was added to the solution of the tested compound in the dark immediately before the start of the photochemical experiment (C_DPBF_ = 0.1 mM). Porphyrin concentration was maintained at about 2 μM in the all photochemical experiments so that the sample absorbance in the Q-band region did not exceed 0.05 in order to minimize the light filter effect. The singlet oxygen quantum yield (Φ_Δ_) were calculated according to the known procedure [32,33,34] with ZnTPP being a standard (Φ_Δ_ = 0.74 in DMF).

*Biological activity*. Cell viability study (MTT-test) on Hep2 (human laryngeal cancer) and NKE (normal kidney epithelial) cell lines. For this study, cells were plated into 96-well plates (BD Micro-FinePlus, Franklin Lakes, USA) (6 × 10^3^ cells in 180 μL of culture medium) and incubated for 24 h at 37 °C in 5% CO_2_. The medium was standard DMEM (PanEco, Moscow, Russia) with penicillin-streptomycin (50 units/mL) (PanEco, Moscow, Russia) supplemented with 10% fetal bovine serum (PanEco, Russia). After this time, culture medium was replaced with a similar volume of Hanks’ solution (PanEco, Russia) with glucose added at a final concentration of 4.5 mg/mL. On the day of the experiment, serial dilutions of the compound in deionised water were made and 20 μL was added to the cell culture. The cell culture thus obtained was irradiated for 90 min using a Medical Therapy Philips TL 20W/52 lamp (wavelength 400–500 nm), power 2.3 mW. In parallel, the cell culture was incubated with the compound in the dark. After 90 min, in both cases, Hanks’ solution with the compound was replaced with DMEM medium without serum. The cell culture was then left for 24 h at 37 °C in 5% CO_2_ in the dark, after which 10 μL of MTT reagent solution (5 mg/mL, PanEco, Russia) was added and incubated for another 3.5 h. The formed formazan in cells was dissolved in 100 μL DMSO. The optical density of the solution was measured on a MultiScan MCC 340 spectrophotometer (Labsystems, Kennett Square, PA, USA) at 540 nm. Each series of experiments was repeated 3 times. The concentration of the compound giving 50% of the maximum toxic effect (IC_50_) was calculated from the titration curves. Statistical processing of the obtained data was performed using Excel program package (Microsoft).

*Gap junction-mediated intercellular communication test*. The IAR2 cell line (rat liver epithelium) was used for this study. Cells were plated in 24-well plates at 1 × 105 per well and incubated in DMEM medium (PanEco, Russia) with penicillin-streptomycin (50 units/mL) (PanEco, Russia) and 10% fetal bovine serum (PanEco, Russia) at 37 °C in 5% CO_2_. When the cell monolayer was reached, the medium was changed to a similar one but without serum to avoid interaction of the substance with it. Compound **12** was added until the final concentration reached 4.5 μM; 2.25 μM; 1.125 μM and 0.56 μM. Cells serving as positive controls were treated with TPA (12-O-tetradecanoylphorbol-13-acetate (TPA), Invitrogene, Waltham, MA, USA) at a final concentration of 5 μg/mL (dissociates intercellular contacts). Cell culture with the substances was left for 4 h at 37 °C in 5% CO_2_. At the end of incubation, cells were washed with PBS and coated with luminescent indicator solution (Lucifer yellow, Invitrogene, USA), and then two parallel and two perpendicular scratches were applied to the cell monolayer using a surgical scalpel. The cells were incubated for 4 min at 37 °C in a humid atmosphere without access to light, washed three times with PBS, and coated with fixative solution. After incubation for 24 h, the cell monolayer was analyzed using a Zeiss fluorescence microscope with a phototip (Axioplan 2 imaging) filter for FITC fluorescence at 400× magnification. For each dose of the compound under study, 18–22 independent images were taken of the cell monolayer area adjacent to the scratch line. For each image, the order of cells carrying the fluorescent dye was determined. The separation of intercellular contacts was determined as the ratio of the average number of cell layers into which dye penetration occurred for each concentration of the compound under study to the average number of cell layers into which dye penetration occurred in control samples (cells cultured without the compound). Statistics were calculated using Student’s *t*-test using the Microsoft Excel software package.

*Comet assay*. NKE cells (human kidney epithelium) were seeded into 24-well plates at 8 × 10^4^ per well and incubated in DMEM medium (PanEco, Russia) with penicillin-streptomycin (50 units/mL) (PanEco, Russia) and 10% fetal bovine serum (PanEco, Russia) at 37 °C in 5% CO_2_. After 24 h, the medium was changed to a similar one but without serum to avoid interaction of the studied substance with it. Compound **14** was added until the final concentration reached 4.5 μM; 1.5 μM; 0.5 μM and 0.17 μM. Cells serving as positive controls were treated with ifosfamide (Baxter Oncology, Germany) at a final concentration of 1 μg/mL. Cell culture with the substances was left for 24 h at 37 °C in 5% CO_2_. Once this time was reached, the cells were removed from the substrate and washed twice with phosphate-salt buffer (PBS). Direct Comet assay was performed using OxiSelect™ Comet Assay Kit (Cell Biolabs, USA) according to the manufacturer’s protocol. Samples were stained with fluorescent dye Sybr Gold, Invitrogene, USA, and examined with a Zeiss microscope using a filter for FITC-fluorescence. All necessary measurements were performed using the computer program CometScore2.0. Statistics were calculated using Student’s *t*-test using the Microsoft Excel software package.

*Synthesis. Synthesis of 4′-(4-methylphenyl)-2,2′:6′,2″-terpyridine* **1**. A mixture of 2-acetylpyridine (4.84 g, 40 mmol), 4-methylbenzaldehyde (2.40 g, 20 mmol) was dissolved in ethanol (100 mL). Then the solution of granulated 85% KOH (3.08 g, 40 mmol) in 30% ammonia aqueous solution (60 mL) was added to the reaction mixture. The solution was cooled to 22 °C, filtered and the beige solid was washed with cold EtOH. The purification was carried out by recrystallisation of ethanol. The resulting white crystals were dried in vacuo over P_2_O_5_. The yield was 4.76 g (75%). ^1^H NMR (300 MHz CDCl_3_, δ, ppm:) 8.54–8.69 (4H, m, 3′5′, 6,6″), 8.59 (2H, d, *J* = 7.02 Hz, 3,3″), 7.81 (4H, m, 5,5″, 2,6ArH), 7.47 (2H, d, *J* = 8.26 Hz, 3,5ArH), 7.28 (2H, dd, *J*_1_ = 1.07 Hz, *J*_2_ = 4.79 Hz, 4,4″), 2.6 (3H, s,). ^13^C NMR (CDCl_3_, δ, ppm): 156.65, 154.57, 150.19, 147.25, 139.76, 138.78, 136.28, 129.84, 127.62, 124.85, 124.09, 123.99, 61.57, 21.12, 13.79. MALDI-TOF MS, *m*/*z*: 323.919 [M^+^], the molecular formula of C22H17N3 requires 323.39.

*Synthesis of 4′-(4-bromomethylphenyl)-2,2′:6′,2″-terpyridine **2***. A mixture of 4′-(4-methylphenyl)-2,2′:6′,2″-terpyridine **1** (0.5 g, 1.55 mmol) and N-bromosuccinimide (NBS) (0.33 g, 1.85 mmol) was dissolved in dry CCl_4_ (10 mL) and then 20 mg (0.12 mmol) of α,α′-azobis(isobutyroniltrile) (AIBN) was added. The reaction was boiled under stirring for 4 h. The hot reaction mixture was filtered off and evaporated at the rotary evaporator. The target product was purified by recrystallization in ethanol and dried in vacuo over P_2_O_5_. The yield was 0.51 g (80%). ^1^H NMR (300 MHz CDCl_3_, δ, ppm:) 4.49 (2H, s, CH_2_), 7.26 (2H, dd, *J*_1_ = 1.07 Hz, *J*_2_ = 4.79 Hz, 4,4″), 7.44 (2H, d, *J* = 8.26 Hz, 3,5ArH), 7.78 (4H, m, 5,5″, 2,6ArH), 8.60 (2H, d, *J* = 7.02 Hz, 3,3″), 8.65 (4H, m, 3′5′, 6,6″). ^13^C NMR (CDCl_3_, δ, ppm:) 155.70, 149.392, 149.02, 138.56, 138.4, 136.9, 129.6, 127.7, 124.33, 121.71, 118.7, 32.9. MALDI-TOF MS, *m*/*z*: 402.006 [M^+^], the molecular formula of C22H16N3Br requires 402.286.

*Synthesis of 4′-(4-amidomethylphenyl)-2,2′:6′,2″-terpyridine* **3**. First 4′-(4-Azidomethylphenyl)-2,2′:6′,2″-terpyridine was prepared. A mixture of compound **2** (0.130 g, 0.32 mmol) and sodium azide (0.031 g, 0.50 mmol) in 7 mL DMF was stirred at 70 °C in an argon flow for 20 h without light. The reaction mixture was extracted in dichloromethane/water (*v*/*v* 1:1). The organic layer was dried over Na_2_SO_4_ and evaporated *in vacuo*. The formed solid was recrystallized in methanol and dried *in vacuo* over P_2_O_5_. The yield was 100 mg (85%). ^1^H NMR (300 MHz, CDCl_3_ δ, ppm): 8.76 (s, 2H), 8.74 (d, *J* = 6.3 Hz, 2H), 8.69 (d, *J* = 7.8 Hz, 2H), 7.94 (d, *J* = 8.1 Hz, 2H), 7.89 (m, 2H), 7.48 (d, *J* = 8.1 Hz, 2H), 7.37(m, 2H), 4.47 (s, 2H). ^13^C NMR (300 MHz, CDCl_3_, δ, ppm:) 156.1, 155.9, 149.6, 149.1, 138.5, 136.9, 136.2, 128.7, 127.8, 123.8, 121.3, 118.8, 54.4. IR spectra, cm^−1^: 3050, 2361, 2088 (st, N_3_), 1246 (δ, N_3_).

4′-(4-Azidomethylphenyl)-2,2′:6′,2″-terpyridine (0.30 g, 0.82 mmol) was dissolved in THF (3 mL) and stirred for 5 min at 0 °C. Then triphenylphosphine (0.324 g, 1.24 mmol) was added and the resulting mixture was stirred for 15 h at rt. Then, 1 mL of H_2_O was added, and the solution was stirred for 1 h. The purification was carried out by mashing the sediment first in diethyl ether, then in *n*-hexane, and recrystallized in acetone/ethanol system. The resulting beige crystals were dried *in vacuo* over P_2_O_5_. The yield was 0.21 g (76%). ^1^H NMR (CDCl_3_, δ, ppm): 4.00 (s, 2 H), 7.26 (dd, *J*_1_ = 1.07 Hz, *J*_2_ = 4.79 Hz, 2H), 7.58 (d, *J* = 8.26 Hz, 2 H), 8.09 (m, 4 H), 8.92 (d, *J* = 7.02 Hz, 2H), 8.98 (m, 4 H). ^13^C NMR (CDCl_3_, δ, ppm): 156.2, 155.9, 149.9, 149.1, 144.2, 136.8, 134.9, 127.6, 127.4, 123.7, 121.31, 118.64, 46.1. MALDI-TOF MS, *m*/*z*: 339.473 [M+], the molecular formula of C22H18N4 requires 338.405.

*General methodology A for the synthesis of A3B-type meso-arylporphyrins*

Benzaldehydes were dissolved in a mixture of organic solvents (acetic acid, nitrobenzene, and propionic acid (*v*/*v*/*v* 3:3:6)) and stirred under boiling for 10 min. Pyrrole dissolved in nitrobenzene was added dropwise and the boiled mixture was stirred for 2 h. The cooled reaction mixture was extracted in dichloromethane/water system (*v*/*v* 1:1), triethylamine was used for neutralization. The organic layer was dried with anhydrous sodium sulfate and evaporated in vacuo.

*Synthesis of 5-(4-hydroxyphenyl)-10,15,20-tris(4-n-hexyloxyphenyl)porphyrin* **4**. Compound **4** was prepared according to General methodology A from 1.00 g (4.85 mmol) of 4-*n*-hexyloxybenzaldehyde, 0.204 g (1.62 mmol) of 4-hydroxybenzaldehyde and 0.434 g of pyrrole (6.47 mmol). The target product was purified by column chromatography using G 60 silica gel and CH_2_Cl_2_ as an eluent. The resulting purple solid was dried *in vacuo* over P_2_O_5_. The yield is 0.19 g (12.6%), R_f_ = 0.35 (CH_2_Cl_2_). EAS (CH_2_Cl_2_) λmax, nm (lgε): 421.8 (4.98); 516.8 (3.01); 552.6 (2.84); 581.0 (2.56); 649.6 (2.51). ^1^H NMR (300 MHz, CDCl_3_, δ, ppm:) 9.01-8.92 (8H, m, H2, H3, H7, H8, H12, H13, H17, H18), 8.3 (6H, d, *J* = 8.44 Hz, 2,6ArH), 7.99 (2H, d, *J* = 8.25 Hz, 2,6ArH), 7.22 (6H, m, 7.42, 3,5ArH), 7.00 (2H, d, *J* = 8.8 Hz, 3,5ArH), 4.16–4.23 (m, -OCH_2_, 6H), 2.00–1.84 (6H, m, -OCH_2_CH_2_CH_2_), 1.69–1.71 (6H, m, -O(CH_2_)_2_CH_2_CH_2_), 1.50–1.27 (12H, m, CH_2_(CH_2_)_2_CH_3_), 0.91–0.88 (9H, m, CH_3_), −2.88 (2H, br. s, NH). ^13^C NMR (CDCl_3_, δ, ppm): 190.11, 167.2, 158.65, 350.08, 133.84, 131.74, 130.73, 128.17, 114.51, 113.30, 111.9, 67.70, 39.32, 31.51, 28.7, 25.3, 21.8, 14.52, 10.28. MALDI-TOF MS, *m*/*z*: 930.401 [M-H]^+^, the molecular formula of C62H66N4O4 requires 931.212.

*Synthesis of 5-(4-pyridyl)-10,15,20-tris(4-n-hexyloxyphenyl)porphyrin* **5**. Compound **5** was prepared according to General methodology A from 0.620 g (5.55 mmol) of 4-*n*-hexyloxybenzaldehyde, 0.62 g (1.85 mmol) of 4-pyridylcarbaldehyde 0.5 g of pyrrole (7.5 mmol). The targed porphyrin was purified by column chromatography on aluminum oxide. The eluent was CH_2_Cl_2_/EtOAc (*v*/*v* 100:1). The product was recrystallized in methanol. The resulting purple solid was dried *in vacuo* over P_2_O_5_. The yield 0.24 g (11%), R*_f_* = 0.35 (CH_2_Cl_2_/EtOAc 100:1). EAS (CHCl_3_) λmax, nm (lgε): 420.0 (5.7); 517.4 (4.50); 553.6 (4.07); 592.0 (3.8); 648.0 (3.78). ^1^H NMR (CDCl_3_, δ, ppm), 9.04 (2H, d, *J* = 4.41 Hz, 3,5-Py), 8.93 (6H, d, *J* = 4.8 Hz, H2, H8, H12, H13, H17, H18), 8.79 (2H, d*, J* = 4.82 Hz, H2, H3), 8.78 (2H, d, *J* = 4.9 Hz, H2, H8), 8.18 (2H, m, 2,6Py-H), 8.12 (2H, d, *J* = 8.67 Hz, 2,6Ar-H), 7.29 (6H, d, *J* = 10.48 Hz, 3,5ArH), 4.25 (6H, t, *J* = 6.5 Hz, -OCH_2_CH_2_), 1.99 (6H, m, -OCH_2_CH_2_CH_2_), 1.64 (6H, m, -O(CH_2_)_2_CH_2_CH_2_), 1.38 (12H, m, (CH_2_)_2_CH_3_), 0.96 (9H, m, CH_3_), −2.75 (s, 2H, NH-Pyrrole). ^13^C NMR (CDCl_3_, δ, ppm): 158.4, 148.9, 147.4, 135.1, 133.1, 131.7, 128.5, 120.0, 119.3, 118.94, 115.5, 112.0, 76.1, 31.6, 29.6, 26.2, 22.0, 13.5. MALDI-TOF-MS, *m*/*z*: 916.833 [M^+^]; the molecular formula of C61H65N5O3 requires 916.200.

*5-(4-Aminophenyl)-10,15,20-tris(4-hexyloxyphenyl)porphyrin* **6**. Compound **6** was prepared according to General Method A in two steps. First, 5-(4-acetamidophenyl)-10,15,20-tri(4-hexyloxyphenyl)porphyrin was received from 0.5 g (2.427 mmol) of 4-*n*-hexyloxybenzaldehyde, 0.132 g (0.809 mmol) of *p*-acetamidobenzaldehyde and 0.217 g (3.236 mmol) of pyrrole. The yield was 0.086 g (11%). EAS (CH_2_Cl_2_) λmax/nm, lgε: 418.4 (5.52); 518.4 (4.35); 556.0 (4.05); 593.2 (3.94); 649.4 (3.87). Rf 0.35 (CH_2_Cl_2_). ^1^H NMR spectrum (CDCl_3_, δ, ppm): 8.75 (8H, m, H2, H3, H7, H8, H12, H13, H17, H18), 7.95 (6H, m, 2,6ArH), 8.11 (2H, m, 2,6ArH), 7.51 (6H, m, 3,5ArH), 7.30 (2H, m, 3,5ArH), 3.99 (6H, m, -OCH_2_), 1.98 (3H, s, C(O)CH_3_), 1.75 (6H, m, -OCH_2_CH_2_CH_2_), 1.43 (2H, m, O(CH_2_)_2_CH_2_CH_2_), 1.28 (12H, m, CH_2_(CH_2_)_2_CH_3_), 0.85 (9H, m, -CH_3_), −2.88(2H, s, NH). ^13^C NMR (CDCl_3_, δ, ppm): 168.99, 158.51, 135.64, 134.64, 132.36, 131.43, 128.64, 119.76, 113.5, 38.75, 32.10, 30.47, 29.82, 28.73, 26.08, 22.57, 14.40. IR spectrum ν/cm^−1^: 3345 cm^−1^(st NH), 1600 cm^−1^ (δ NH), 1265, 1042 cm^−1^ (ArOCH_2_). MALDI-TOF-MS, *m*/*z*: 974.3 [M+1]^+^; the molecular formula of C64H69N5O4 requires 972.264.

5-(4-Acetamidophenyl)-10,15,20-tris(4-hexyloxyphenyl)porphyrin (0.1 mmol) was dissolved in 8 mL of trifluoroacetic acid, then 10 mL of concentrated hydrochloric acid was added and stirred for 24 h at 80 °C. Extraction was carried out in a CH_2_Cl_2_/H_2_O system, washed with 10% Na_2_CO_3_. The resulting solution was evaporated and purified by column chromatography on G 60 silica gel. The yield was 79 mg (85%). EAS (CH_2_Cl_2_) λmax/nm, lgε: 419.2 (5.57); 515.6 (4.30); 555.4 (4.15); 608.6 (3.99); 649.6 (3.88). Rf 0.75 (CH_2_Cl_2_). ^1^H NMR spectrum (CDCl_3_, δ, ppm): δ: 9.01 (8H, m, H2, H3, H7, H8, H12, H13, H17, H18), 8.13 (6H, m., 2,6Ar-H), 8.01 (2H, m., 2,6Ar-H), 7.29 (6H, м., 3,5Ar-H), 7.30 (2H, m., 3,5Ar-H), 4.28 (6H, m, -OCH_2_CH_2_), 4.26 (2H, s, NH_2_), 1.97 (6H, m., -OCH_2_CH_2_CH_2_), 1.64 (2H, м, O(CH_2_)_2_CH_2_CH_2_), 1.44 (12H, м., CH_2_(CH_2_)_2_CH_3_), 0.99 (9H, м., -CH_3_), −2.71 (br s, 2H, NH-Pyrrole). ^13^C NMR (CDCl_3_, δ, ppm): 158.79, 145.56, 135.58, 134.61, 132.49, 130.99, 120.79, 119.65, 113.47, 112.67, 67.60, 32.09, 29.70, 25.96, 22.52, 13.68. MALDI-TOF-MS, *m*/*z*: 931.3 [M+1]^+^; the molecular formula of C62H67N5O3 requires 930.227.

*General methodology B for the synthesis of zinc complexes of porphyrins*

A free-base porphyrin **4–7** was dissolved in CH_2_Cl_2_ (10 mL) and a tenfold excess of zinc acetate dihydrate in methanol (6 mL) was added. The reaction mixture was stirred for 4 h at room temperature and then was extracted in a CH_2_Cl_2_/H_2_O system (*v*/*v* 1:1). The organic layer was removed *in vacuo*, recrystallised in CH_2_Cl_2_/CH_3_OH system and dried over P_2_O_5_. The degree of the reaction was evaluated using an EAS.

*Synthesis of 5-(4-hydroxyphenyl)-10,15,20-tris(4-n-hexyloxyphenyl)porphynatozinc* **8**. Compound **8** was prepared according to the general methodology B from porphyrin **4** (0.190 g, 0.20 mmol) and zinc acetate dihydrate (0.45 g, 2.00 mmol). The yield was 0.185 g (98%). EAS (CH_2_Cl_2_) λmax, nm (lgε): 425.2 (5.58), 557.3 (4.14), 596 (3.86). ^1^H NMR (300 MHz, CDCl_3_, δ, ppm:) 8.90–8.85 (2H, m, H3, H7), 8.60-848 (6H, m, H2, H8, H12, H13, H17, H18), 8.21–7.99 (6H, m, 2,6Ar-H), 7.59–7.48 (2H, m, 2,6Ar-H), 7.32–7.26 (6H, m, 3,5Ar-H), 7.25 (2H, m, 3,5Ar-H), 4.29 (6H, d, *J* = 7.6 Hz, OCH_2_), 2.10–1.92 (6H, m, OCH_2_CH_2_), 1.74–1.59 (6H, m, O(CH_2_)_2_CH_2_), 1.52–1-40 (12H, m, (CH_2_)_2_CH_3_), 1.07–0.93 (9H, m, -CH_3_). ^13^C NMR (CDCl_3_, δ, ppm): 154.71, 146.87, 146.67, 143.52, 139.45, 137.51, 136.53, 130.31, 129.99, 129.84, 129.08, 126.52, 121.34, 121.17, 119.62, 116.64, 115.71, 68.81, 31.60, 29.64, 25.70, 22.66, 14.18. MALDI-TOF-MS, *m*/*z*: 993.425 [M+1]^+^, the molecular formula of C62H64N4O4Zn requires 994.586.

*Synthesis of 5-(4-pyridyl)-10,15,20-tri(4-n-hexyloxyphenyl)porphynatozinc* **9**. Compound **9** was prepared according to the general methodology B from porphyrin **5** (0.235 g, 0.26 mmol) and zinc acetate dihydrate(0.5 g, 2.60 mmol). The yield was 0.248 g (98%). EAS (CHCl_3_) λmax/nm (lgε): 424 (5.31); 550 (4.05); 592 (3.67). ^1^H NMR spectrum (CDCl_3_, δ, ppm): 9.06–8.99 (2H, m, 3,5-Py), 8.93 (2H, d, *J* = 4.8 Hz, 3H, 7H), 8.78 (6H, s, H2, H8, H12, H13, H17, H18), 8.20–8.15 (2H, m, 2,6Py), 8.14–8.08 (6H, m, 2,6-ArH), 7.31–7.26 (6H, m, Ph-H), 4.25 (6H, t, *J* = 6.5 Hz, -OCH_2_-), 1.99 (6H, m, OCH_2_CH_2_-), 1.64 (6H, m, -O(CH_2_)_2_CH_2_), 1.38 (12H, m, (CH_2_)_2_CH_3_), 0.98 (9H, m, CH_3_). ^13^C NMR (CDCl_3_, δ, ppm) 159.11, 147.99, 135.58, 134.07, 129.555, 120.92, 115.57, 112.53, 31.32, 29.36, 26.15, 22.27, 13.81. HRMS, *m*/*z*: 978.4278 [M-H]^+^, the molecular formula of C61H65N5O3Zn requires 978.4295.

*Synthesis of 5-(4-aminophenyl)-10,15,20-tris(4-hexyloxyphenyl)porphynatozinc* **10**. Compound **10** was prepared according to general methodology B from porphyrin **6** (0.235 g, 0.26 mmol) and zinc acetate dihydrate (0.5 g, 2.60 mmol). The yield was 0.248 g (98%). Rf 0.75 (CHCl_3_). EAS (CHCl_3_) λmax/nm (lgε): 430 (5.57); 556 (4.15); 595 (3.74). ^1^H NMR spectrum (CDCl_3_, δ, ppm):8.97 (2H, d, H3, H7), 8.91 (6H, m, H2, H8, H12, H13, H17, H18), 8.14 (6H, dd, 2-H), 8.014 (2H, d, 2-H), 7.25 (6H, m, 3-H), 7.00 (2H, d, 3-H), 4.22 (6H, t, OCH_2_), 1.94–2.03 (6H, m, -OCH_2_), 1.59–1.68 (6H, m, -OCH_2_CH_2_), 1.32-1.52 (18H, m, -O(CH_2_)_2_(CH_2_)_3_), 0.96 (9H, m, CH_3_). ^13^C NMR (CDCl_3_, δ, ppm): 158.2, 149.8, 147.3, 134.7, 133.2, 131.7, 128.6, 120.0, 119.5, 115.0, 111.9, 76.1, 31.1, 29.7, 25.3, 21.8, 13.3. HRMS, *m*/*z* 992.4442 [M]+; the molecular formula of C62H65N5O3Zn requires 992.4452.

*Synthesis of conjugate* **12**. A mixture containing 0.20 g (0.215 mmol) of compound **8**, 120 g (0.30 mmol) of compound **2**, and 0.120 g (2.15 mmol) of Cs_2_CO_3_ was boiled in 10 mL DMF in argon flow under stirring for 24 h. The cooled reaction mixture was extracted in CH_2_Cl_2_/H_2_O system (*v*/*v* 1:1). The organic layer was evaporated in vacuo. The target product was purified by column chromatography using aluminium oxide (neutral, activity 1) as a sorbent and CH_2_Cl_2_/EtOAc (*v*/*v* 50:1) as an eluent. The solid was recrystallized in the CH_2_Cl_2_/CH_3_CN system. The resulting solid was dried *in vacuo* over P_2_O_5_. The yield was 100 mg (40%). R_f_ Al_2_O_3_ = 0.5 (CH_2_Cl_2_/EtOAc (*v*/*v* 50:1)). EAS (CH_2_Cl_2_) λmax, nm (lgε): 426 (5.70), 555 (4.24), 596 (3.93). ^1^H NMR (300 MHz, CDCl_3_) δ, ppm: 8.97 (8H, H2, H3, H7, H8, H12, H13, H17, H18), 8.82 (2H, s, 3′5′), 8.75 (2H, m, 6, 6″), 8.71 (2H, m, 3, 3″), 8.15–8. 05 (8H, m, 2-H), 8.04 (2H, d, *J* = 8.2 Hz, Tpy 2,6ArH), 7.86 (2H, ddd, *J* = 7.7, 1.8 Hz, 5, 5″), 7.71 (2H, d, *J* = 7.5 Hz, Tpy 3,5ArH), 7.34–7.37 (2H, ddd, *J* = 7.56, 4.9, 1.2 Hz, 4, 4′), 7.32 (2H, d, *J* = 7.7 Hz, 3-H), 7.26–7.20 (6H, m,3-H), 5.27 (s, 2H, OCH_2_-Tpy), 4.21 (6H, t, *J*_1_ = *J*_2_= 6.43 Hz, OCH_2_), 2.05–1.85 (6H, m, OCH_2_CH_2_CH_2_), 1.71–1.55 (6H, m, O(CH_2_)_2_CH_2_CH_2_), 1.52–1.40 (12H, m, O(CH_2_)_3_(CH_2_)_2_CH_2_), 1.08–0.94 (9H, m, CH_3_). ^13^C NMR (CDCl_3_, δ, ppm): 158.96, 156.13, 149.98, 148.98, 148.80, 143.04, 137.87, 136.52, 135.57, 135.35, 134.02, 125.35, 123.72, 121.20, 119.87, 119.20, 112.70, 68.27, 31.06, 29.29, 25.59, 22.67, 14.09. HRMS MALDI-TOF, *m*/*z*: 1316.554 [M+H]^+^; the molecular formula of C84H79N7O4Zn requires 1315.549.

*Synthesis of conjugate **13***. A mixture containing 0.250 g (0.255 mmol) of compound **9**, 0.120 g (0.30 mmol) of compound **2**, and 0.120 g (2.15 mmol) of Cs_2_CO_3_ was boiled in 10 mL THF under argon flow while stirring for 36 h. The cooled reaction mixture was extracted in CH_2_Cl_2_/H_2_O system (*v*/*v* 1:1). The organic layer was evaporated in vacuo. The target product was purified by column chromatography using aluminium oxide as a sorbent (neutral, activity 1) and eluted with CH_2_Cl_2_/EtOAc (*v*/*v* 50:1). The solid was recrystallized in the dichloromethane/acetonitrile system. The resulting solid was dried *in vacuo* over P_2_O_5_. The yield was 100 mg (34%). R*_f_* Al_2_O_3_ = 0.4 (CH_2_Cl_2_/EtOAc (*v*/*v* 50:1)). EAS (CH_2_Cl_2_) λmax, nm (lgε): 426 (5.70), 555 (4.24), 596 (3.93). ^1^H NMR (300 MHz, CDCl_3_) δ, ppm: 9.38 (8H, br s, H2, H3, H7, H8, H12, H13, H17, H18), 9.22 (2H, d, *J* = 4.57 Hz, 3′5′), 9.18 (2H, d, *J* = 4.44 Hz, 6,6″), 8.78 (4H, s, 3,3″), 8.45 (8H, d, *J* = 7.87 Hz, 2-H), 8.37 (2H, d, *J =* 4.55 Hz, Tpy:α-Ph), 7.62 (2H, s, 5, 5″), 7.51 (8H, d, *J =* 8.00 Hz, 3-H), 7.25 (2H, m, 4, 4′), 5.08 (2H, N^+^CH_2_)_,_ 4.27 (6H, t, *J_1_ = J_2_* = 6.24 Hz, OCH_2_), 1.96 (6H, m, OCH_2_CH_2_), 1.63 (6H, m, O(CH_2_)_2_CH_2_), 1.39 (12H, m, O(CH_2_)_3_(CH_2_)_2_), 0.98 (9H, m, CH_3_). ^13^C NMR (CDCl_3_, δ, ppm): 158.96, 156.13, 149.98, 148.98, 148.80, 143.04, 137.87, 136.52, 135.57, 135.35, 134.02, 125.35, 123.72, 121.20, 119.87, 119.20, 112.70, 68.27, 31.06, 29.29, 25.59, 22.67, 14.09. MALDI-TOF-MS, *m*/*z*: 1301.821 [M-Br]^+^; the molecular formula of BrC83H79N8O3Zn requires 1381.880.

*Synthesis of conjugate* **14**. A solution of 4-([2,2′:6″,2″-terpyridin]-4′-yl)benzoic acid (80 mg, 0.21 mmol) in thionyl chloride (3 mL)was stirred at 80 °C in argon flow for 2 h. Then, thionyl chloride was removed in vacuo and the terpyridine acyl chloride was dried under high vacuum at 50 °C for 1 h. The received solid was dissolved in anhydrous THF (9 mL), and then compound **10** (70 mg, 0.07 mmol) and anhydrous triethylamine (0.1 mL) were added. The reaction mixture was heated under argon at 70 °C for 12 h. The resulting mixture was cooled, the solvent was removed in vacuo and extracted in CHCl_3_ (80 mL) and water (3 × 50 mL). The organic layer was dried under Na_2_SO_4_, filtered, and concentrated. The residue was purified by column chromatography using silica gel G60 (CH_2_Cl_2_:MeOH (50:3)) to obtain the target product as a purple solid (88 mg, 95%). EAS (CH_2_Cl_2_) λmax, nm (lgε): 430 (5.71), 565 (4.26), 598 (3.96). ^1^H NMR (300 MHz, CDCl_3_) δ, ppm: 8.90–8.85 (8H, m, H2, H3, H7, H8, H12, H13, H17, H18), 8.83 (2H, s, 3′5′-tpy), 8.79–8.75 (2H, m, 6,6″-tpy), 8.71 (2H, d, *J* = 8.0 Hz, 3,3″-tpy), 8.29–8.15 (6H, m, Ph-H, 3,5, 2,6-Ph-tpy), 8.14–8.04 (8H, m, 2,6ArH), 7.91 (2H, td, *J* = 7.8, 1.8 Hz, 4, 4″-tpy), 7.42–7.36 (2H, m, 5,5″-tpy), 7.31–7.22 (6H, m, 3,5ArH), 4.25 (6H, t, *J_1_ = J_2_* = 6.5 Hz, OCH_2_), 1.99 (6H, m, OCH_2_CH_2_CH_2_), 1.64 (6H, m, O(CH_2_)_2_CH_2_CH_2_), 1.51–1.40 (12H, m, O(CH_2_)_3_(CH_2_)_2_), 1.05–0.94 (9H, m, CH_3_). ^13^C NMR (CDCl_3_, δ, ppm): 158.96, 156.26, 150.20, 148.99, 142.79, 137.87, 136.54, 135.57, 134.87, 134.17, 125.42, 123.20, 121.55, 119.20, 112.42, 67.79, 31.56, 29.34, 25.89, 22.67, 14.09. HRMS (ESI, *m*/*z*): 1329.5469 [M]^+^; the molecular formula of C84H78N8O4Zn^2+^ [M+H]^+^ requires 1329.5427.

*Synthesis of conjugate* **15**. Porphyrin **11** (0.200 g, 0.19 mmol) and EDC (0.05 g, 0.32 mmol) were dissolved in THF (5 mL) and stirred in an argon flow at 0 °C for 10 min. DMAP (0.040 g, 0.32 mmol) and HOBT (0.045 g, 0.32 mmol) were added to the reaction mixture. After that, 0.08 g (0.32 mmol) of compound **3** was added dropwise in THF (5 mL) and reaction mixture was stirred in an argon flow for 8 h. The reaction mixture was extracted in THF/saturated NaCl solution (*v*/*v* 1:2) and neutralized with 0.5% hydrochloric acid solution. The organic layer was dried over Na_2_SO_4_, filtered, and concentrated. The crude product was purified by column chromatography using aluminium oxide and CH_2_Cl_2_/EtOAc (*v*/*v* 50:1) as an eluent. The solid was recrystallized in the THF/acetonitrile system. The resulting solid was dried in vacuo over P_2_O_5_. The yield was 0.190 g (75%). R_f_ Al_2_O_3_ = 0.55 (CH_2_Cl_2_/EtOAc 50:1). EAS (THF) λmax, nm (lgε): 426.5 (5.62), 558.2 (4.18), 598 (3.87). ^1^H NMR (300 MHz, CDCl_3_, δ, ppm.): 8.98 (4H, m, 3′5′, 6,6″), 8.93 (2H, d, *J =* 4.67 Hz, 3,3″), 8.73 (2H, d = 4.69 Hz, H3, H7), 8.53 (6H, m, H2, H8, H12, H13, H17, H18), 8.10 (2H, d, *J =* 8.60 Hz, ), 8.06 (6H, d, *J =* 8.53 Hz, 2-H), 7.99 (2H, d, *J =* 8.05 Hz, 2-H), 7.77 (2H, ddd, *J*_1_ *= J*_2_ *= J*_3_ = 1.56 Hz, 5,5″), 7.60 (2H, d, *J* = 8.14 Hz, 2,6-ArH) 7.22 (4H, m, 4, 4″, 3,5-ArH), 7.16 (6H, d, *J*_1_ = 8.60 Hz, 3-H), 6.91 (2H, d, *J*_1_ = 7.68 Hz, 3-H), 6.44 (1H, br s, NHCO), 4.15 (6H, m, OCH_2_), 1.84–1.96 (6H, m, OCH_2_CH_2_), 1.51–1.63 (6H, m, O(CH_2_)_2_CH_2_), 1.40–1.47 (12H, m, O(CH_2_)_3_(CH_2_)_2_), 0.95-1.05 (9H, m, CH_3_). ^13^C NMR (CDCl_3_, δ, ppm): 158.78, 156.11, 155.83, 150.80, 150.62, 150.55, 149.63, 149.61, 149.06, 136.83, 135.57, 135.26, 134.39, 132.30, 132.09, 131.45, 128.20, 127.46, 124.69, 123.79, 121.32, 121.03, 118.71, 112.66, 68.35, 31.85, 29.59, 29.56, 26.04, 26.01, 22.83, 22.81, 14.25. HRMS (ESI, *m*/*z*): 1343.5646 [M]^+^; the molecular formula of C85H80N8O4Zn requires 1343.5678.

*General methodology C for the synthesis of complexes with paramagnetic metals*

The conjugate was dissolved in 10 mL of CH_2_Cl_2_ or THF and a twofold excess of the corresponding salt in 6 mL of methanol was added and the reaction mixture was stirred at room temperature for 4–6 h. The solvents were removed in vacuo. The reaction mixture was extracted in a CH_2_Cl_2_/H_2_O system (*v*/*v* 1:1). The organic layer was dried under Na_2_SO_4_ and evaporated under vacuum, recrystallized in THF/CH_3_CN system and dried *in vacuo* over P_2_O_5_.

*General methodology D*

The conjugate was dissolved in 10 mL THF and 2.5 eq of the corresponding Gd (III) salt and 2.5 eq of anhydrous sodium acetate in 6 mL of methanol were added. The reaction mixture was stirred at 80 °C in an autoclave for 3 days. The work was carried out in a glove box in a nitrogen flow. After 48 h, a precipitate began to precipitate. The reaction mass was centrifuged and the precipitate was washed with 20 mL of acetonitrile followed by 20 mL of methanol. Afterwards the solid was recrystallized from THF/CH_3_CN. It was dried in vacuo over P_2_O_5_.

*Synthesis of complex **12a^1^***. Complex **12a^1^** was prepared according to the general methodology C from 0.085 g (0.064 mmol) of compound **12** dissolved in THF and 0.021 g (0.13 mmol) of anhydrous iron (III) chloride dissolved in CH_3_OH in N_2_ flow. The yield was 0.072 g (75%). R_f_ Al_2_O_3_ = 0.4 (CH_2_Cl_2_/CH_3_OH 50:1). EAS (THF) λmax, nm (lgε): 426 (5.51), 558 (4.02), 599 (3.99). HRMS, *m*/*z*: 1403.0753 [M-2Cl]^+^ the molecular formula of C84H79Cl3FeN7O4Zn requires 1478.1639.

*Synthesis of complex **12a^2^***. Complex **12a^2^** was prepared according to the general methodology C from 0.100 g (0.076 mmol) of compound **12** dissolved in THF, iron (III) acetylacetonate (0.054 g, 0.152 mmol), and sodium acetate (0.013 g, 0.152 mmol). The yield was 0.104 g (82%). R_f_ Al_2_O_3_ = 0.4 (CH_2_Cl_2_/CH_3_OH 50:1). EAS (THF) λmax, nm (lgε): 426 (5.69), 558 (4.10), 599 (3.74). MALDI-TOF-MS, *m*/*z*: 1605.635 [M-Zn]^+^; the molecular formula of C99H100FeN7O10Zn requires 1669.15. 

*Synthesis of complex **12b^1^***. Complex **12b^1^** was prepared according to the general methodology D from 0.080 g (0.06 mmol) of compound 13, 0.040 g (0.15 mmol), anhydrous GdCl_3_, 0.012 g (0.15 mmol) of anhydrous sodium acetate. The yield is 0.072 g (36%). Rf Al_2_O_3_ = 0.45 (CH_2_Cl_2_/CH_3_OH 90:1). EAS (CH_2_Cl_2_) λmax, nm (lgε): 432 (5.56), 568 (4.18), 598 (3.45). MALDI-TOF-MS, *m*/*z*: calculated 1579.5808 [M]^+^; the molecular formula of C84H79Cl3GdN7O4Zn requires 1579.5689.

*Synthesis of complex **13b^1^***. Complex **13b^1^** was prepared according to the general methodology C from 0.130 g (0.099 mmol) of compound **13** dissolved in CH_2_Cl_2_, 0.025 g (0.198 mmol) of anhydrous iron (III) chloride in a current of Ar. The yield is 0.039 g (25%). Rf Al_2_O_3_ = 0.45 (dichloromethane/methanol 80:1). EAS (CH_2_Cl_2_) λmax, nm (lgε): 429 (5.60), 560 (4.26), 596 (3.57). MALDI-TOF-MS, *m*/*z*: 1589.724 [M+CH_3_COO^−^]^+^; the molecular formula of CC83H79GdN8O3ZnCl3 requires 1565.5655.

*Synthesis of complex **14a^1^***. Preparation was carried out according to the general methodology C: from 0.080 g (0.06 mmol) of compound **14** dissolved in CH_2_Cl_2_ and 0.020 g (0.12 mmol) of anhydrous iron (III) chloride. The yield is 0.072 g (40%). R*_f_* Al_2_O_3_ = 0.60 (CH_2_Cl_2_/CH_3_OH 80:1). EAS (CH_2_Cl_2_) λmax, nm (lgε): 432 (5.56), 568 (4.18), 598 (3.45). MALDI-TOF-MS, *m*/*z*: 1512.087; the molecular formula of C84H78N8O4ZnCl3Fe requires 1491,1627.

*Synthesis of complex **14b^1^***. Preparation was carried out according to the general method D from 0.135 g (0.1 mmol) of compound **15**, 0.066 g (0.25 mmol) of anhydrous GdCl_3_, and 0.020 g (0.15 mmol) of anhydrous sodium acetate. The yield was 0.050 g (30%). EAS (CH_2_Cl_2_) λmax, nm (lgε): 426 (5.62), 556 (4.04), 598 (3.67). MALDI-TOF-MS, *m*/*z*: 1589.602 [M+2H]^+^; the molecular formula of C84H80Cl3GdN8O4Zn requires 1592.583.

*Synthesis of complex **15b^1^***. Preparation was carried out according to the general method D from 0.135 g (0.1 mmol) of compound **15**, 0.066 g (0.25 mmol) of anhydrous GdCl_3_, and 0.020 g (0.15 mmol) of anhydrous sodium acetate. The yield was 0.045 g (28%). Rf Al_2_O_3_ = 0.45 (CH_2_Cl_2_/CH_3_OH 90:1). EAS (CH_2_Cl_2_) λmax, nm (lgε): 426 (5.51), 556 (3.95), 598 (3.64). MALDI-TOF-MS, *m*/*z*: 1547.909 [M-Zn]^+^; the molecular formula of C85H80Cl3GdN8O4Zn requires 1606.594.

## 3. Results and Discussion

**Scheme 1:**
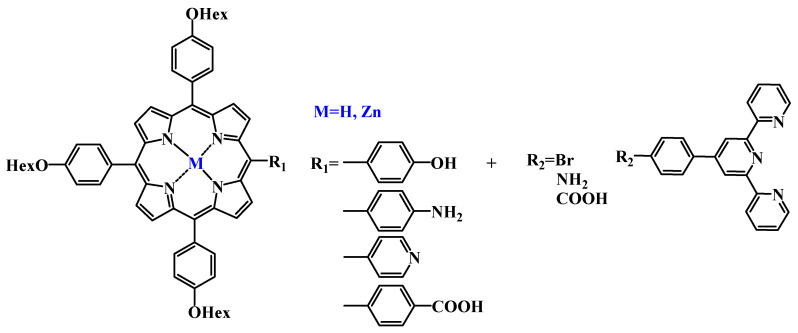
Design of the terpyridine-porphyrin conjugates.

*Synthesis. Meso-*arylporphyrins have long been known to be promising agents for PDT [35,36]. For most of them, however, problems associated with the hydrophobicity of these structures remain. In this work, the design of new multifunctional photodiagnostic and phototherapeutic agents is proposed, taking into account the peculiarities of the functional groups and substituents of each compound. The conjugate synthesis strategy is based on the interaction of pre-functionalised A3B-type *meso*-arylporphyrins with functionally active substituents (hydroxy-, carboxy-, amino-, pyridyl-) and derivatives of 4′-(4-methylphenyl)-2,2′:6′,2′-terpyridine (bromo-, amino-, carboxy-) (Scheme 1). The formation of the amide bond or alkylation reaction was chosen as the main strategy for obtaining the conjugates.

**Scheme 2:**
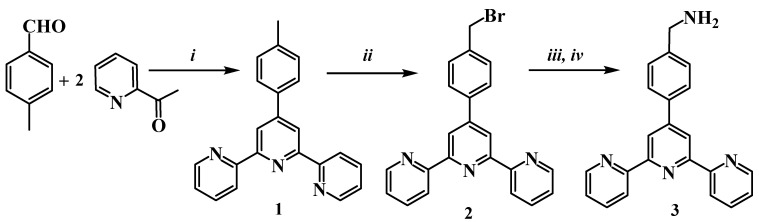
Synthesis of terpyridine and its derivatives.

For the first step, the preparation of 4′-(4-methylphenyl)-2,2′:6′,2″-terpyridine **1** and its derivatives **2**–**3** was redesigned. Recently, a variation of the Kröhnke method was proposed [37,38,39]. The overall yield of the target compound **1**, according to two of these papers [38,39], was 19% and 63%, respectively. After the adaptation of the synthetic procedure of compound **1**, its yield increased to 75%. Thus, 2-acetylpyridine and 4-methylbenzaldehyde were first dissolved in ethanol, and then 85% KOH in an aqueous ammonia solution was added to the resulting mixture and stirred for 4 h at a slightly increased temperature. The resulting product was precipitated from the reaction mixture and was then filtered and washed with cold ethanol and recrystallised from ethanol (Scheme 2).

**Reagents and reaction conditions**: (*i*) KOH, NH_3_, EtOH, t = 34 °C; (*ii*) NBS, AIBN; CCl_4_, t = 77 °C; (*iii*) NaN_3_, DMF, Ar, t = 70 °C; (*iv*) PPh_3_, THF, H_2_0.

Bromosubstituted derivative **2** was prepared by a radical bromination reaction with N-bromosuccinimide in the presence of AIBN, according to a method described in the literature [40]. In the ^1^H-NMR spectrum of compound **2**, the singlet signal from the Tpy methyl group disappeared at 2.36 ppm and a singlet from CH_2_Br appeared at 4.49 ppm. (SI, Figures S1–S12).

For the 4′-(4-aminomethylphenyl)-2,2′:6′,2″-terpyridine **3**, the proposed strategies that included interaction with concentrated ammonia solution and Gabriel reaction produced low yields [38,41]. As a result, for compound **3**, interaction with sodium azide, followed by a mild Staudinger reduction, was chosen. Thus, bromide **2** and sodium azide were dissolved in DMF and stirred at 70 °C in an argon flow for 8 h without access to light. In the IR spectrum of the azide compound, characteristic bands for aryl azids were observed at 2.088 cm^−1^ (asymmetric valence vibrations) and 1.287 cm^−1^ (symmetric valence vibrations), corresponding to the 4′-(4-azidomethylphenyl)-2,2′:6′,2′-terpyridine azide group (SI, Figure S9). In addition, an amine was obtained from the azide in a 75% yield by the Staudinger reaction.

**Scheme 3:**
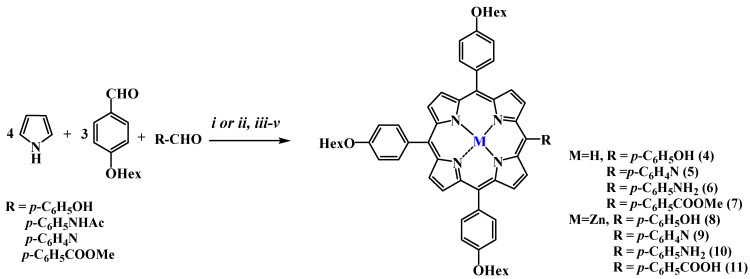
Synthesis of porphyrins and their complexes with Zn.

*Preparation of meso-arylporphyrins of A3B type and their metal complexes*. Earlier, the authors demonstrated the efficiency of mixed-aldehyde monopyrrole condensation by the Adler and Lindsey methods, using previously obtained substituted benzaldehydes to obtain A3B-type porphyrins with functionally active substituents [42,43]. In the condensation reaction with pyrrole, two corresponding substituted aldehydes were used in stoichiometric ratios, yielding a mixture of six porphyrin products. The target free-base A3B porphyrins were isolated by column chromatography with a maximum yield of up to 13% (Table 1, Scheme 3). Each type of porphyrin required a specific synthetic technique, depending on the lability of the starting groups. Thus, for the synthesis of porphyrins containing hydroxyl, pyridyl, or amino groups, the improved Adler method in three solvents- nitrobenzene, acetic and propionic acids—is optimal [43,44]. This approach involves the use of propionic and acetic acids as the solvents and nitrobenzene as an additional oxidant. A mixture of carboxylic acids and nitrobenzene was boiled for 15 min, then aldehydes were added, following the slow addition of pyrrole to nitrobenzene. The products of monopyrrole condensation were separated by column chromatography. The yields of the target compounds are given in Table 1. For the synthesis of porphyrin **8** with a carboxylic group, the Lindsey method was used to obtain intermediate free-base porphyrin, followed by the removal of the methyl-protecting group [45].

**Reagents and reaction conditions**: For porphyrins, **4**–**6**: (*i*) acetic acid, propionic acid, nitrobenzene (1:2:1, *v*/*v*); boiling; 2 h; for porphyrin **7**: (*ii*) CHCl_3_, BF_3_ OEt_2_, DDQ, rt; for porphyrin **5** (removal of acetyl protection): (*iii*) TFA/HCl; boiling; 4 h; (*iv*) ZnOAc_2_, MeOH; CHCl_3_; for porphyrin **11**: (*v*) THF, EtOH, H_2_O (4:2:1, *v*/*v*); KOH; boiling.

Zinc metal complexes **8**–**11** were obtained using the standard method, by adding the corresponding metal salt to the porphyrin in the methylene/methanol system. The reaction was monitored using electron absorption spectra (EAS). The structure and identity of the obtained compounds were confirmed by TLC, ^1^H- and ^13^C-NMR- and UV-spectroscopy, HPLC-HRMS, and MALDI-TOF mass spectrometry (SI, Figures S13–S35).

**Scheme 4:**
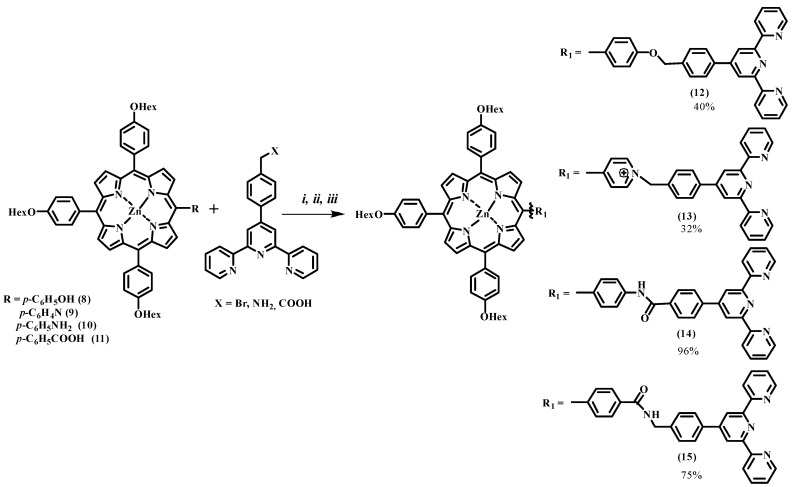
Synthesis of conjugates.

*Preparation of conjugates of meso-arylporphyrins and derivatives of 4′-(4-methylphenyl)-2,2′:6′,2*″*-terpyridine*. The target *meso*-arylporphyrins with terpyridine moieties were synthesised according to two different strategies: by O- or N-alkylation reactions, as well as the creation of an amide bond. Thus, conjugates **12**–**13** were obtained by the alkylation of Zn(II) complexes of porphyrins **8**, **9** with 4′-(4-aminomethylphenyl)-2,2′:6′,2″-terpyridine **2** (Scheme 4). This reaction was carried out in DMF (for **12**) or THF (for **13**), with an excess (8 eq.) of the base as a catalyst to increase the reaction rate and yield. It should be noted that the use of Cs_2_CO_3_ as a catalyst resulted in a significantly greater yield than with KOH, DBU, or K_2_CO_3_. The yield of conjugate **12** increased to 40%. The yield of the N-alkylation reaction was lower, so compound **13** was isolated in 32%. Apparently, such a yield in this reaction is related to steric hindrance in the porphyrin molecule. The target compounds **12**–**13** were purified by column chromatography on aluminum oxide, followed by recrystallisation in acetonitrile.

**Reagents and reaction conditions**: (*i*) DMF, Cs_2_CO_3_, Ar; (*ii*) THF, EDC, DMAP, HOBT, Ar; t = 0 °C.

The next type of amide-bonded conjugate **15** was obtained according to the carbodiimide method, with nucleophilic additives using EDC and DMAP in the presence of stoichiometric amounts of HOBT in a 75% yield (Scheme 4) [46]. In order to improve the yield of this reaction, the synthesis of conjugate **14** acylation by Schotten-Baumann was used. Thus, compound **10** was treated with chlorohydride of 4-([2,2′:6′,2′-terpyridine]-4′-yl) benzoic acid [47] in the presence of Et_3_N. The yield of the acylation reaction was almost quantitative (96%). The identity and chemical structure of the obtained conjugates **12–15** were confirmed by thin layer chromatography, EAS, ^1^H- and ^13^C-NMR spectroscopy, ^1^H-^1^H COSY, mass spectrometry and HPLC-HRMS (SI, Figures S36–S48). In ^1^H-^1^H COSY spectrum of conjugate **12** signals from the terpyridine fragment are observed: at 8.82 ppm (2H, singlet, 3′5″), 8.75 ppm (2H, doublet multiplet, 6′6″), 8.71 ppm (2H, multiplet doublet, 3′3″), 7.90 ppm (2H, multiplet, 5.5′) and 7.36 ppm (2H, multiplet, 4,4′) (Figure S37 of Supporting Information). Signals from protons of OCH_2_- or N^+^CH_2_ groups are observed as singlets at 5.29 and 6.16 ppm, respectively. In the ^13^C-NMR spectrum of porphyrin **15**, there is a signal in the weak field region of 168.90 ppm corresponding to the carbon atom of the amide group (SI, Figures S36–S48). For the obtained conjugates, **12**–**15** HPLC HRMS analysis was also performed, confirming the formation of desired compounds. 

**Figure 1:**
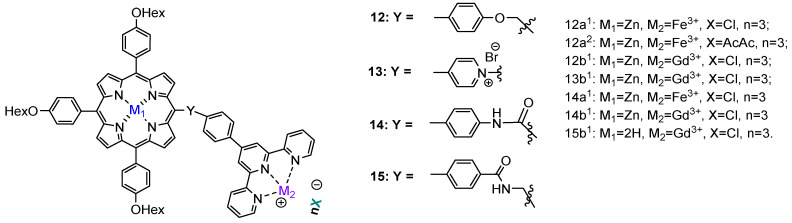
The structure of the obtained complexes.

*Complexation of conjugates with paramagnetic metals*. The chelating properties and reaction condition of conjugates **12**–**15** with paramagnetic Fe(III) and Gd(III) were established and a series of complexes were obtained (Table 2, Figure 1). It was found that Fe(III) complexes **12**–**15a^1,2^** were formed under mild conditions, supporting previous research [48]. The reaction mixture was stirred in an inert atmosphere at room temperature, with the addition of sodium acetate as a catalyst in the methylene/methanol system. Anhydrous chloride or acetylacetonate metal salts were used. During the complexation process, the target compounds precipitated, the products were purified by extraction, and subsequent recrystallisation from acetonitrile was followed by drying under vacuum conditions.

The introduction of Gd(III) required more extreme conditions of reaction conducting In the absence of temperature, the yields of the reaction were 6–10% (Table 2). In order to improve the low yields, the reaction was carried out at high pressure while heating (80 °C) for 36–54 h. Conjugates **12**–**15b^1,2^** were dissolved in THF and anhydrous GdCl_3_ was added to methanol in an inert atmosphere, after which anhydrous sodium acetate (2.5 eq) was added (Table 2, Figure 1). The degree of transformation was evaluated by TLC on aluminum oxide. Target compounds were precipitated from the solution; then, the precipitate was centrifuged, washed with acetonitrile and methanol, and recrystallised in THF/acetonitrile system. The introduction of gadolinium ions increased the hydrophilicity of the molecule to such an extent that the target compounds were isolated by RP chromatography. The structure and identity of the compounds obtained were confirmed by TLC, IR, EPR spectroscopy, and MALDI-TOF mass spectrometry. In the IR spectra, a change in the strain vibrations of bands belonging to the terpyridine residues at 1.545 cm^−1^, 1.559 cm^−1^, 1.576 cm^−1,^ and 1.606 cm^−1^ was observed. The broad absorption bands ν (C = C) of the free ligand around 1.576 cm^−1^ shifted towards higher wave numbers compared with the complexes, indicating coordination of the nitrogen atoms in Tpy with the transition metal ion, which is consistent with previous work [24]. In the MALDI-TOF mass spectra of the complexes, peaks corresponding to the fragmentation of the target compound were observed (SI, Figures S49–S55). The resulting molecular and fragment ion signals were described and identified. Signals from paramagnetic gadolinium and iron atoms were also observed in the EPR spectra.

**Figure 2:**
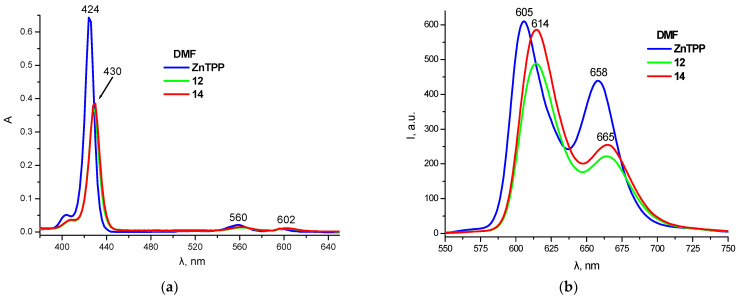
UV-Vis absorption (**a**) and fluorescence (**b**) spectra of ZnTPP, **12** and **14** in DMF (*C* = 0.75 μM).

*Photophysical and photochemical properties of compounds **12** and **14***. Spectral parameters, photophysical properties, and photochemical activity were studied for conjugates **12** and **14**, as the most promising candidates in terms of the yield of the target compounds. For zinc complexes **12** and **14** in DMF, the main parameters of absorption and emission spectra were very similar, with λ_B_ = 428–430 nm and λ_Q_ = 560 and 602 nm (Figure 2a,b). A bathochromic shift of the absorption maxima by 5–6 nm relative to the unsubstituted ZnTPP was due to the presence of substituents in meso- positions of the macrocycle. Molar absorption coefficients for **12** and **14** were found to be 1.5 times lower than for ZnTPP (Table 3).

**Figure 3:**
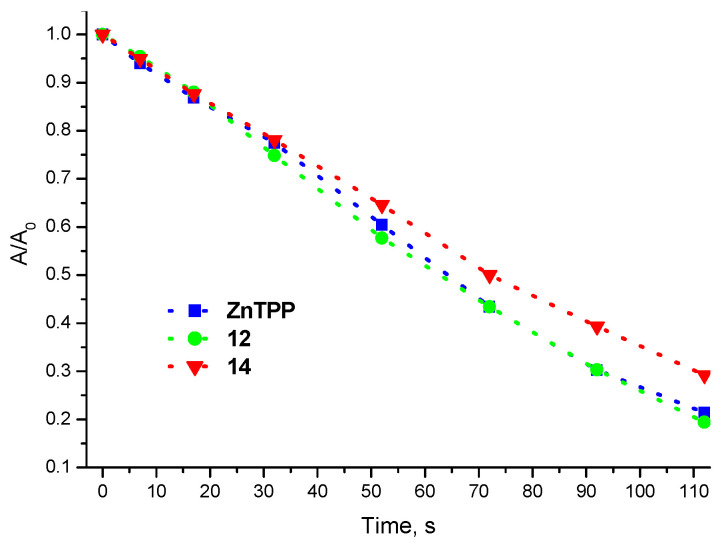
Kinetic curves of DPBF oxidation by singlet oxygen photosensitized by ZnTPP, **12** and **14**.

Fluorescence spectra of compounds **12** and **14** were also very similar in shape and maxima position but differed significantly in the component ratio from the emission spectra of ZnTPP, and were also characterised by a bathochromic band shift (7–9 nm) (Figure 2b). At the same time, complexes **12** and **14** demonstrated slightly higher fluorescence quantum yields (Φ*_F_* = 4–5%). A comparison of the photochemical activity of compounds **12** and **14** in DMF revealed very close values of singlet oxygen quantum yields, of about 65% (Figure 3).

In contrast to organic solvents, in a polar aqueous medium all the compounds studied exhibited an increased tendency to self-assemble into non-specific aggregates that differed from the porphyrin monomolecular form by a significant decrease in the molar absorption coefficient (by a factor of 2.5–3.5 compared with that in DMF), an increase in the half-width of the Soret band from 10 to 14 nm to 36–40 nm and almost complete fluorescence quenching. In order to stabilise the photoactive monomolecular form of porphyrins, their aggregation state, and spectral properties were compared in aqueous solutions of various solubilisers.

**Figure 4:**
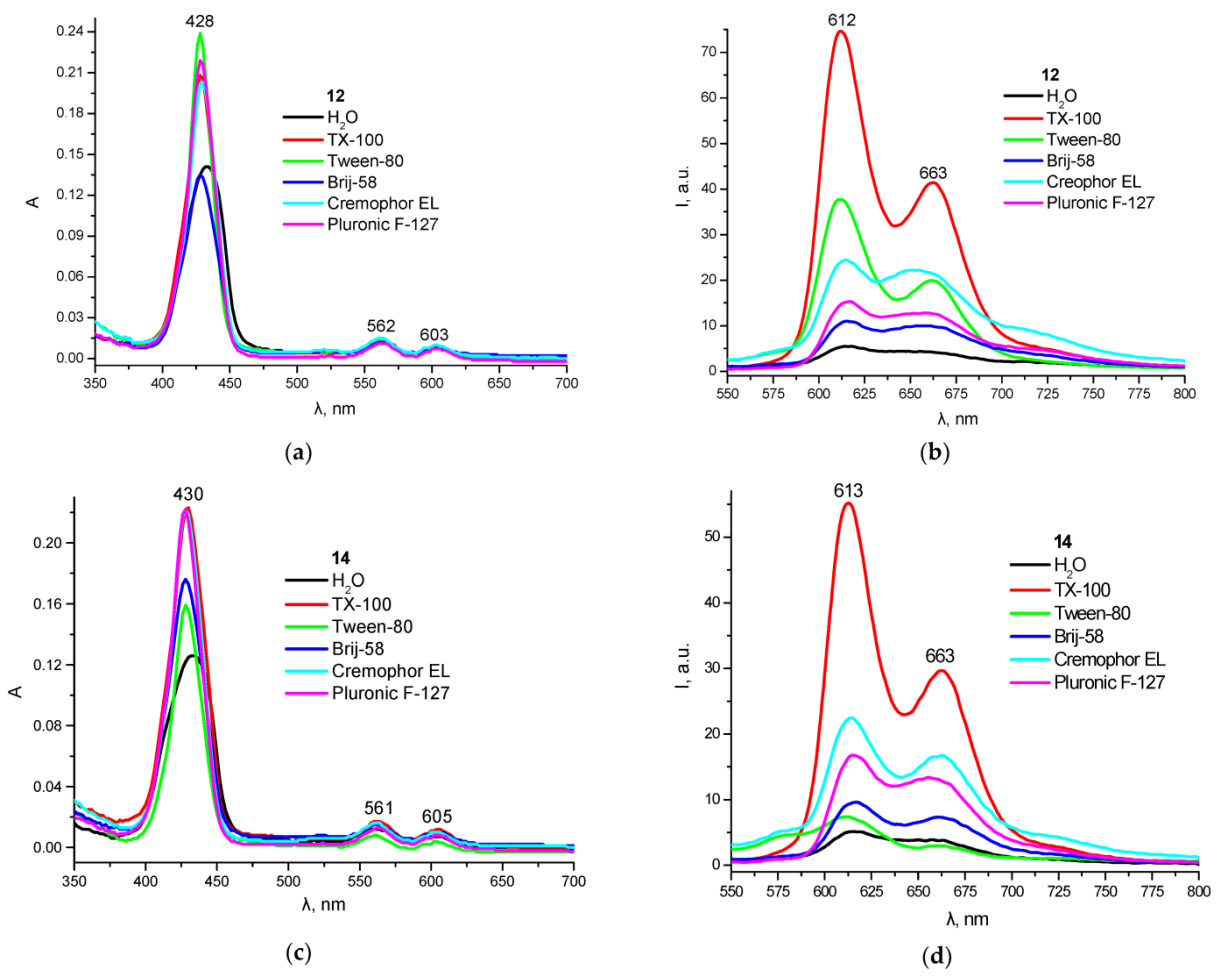
UV-Vis absorption (**a**,**c**) and fluorescence (**b**,**d**) spectra of **12** (**a**,**b**) and **14** (**c**,**d**) in water and aqueous solutions of different solubilizing agents (*C* = 0.9 ± 0.05 μM).

For zinc complex **12**, nonionic surfactant Triton X-100 (TX-100) was the most efficient solubiliser. However, even in a micellar TX-100 solution, a fluorescence intensity of **12** was an order of magnitude lower than in DMF, indicating a relatively low level of solubilisation. In addition to TX-100, partial solubilisation of **12** was observed in the presence of 0.5 mM Tween-80, 1% Cremophor EL and 1% Pluronic F-127. In the latter two cases, a significant increase in the intensity of the long-wavelength band in the emission spectra was observed, which is not typical for the free monomolecular form of porphyrins (Figure 4a,b, Table 3). For compound **14**, the results of solubilisation studies were similar to those for complex **12**, except for the lower efficiency of Tween-80. The least effective solubiliser, as in the previous case, was a 0.5 mM solution of a nonionic surfactant Brij-58 (Figure 4c,d, Table 3).

Thus, according to the results of solubilisation studies, the most effective stabilisation of the fluorescent monomolecular form of porphyrins **12**, **14** was observed in a micellar solution of a synthetic nonionic surfactant TX-100 at 1 mM concentration. Among the biocompatible solubilisers, 1% Cremophor EL and 1% Pluronic F-127 were the most effective for both complexes studied.

*Characterisation of micellar PS formulations*. Micelles are one of the most popular drug delivery vehicles [49,50]. Pluronic F127 is a block copolymer of polyoxyethylene (PEO) and polyoxypropylene (PPO). The wide use of Pluronic F127 is due to its non-toxicity, biocompatibility, and stability [50,51]. Recently [42], the authors have shown that the incorporation of cationic *meso*-arylporphyrins in Pluronic F-127 micelles reduces the PS dose by a factor of two without light exposure and by more than a factor of four when irradiated in the antimicrobial photodynamic therapy study. According to the authors’ previously reported data and the physicochemical properties observed, polymeric micelles of Pluronic F127 were selected as potential biocompatible dosage forms for the biological tests. The solid dispersion method was chosen for obtaining micelles [52,53]. The solubilisation of conjugates **12**, **14** in Pluronic F127 (2.5%, *w*/*v*) was carried out in THF, with subsequent rotatory evaporation of the solvent; this resulted in a thin solid film that could be dissolved in aqueous systems. The micelles obtained were characterised by dynamic light scattering, and a narrow particle size distribution (100–150 nm) was observed (SI, Figures S56–S57). These results differ from those of the authors’ previous work. In the case of cationic porphyrins, the size of the nanoparticles obtained was 19–25 nm. Apparently, the size of the colloidal structures formed depends on the hydrophobicity of the porphyrins used.

### 3.1. Evaluation of Biological Efficacy and Safety

In the present study, the potential use of newly synthesised compounds in medicine was evaluated using three methods. The cytotoxicity of the compounds was determined using the MTT test for two cell lines: tumour (HEP-2) and conditionally normal (NKE), both under irradiation and non-irradiation conditions. In addition, two basic preclinical tests of potential drugs aimed at detecting hidden carcinogenic properties of compounds were performed, namely, Comet assay (DNA damage test) and Gap junction-mediated intercellular communication test (evaluation of non-genotoxic component of carcinogenesis).

**Figure 5:**
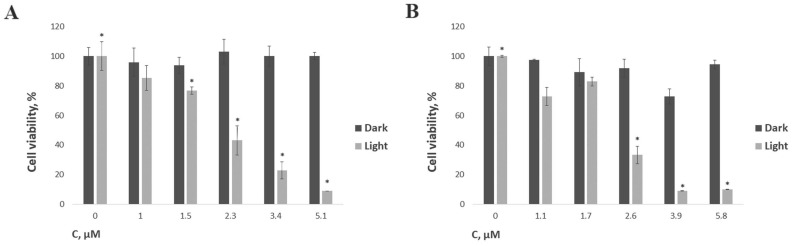
The effect of compounds on the viability of Hep2 cell line. Cells were irradiated for 90 min using the Medical Therapy Philips TL 20W/52 lamp (irradiation dose of 8.073 J/cm^2^). Incubation of cells with the compound without irradiation in the dark for 90 min. * statistically significant differences in cell survival relative to the values with zero concentration of the compound (*p* < 0.01) were noted. (**A**) conjugate **12**; (**B**) conjugate **14**.

*Cell viability study (MTT-test)*. The cytotoxicity of compounds **12** and **14** was studied on a Hep2 cell line (human laryngeal cancer) and NKE (human normal kidney epithelial), with and without irradiation. Two approaches were used when treating the cells. In the first case, compounds **12** and **14** were introduced into the cell culture only for the irradiation time (90 min). After that, the cell medium containing one of the compounds was removed. The cells were incubated in a fresh DMEM medium without compounds for a further 24 h, after which time the results were evaluated. The IC_50_ values were close for both cell lines (Table 4, Figure 5 and Figure S58). As a result, for the Hep2 cells the IC_50_ of compound **12** was shown to be 1.87 ± 0.333 μM when irradiated for 90 min (in Hanks’ solution) and with an irradiation dose of 8.073 J/cm^2^; without irradiation, the compound had no toxic effect in the concentration range studied (Figure 5). For compound **14**, the IC_50_ was 1.4 ± 0.152 μM and it demonstrated no toxicity when incubated without light. The results for the NKE cell line are presented in Table 4 and Figure S58 (Supporting Information).

In the second series of experiments, cell viability was evaluated 24 h after irradiation without removing the compound from the culture medium. As a result, for the Hep2 cells, conjugate **12** was shown to have an IC_50_ of 2.47 ± 0.233 μM (8.073 J/cm^2^ irradiation dose) and 2.55 ± 0.28 μM (no irradiation) after 24 h. For conjugate **14**, the corresponding values of IC_50_ were 1.55 ± 0.15 μM (irradiation dose 8.073 J/cm^2^) and 4.17 ± 0.251 μM (no irradiation). The results for the NKE cell line are presented in Table 4.

The obtained data shows significant dark cytotoxicity of compounds **12** and **14** when cells are incubated with the compounds for 24 h. However, the received IC50 values are characteristic of the porphyrins, which most often lie in the range of 1 to 30 μM [54,55,56]. Unfortunately, the IC50 value of compound **12** does not change depending on the presence or absence of irradiation for both cell lines. For compound **14**, the IC50 decreases by 2–2.5 times with irradiation compared to the dark effect. This difference is significantly lower compared to the DPP-ZnP-(GdDOTA)2 compound, where the IC50 value was reduced 6 times when irradiated [54]. It should be noted that Jenni et al. used a much higher total irradiation dose of 45 J/cm^2^, whereas in our work 8.073 J/cm^2^. Possibly, increasing the total irradiation dose in the case of compound **14** will lead to a more significant difference between the IC50 values of the dark effect and after irradiation.

The short-term treatment of cell lines with compounds **12** and **14** gave more promising results. In this case, the cytotoxicity upon irradiation is achieved at non-toxic concentrations without irradiation (Figure 5). Thus, the received compounds can potentially be used in superficial neoplasms (dermatology and gynaecology) where the application of the compounds is possible, followed by their removal from tissues after irradiation.

*Gap junction-mediated intercellular communication test*. In the present study, the metabolic cooperation inhibition test was used to determine the degree of disruption of intercellular contacts. Intercellular gap junctions are designed to regulate normal cell growth and differentiation. Disruption of the intercellular contacts leads to many pathological processes, such as carcinogenesis or the development of hereditary diseases [54]. PDT drugs are often used locally in the form of applications, so the evaluation of the ability to disrupt the integrity of intercellular contacts (test for inhibition of metabolic cooperation or promoter activity) was established. For this purpose, the well-proven method of introducing a fluorescent indicator with a scratch into the IAR2 (rat liver epithelium) cell monolayer and then tracking its movement perpendicular to the scratch was applied [57,58]. During the experiment, IAR2 was seeded into 24-well plates. After reaching the monolayer, compounds **12** and **14** were added at concentrations ranging from 0.56 to 4.5 μM and incubated for 4 h. TPA (12-O-tetradecanoylphorbol-13-acetate) was used as a positive control as a classic promoter of carcinogenesis [59]. After 4 h, the medium with the compounds was removed and the fluorescent indicator Lucifer yellow (LY) was introduced into the cell monolayer by scratching. The result was evaluated using a Zeiss fluorescence microscope with a phototip (Axioplan 2 imaging) filter for FITC fluorescence.

**Figure 6:**
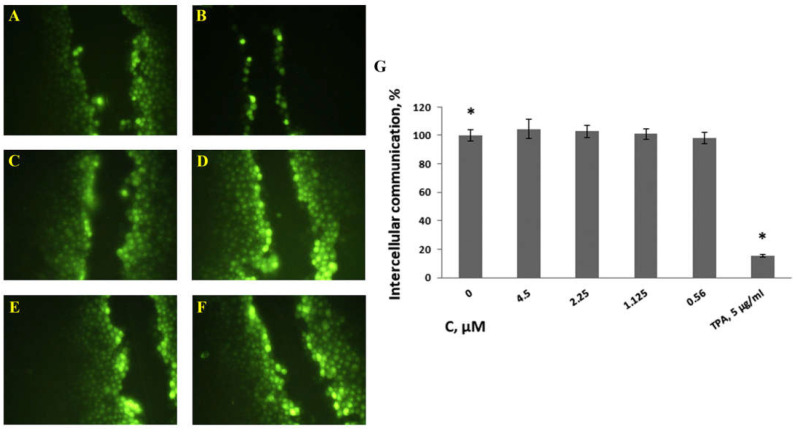
Micrographs of the cell monolayer in the scratch area of the control and experimental samples. (**A**). Negative control (dH_2_O). (**B**). Positive control (TPA, 5 μg/mL). Compound **12**: 4.5 μM (**C**); 2.25 μM (**D**); 1.125 μM (**E**) and 0.56 μM (**F**). LY propagation length in 4 min. Histogram of the cells proportion in cooperation with compound **12** at concentration: 104% at 4.5 μM, 103% at 2.25 μM, 101% at 1.125 μM, and 98% at 0.56 μM (* statistically significant difference with negative control, *p* < 0.01) (**G**).

**Figure 7:**
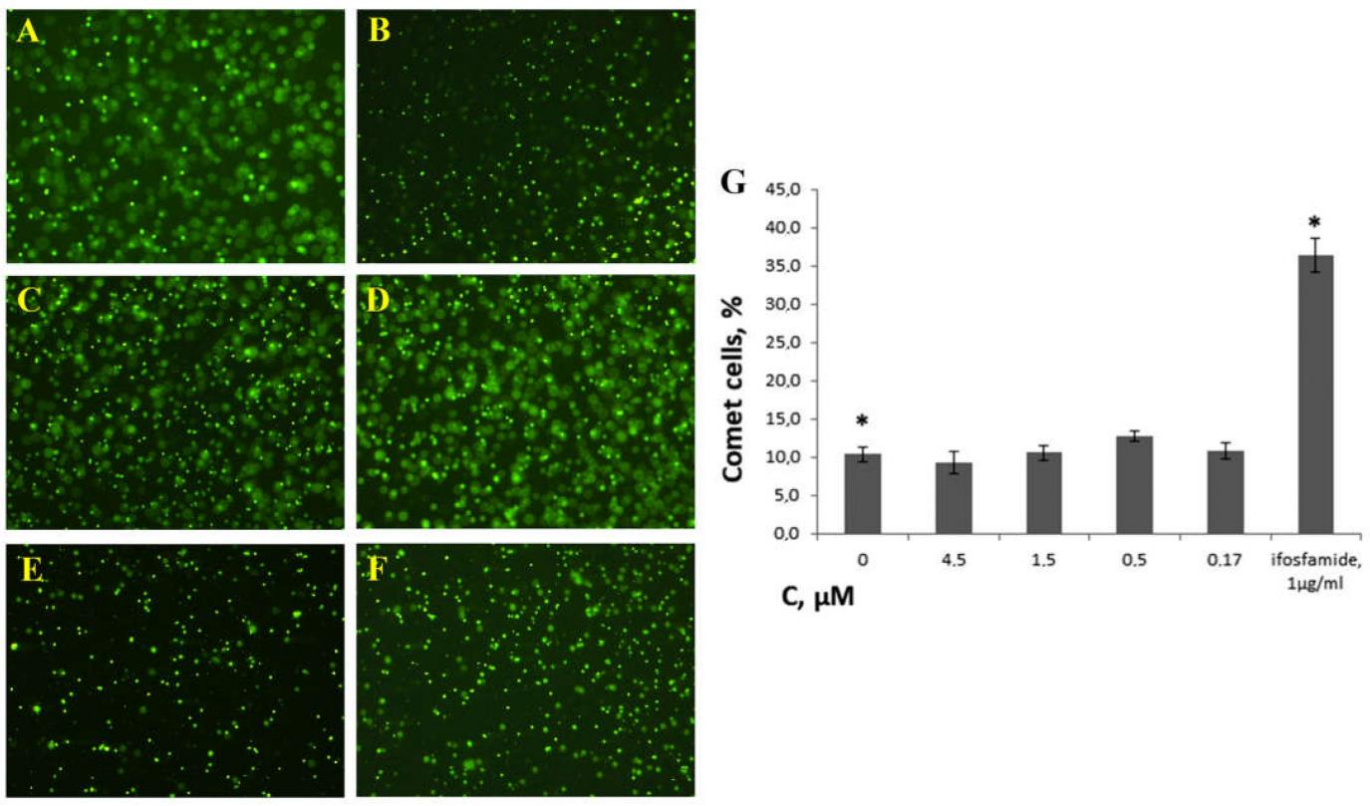
Effect of compound **14** on the number of ‘comet’ cells. NKE cells were incubated with the compound at final concentrations of 4.5 μM (**A**), 1.5 μM (**B**), 0.5 μM (**C**), and 0.17 μM (**D**) for 24 h. The negative control was physiological solution (**E**) and the positive control was ifosfamide 1 μg/mL (**F**). Histogram of the dependence of the comet cells proportion on the concentration of compound **14** (**G**). The proportion of ‘comet’ cells for the negative control population was 10.3 ± 1.03%; for conjugate **14** at concentration 4.5 μM, the proportion was 9.3 ± 1.39%; at concentration 1.5 μM, the proportion was 10.5 ± 0.89%; at concentration 0.5 μM, the proportion was 12.7 ± 0.74%; at concentration 0.17 μM, the proportion was 10.8 ± 1.03%. The proportion of ‘comet’ cells in the population treated with ifosfamide 1 µg/mL was 36.1 ± 2.14%, (* statistically significant difference with negative control, *p* > 0.01).

The study demonstrated that compounds **12** and **14** did not exhibit promoter activity in the given concentration ranges, i.e., they did not disrupt intercellular contacts. Thus, when exposed to compound **12** at concentrations of 4.5 μM, 2.25 μM, 1.125 μM, and 0.56 μM, the degree of cell cooperation was 104%, 103%, 101%, and 98% with respect to the untreated cells (Figure 6). For **14**, these values were 109%, 105%, 101%, and 111%, respectively (SI, Figure S58). At the same time, the degree of cell cooperation in the samples treated with TPA at a final concentration of 5 μg/mL varied from 15% to 20%, which was statistically significantly different from the controls (*p* < 0.01). Figure 7 shows microphotographs of the cell monolayer in the scratch area from both the control and experimental samples. Both **12** and **14** compounds showed efficacy and safety in a given concentration range. The conjugates obtained did not damage the intercellular contacts, and thus did not promote tumour transformation in healthy tissues.

### 3.2. Comet Assay

The Comet assay (OECD 489) was used to evaluate the possible genotoxicity of compound **14**. During the study, the NKE cells were seeded in 24-well plates. After the cells reached 70% coverage, compound **14** was added in the concentration range from 0.17 to 4.5 μM and left for 24 h. After this time, the assay itself was performed using OxiSelect™ Comet Assay Kit (Cell Biolabs, San Diego, CA, USA). When analysing the degree of DNA damage in the DNA comet assay, the proportion of ‘comets’ in the total number of cells was estimated. It was shown that treatment of NKE cells with the compound **14** at final concentrations of 4.5 µM, 1.5 µM, 0.5 µM, and 0.17 µM did not increase the degree of DNA damage (Figure 7). At the same time, the drug ifosfamide (Baxter Oncology, Halle, Germany), a known DNA-damaging agent (positive control), caused a significant increase in the number of ‘comets’, compared with the control group, (36.4% of the total number of cells, compared with 10.3% in the negative control). 

Thus, it is shown that compound **14** has no direct genotoxic effect despite its significant cytotoxicity. Most likely, its cytotoxicity is due to the interaction with DNA and changes in the expression profile of a number of genes, which is characteristic of porphyrin-based compounds [60]. In particular, porphyrins are known to act as stabilising agents against noncanonical DNA structures, G-quadruplexes, often located in the promoter parts of genes, which leads to changes in the expression profile of the latter [61,62].

## 4. Conclusions

The greatest advantage of metalloporphyrins is their multifunctionality, simultaneously allowing the conjugation and chelation of metals, in many cases retaining their inherent fluorescent and therapeutic properties. Conjugation with terpyridine fragments is a convenient strategy for introducing metals with large atomic radii. Photophysical and photochemical studies of the obtained conjugates revealed properties typical of the porphyrin moiety with good fluoresce and excellent singlet oxygen generation. Solubilisation studies enabled the suggestion of a micellar form of the target compounds suitable for application in aqueous biological media. In our work, we showed the influence of structure fragments on their biological activity. Thus, it was shown that the amide bond between the macrocycle and terpyridine fragment in conjugate **14** leads to the decrease of IC50 values by 2–2.5 times with irradiation compared to the dark effect under 24 h experiment. Preliminary in vitro studies have shown the safety and efficacy of compounds **12** and **14** in their short-term exposure. Hence, such conjugates can potentially be considered a new type of theranostic agent when considering the application method of use with their subsequent removal from the tissues. Therefore, the proposed design of new theranostic agents opens the way for further functionalisation to increase the therapeutic window during long-term exposure to porphyrin-type compounds.

## Figures and Tables

**Table 1:** The yield of asymmetric *meso*-arylporphyrins of A3B type.

Compound	R1	M	η, %
**4**	-*p*C_6_H_5_OH	H	12.6
**5**	-*p*C_6_H_5_NH_2_	H	12
**6**	-*p*C_6_H_4_N	H	11
**7**	-*p*C_6_H_5_COOMe	H	13
**8**	-*p*C_6_H_5_OH	Zn	95
**9**	-*p*C_6_H_4_N	Zn	90
**10**	-*p*C_6_H_5_NH_2_	Zn	92
**11**	-*p*C_6_H_5_COOH	Zn	95

**Table 2:** Reaction condition and the yields of the metal complexes.

Compound	Salt of the Metal	Catalyst	Conditions	Yield, η%
**12a^1^**	FeCl_3_	-	CH_2_Cl_2_, MeOH, t_KOMH._, N_2_	75
**12a^2^**	Fe(AcAc)_3_	NaOAc	CH_2_Cl_2_, MeOH, t_KOMH._	82
**12b^1^**	GdCl_3_	NaOAc	THF, MeOH, t., p, N_2_	36
**13b^1^**	GdCl_3_	NaOAc	THF, MeOH, t_KOMH._, p, N_2_	6
**14a^1^**	FeCl_3_	-	CH_2_Cl_2_, MeOH, t_KOMH._, N_2_	70
**14b^1^**	GdCl_3_	NaOAc	THF, MeOH, t., p, N_2_	30
**15b^1^**	GdCl_3_	NaOAc	THF, MeOH, t., p, N_2_	28

**Table 3:** Spectral parameters of **12** and **14** in aqueous solutions of different solubilising agents.

**12**	**DMSO**	**H_2_O**	**TX-100**	**Brij-58**	**Cremophor**	**Pluronic**	**Tween-80**
**λ_max_**	428	433	428	428	430	428	428
**lg*ε***	5.70	5.17	5.34	5.15	5.35	5.36	5.40
**Δλ_B_, nm**	10	36	25	28	27	22	19
**λ_em_, nm**	614, 664	614, 660	612, 663	615, 660	614, 655	617, 658	612, 662
***I_1_*** **/*I*_2_**	2.20	0.39	1.83	1.10	1.14	1.15	1.90
**14**	**DMSO**	**H_2_O**	**TX-100**	**Brij-58**	**Cremophor**	**Pluronic**	**Tween-80**
**λ_max_**	430	433	430	428	428	428	428
**lgε**	5.71	5.17	5.44	5.32	5.4	5.41	5.27
**Δλ_B_, nm**	10	40	31	31	26	24	25
**λ_em_, nm**	614, 665	615, 664	613, 663	617, 661	614, 662	615, 660	612, 661
***I*** **_1_** **/*I*_2_**	2.30	1.25	1.83	1.43	1.29	1.31	2.33

**Table 4:** IC_50_ of compounds **12** and **14** for the Hep2 and NKE cell lines.

	Compound	12 (Light Irradiation *)	12 (Dark Conditions)	14 (Light Irradiation *)	14 (Dark Conditions)
Time					
Hep2 cell line					
**1.5 h**		1.87 ± 0.333 µM	-	1.4 ± 0.152 µM	-
**24 h**		2.47 ± 0.233 µM	2.55 ± 0.28 µM	1.55 ± 0.15 µM	4.17 ± 0.251 µM
NKE cell line					
**1.5 h**		1.7 ± 0.297 µM	-	2.0 ± 0.34 µM	-
**24 h**		2.57 ± 0.32 µM	2.44 ± 0.281 µM	1.8 ± 0.189 µM	3.85 ± 0.26 µM

* Radiation dose 8.073 J/cm^2^.

## Data Availability

Not applicable.

## Supplementary Materials

The following supporting information can be downloaded at: https://www.mdpi.com/article/10.3390/pharmaceutics15010269/s1, Supplementary File: Spectral data of synthesised compounds, physicochemical properties, solubilization study and biological activity.
