# Supplementary material for: Design of A3B-Porphyrin Conjugates with Terpyridine as Potential Theranostic Agents: Synthesis, Complexation with Fe(III), Gd(III), and Photodynamic Activity
Source: Pharmaceutics. 2023 Jan 12;15(1):269. doi: 10.3390/pharmaceutics15010269 (PMC9865040; doi:10.3390/pharmaceutics15010269)
Supplement: Supplementary file 1 [file pharmaceutics-15-00269-s001.zip › pharmaceutics-2000366-supplementary.pdf]

Supporpting information for

Design of A3B-Porphyrin Conjugates with Terpyridine as Potential Theranostic Agents: Synthesis, Complexation with Fe(III), Gd(III), and Photodynamic Activity

Kseniya A. Zhdanova [1,*], Anastasia V. Ivantsova [1], Fedor Yu. Vyalba [1], Maxim N. Usachev [1], Margarita A. Gradova [2], Oleg V. Gradov [2], Natalia Yu. Karpechenko [3,4] and Natal'ya A. Bragina [4]

[1] Institute of Fine Chemical Technologies, MIREA—Russian Technological University, 119571 Moscow, Russia
[2] N.N. Semenov Federal Research Center for Chemical Physics, Russian Academy of Sciences, Kosygin Street 4, 119991 Moscow, Russia
[3] N.N. Blokhin National Medical Research Center of Oncology, Ministry of Health of Russia, 115522 Moscow, Russia
[4] Ministry of Health of Russia, Pirogov National Research Medical University, 117997 Moscow, Russia
* Correspondence: zhdanova_k@mirea.ru

Table of contents

**1.** *^{1}H NMR, ^{13}C NMR, MALDI-TOF spectra of terpyridine derivatives* ***1-3***	S1-12
**2.** *^{1}H NMR, ^{13}C NMR, mass-spectra data for porphyrins* ***4-11***	S13-35
**3.** *^{1}H NMR, ^{13}C NMR, mass-spectra data for conjugates* ***12-15***	S36-48
**4.** *Mass-spectra data for complexes* ***12-15***	S49-55
**5.** *Particle size distribution*	S56-57
**6.** *Cell viability study (MTT - test) on NKE cells.*	S58
**7.** *Influence on the time/dose irradiation*	S59
**8.** *Gap junction-mediated intercellular communication test*	S60

^{1}H NMR, ^{13}C NMR, MALDI-TOF spectra of terpyridine derivatives ***1-3*** are illustrated below (see **Figures** S1-12).

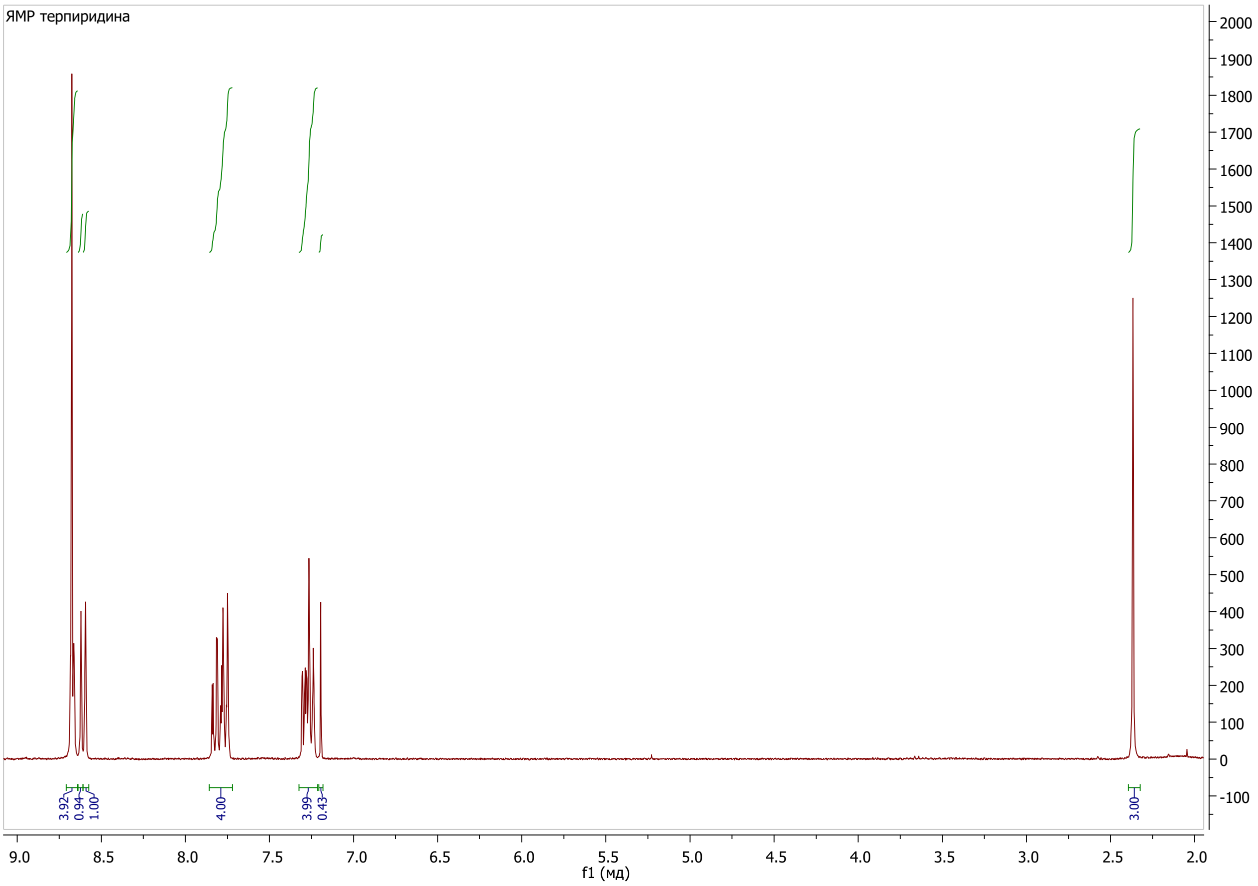

Figure. S1. ^{1}H-NMR spectrum ($CDCl_3$) of compound **1**

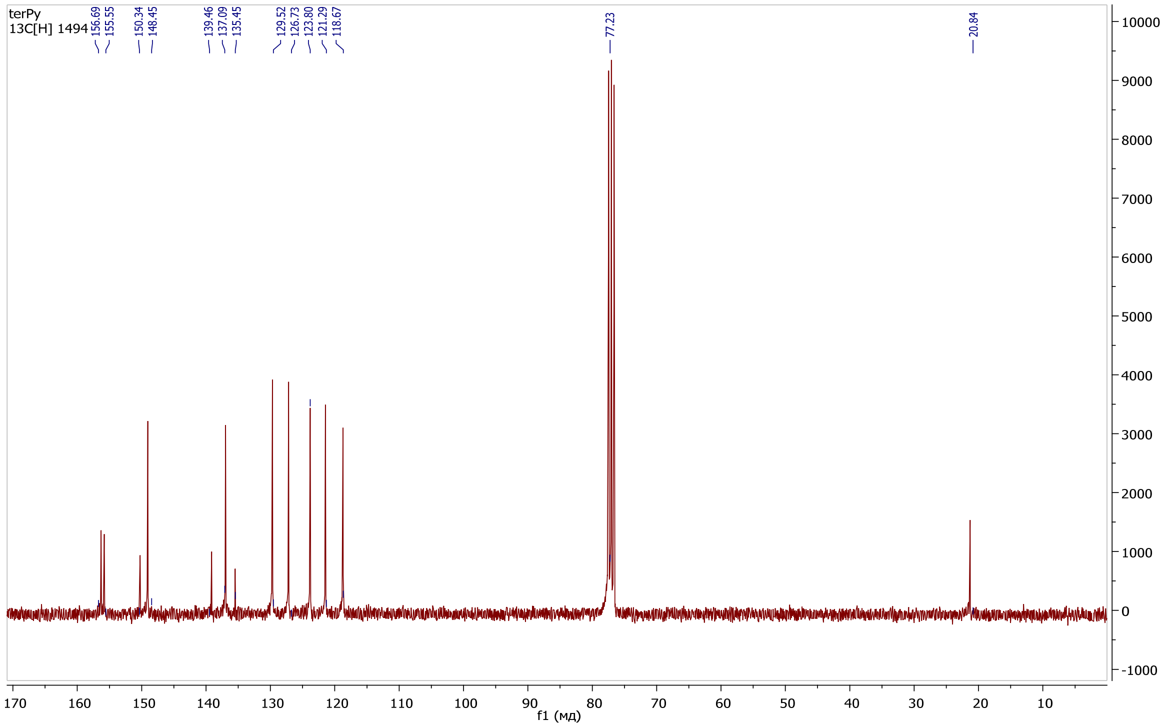

Figure S2. ^{13}C-NMR spectrum ($CDCl_3$) of compound **1**

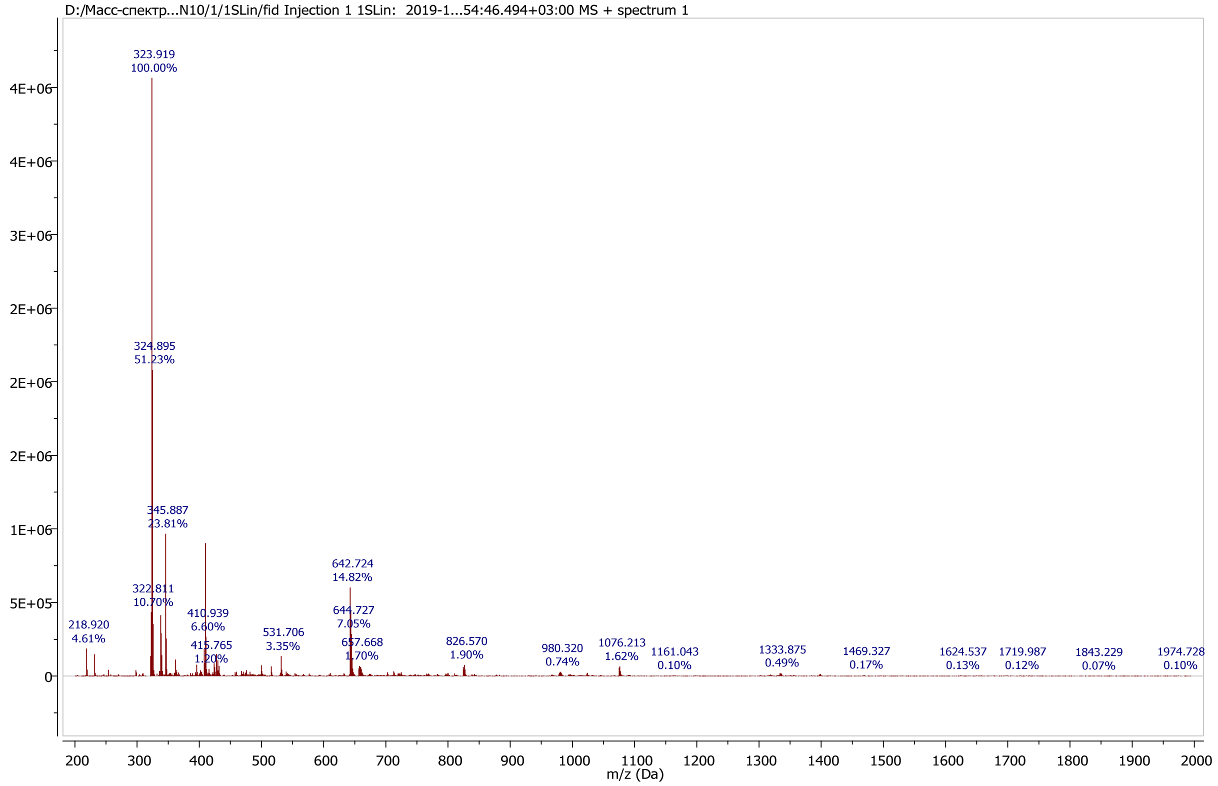

Figure S3. MALDI-TOF mass-spectrum of **1**

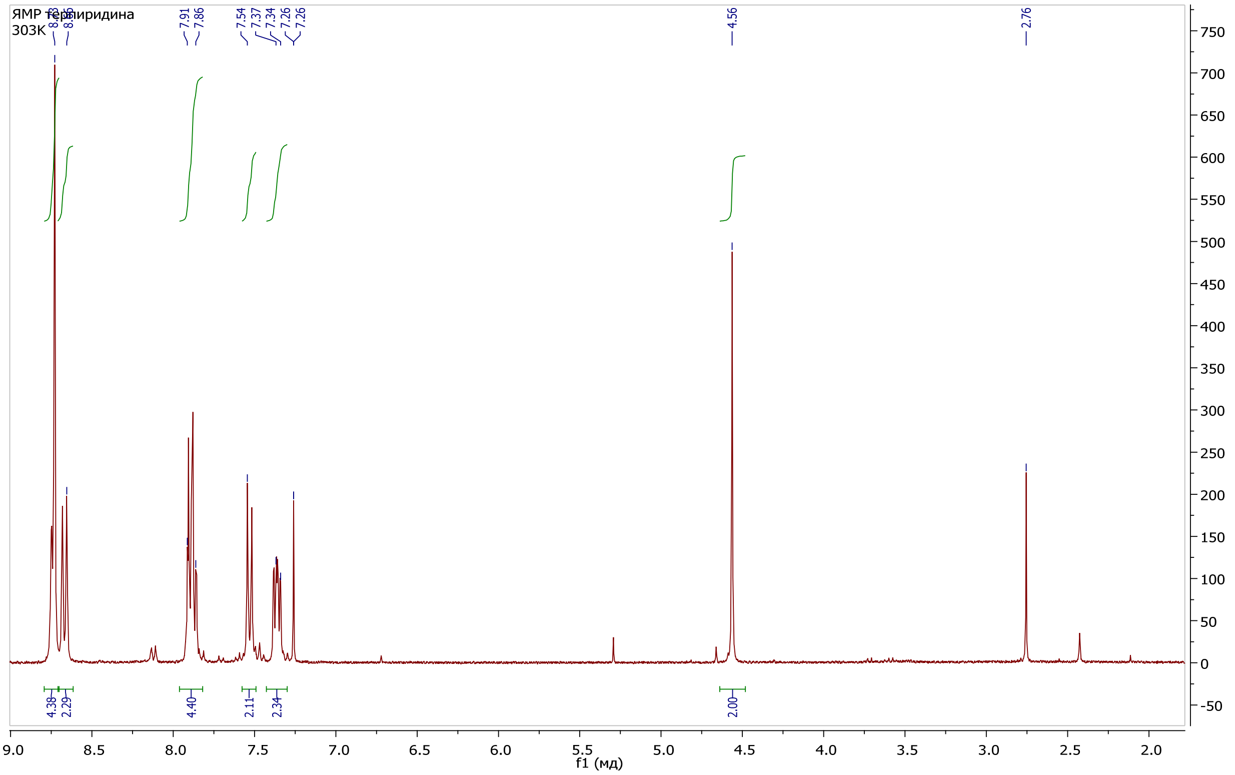

Figure S4.^{1}H-NMR spectrum ($CDCl_3$) of compound **2**

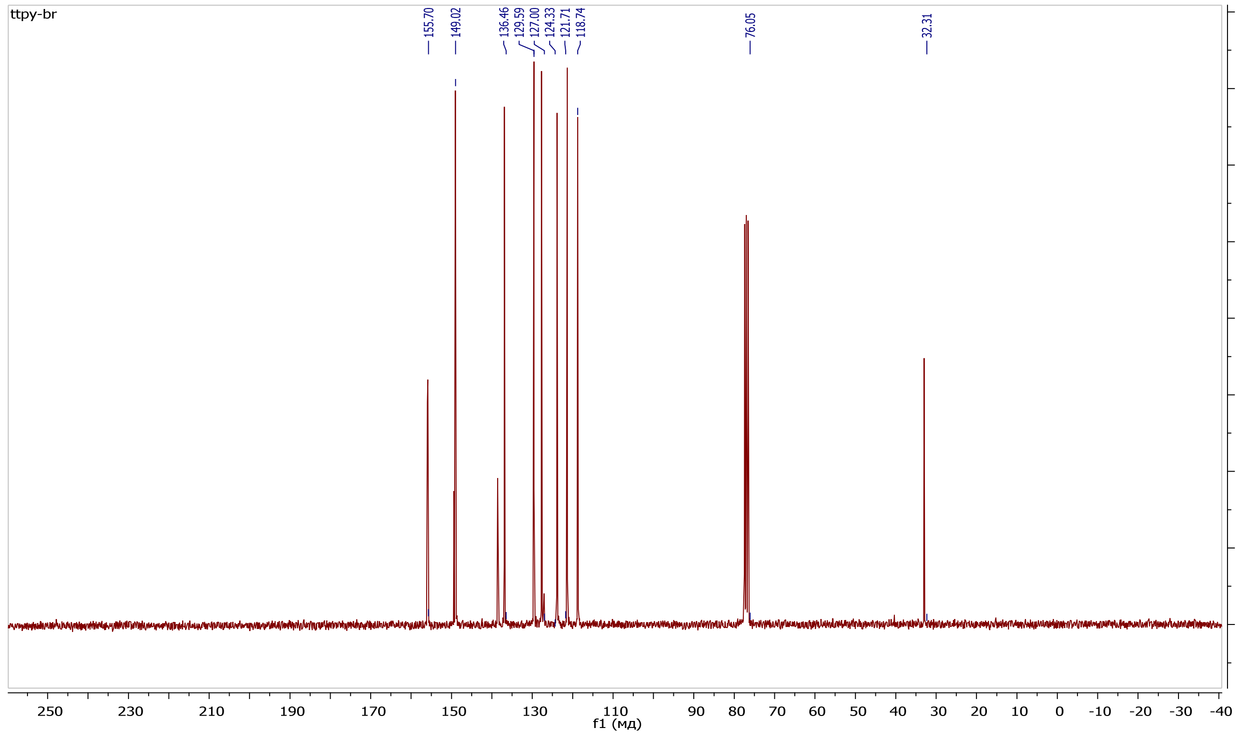

Figure S5.^{13}C-NMR spectrum ($CDCl_3$) of compound **2**

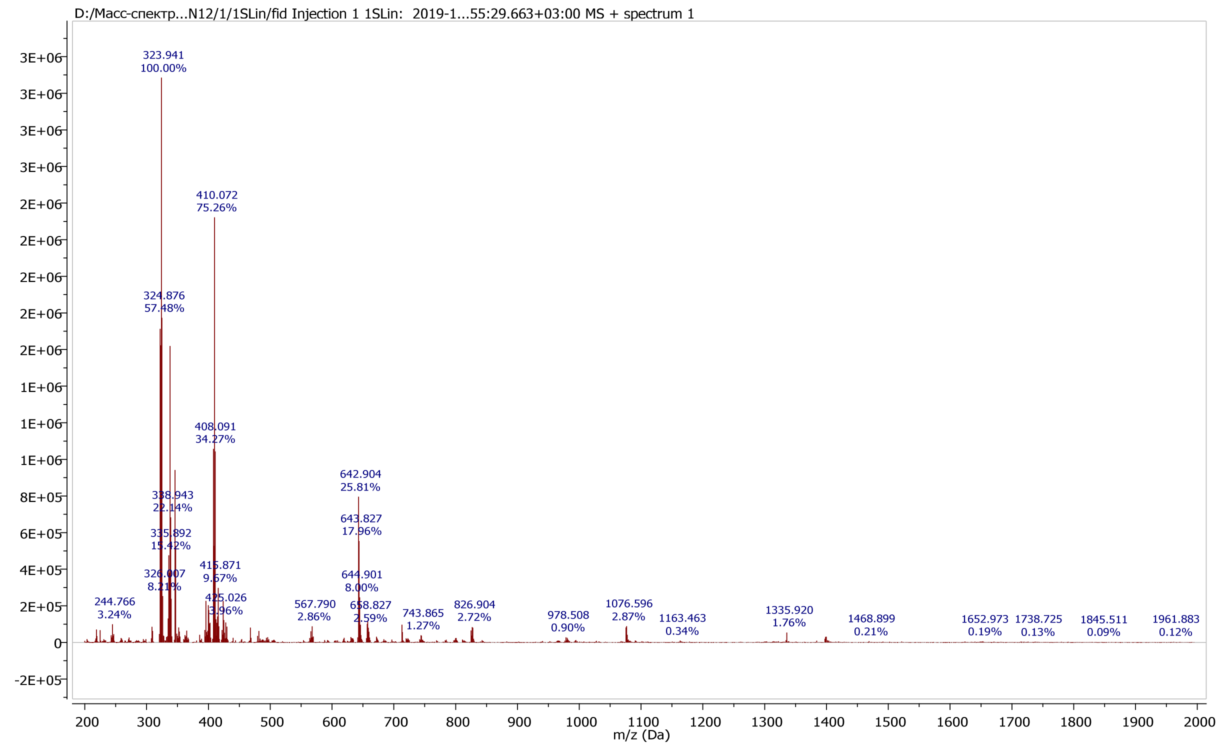

Figure S6. MALDI-TOF mass-spectrum of **2**

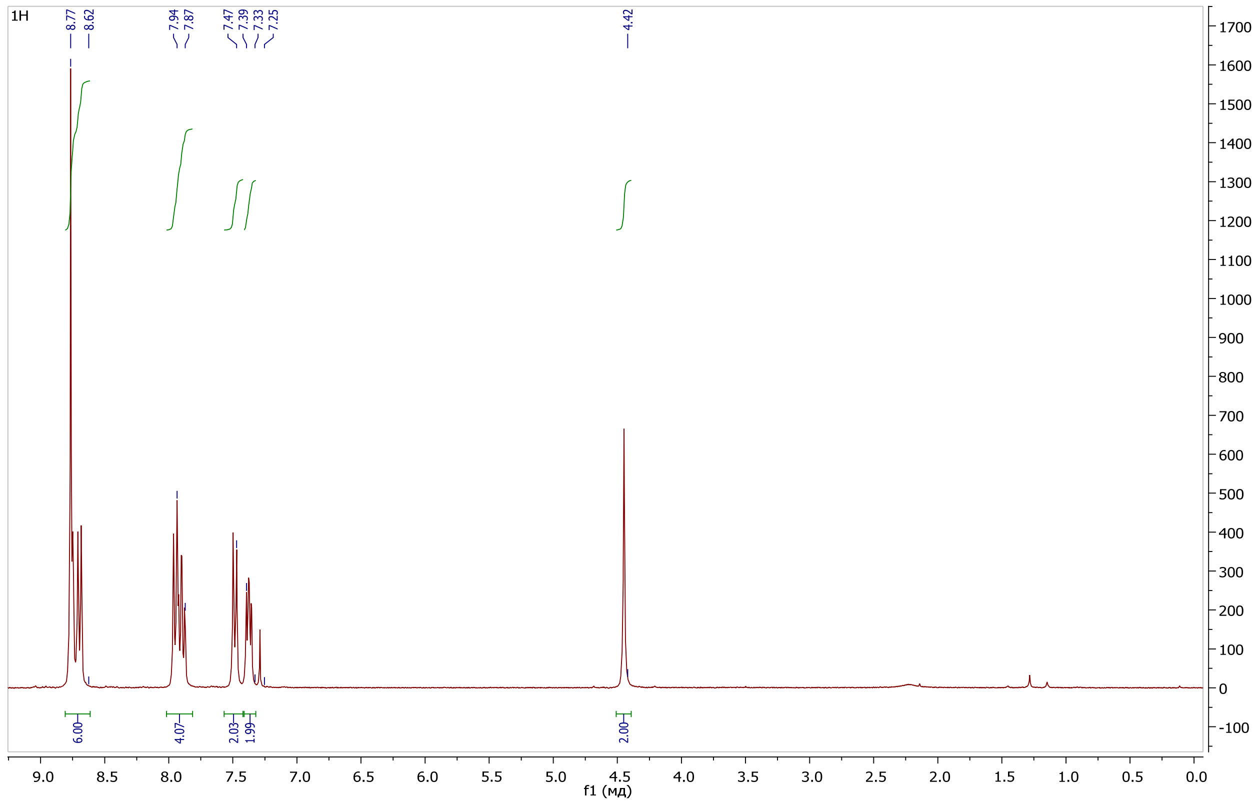

Figure S7. ^{1}H-NMR spectrum ($CDCl_3$) of 4′-(4-azidomethylphenyl)-2,2′:6′,2″-terpyridine

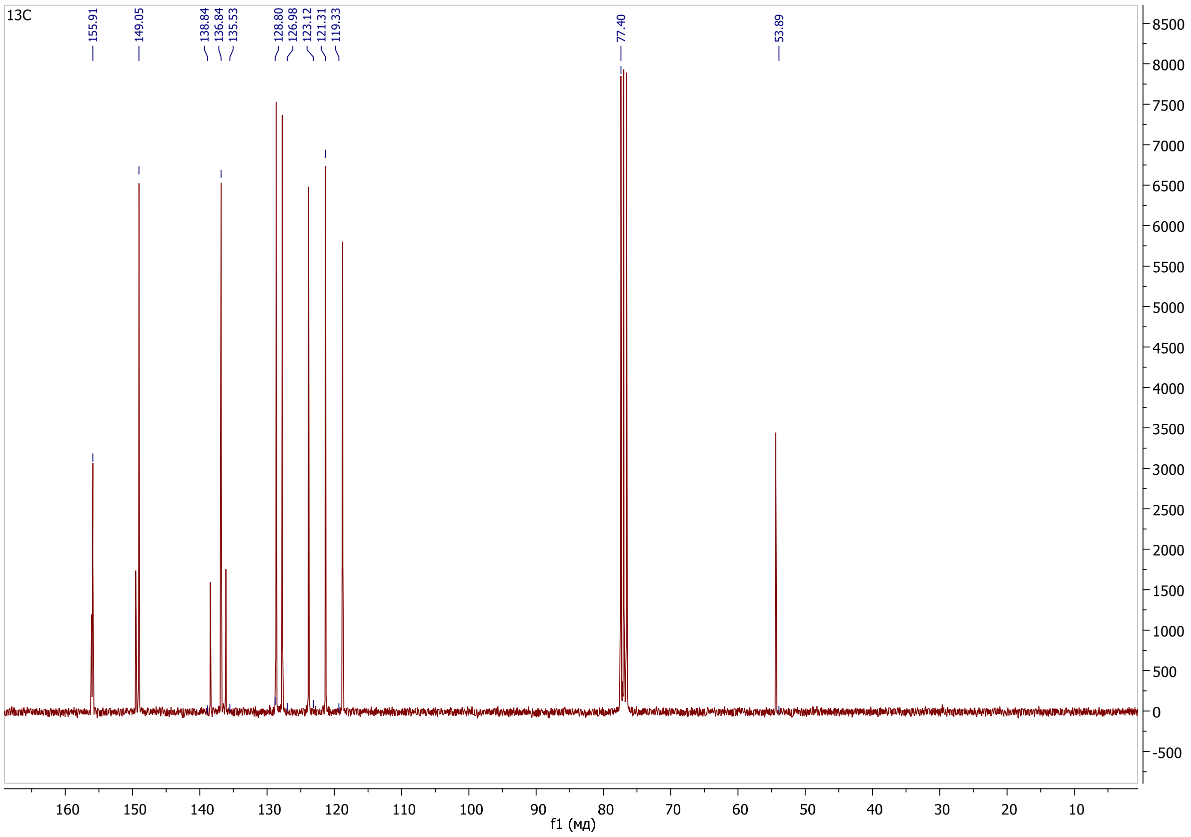

Figure S8. ^{13}C-NMR spectrum ($CDCl_3$) of 4′-(4-azidomethylphenyl)-2,2′:6′,2″-terpyridine

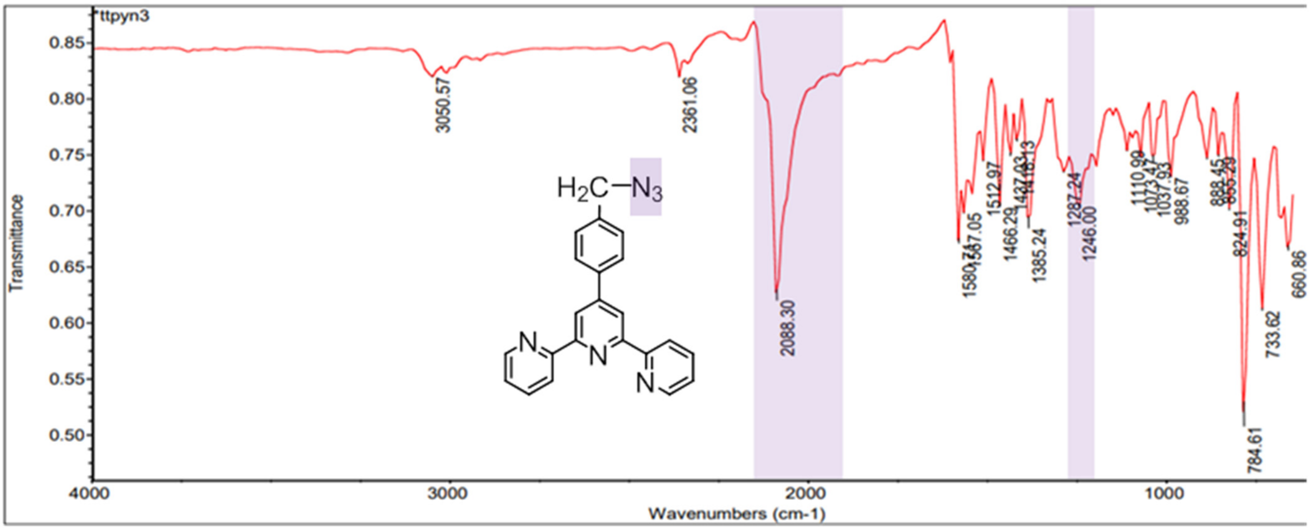

Figure S9. FTIR – spectrum of 4′-(4-azidomethylphenyl)-2,2′:6′,2″-terpyridine

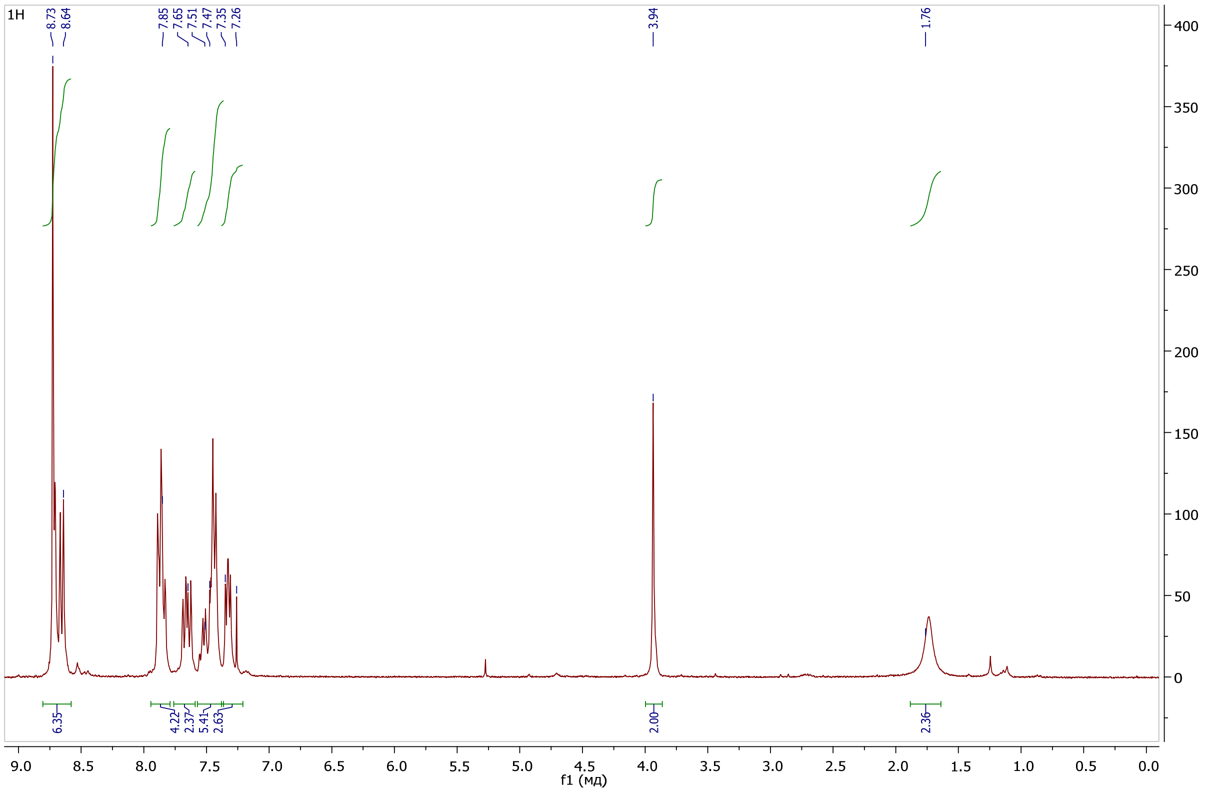

Figure S10. ^{1}H-NMR spectrum ($CDCl_3$) of compound **3**

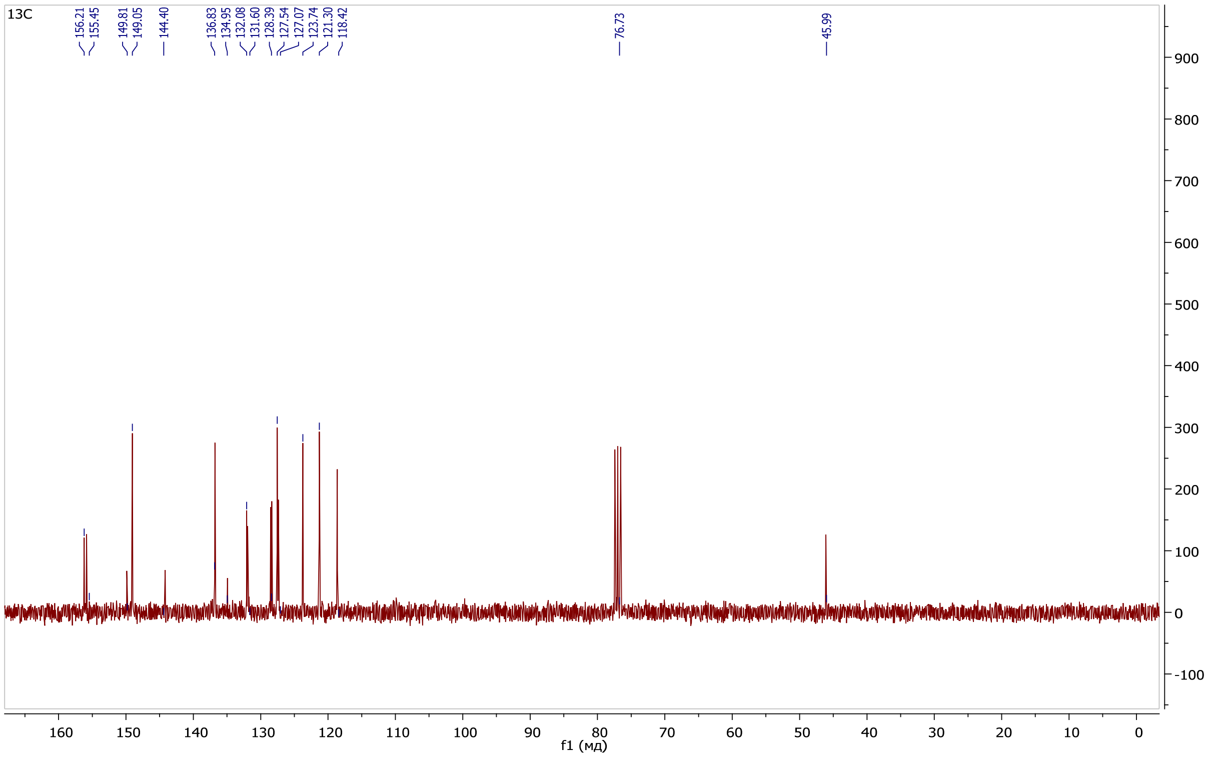

Figure S11.^{13}C-NMR spectrum ($CDCl_3$) of compound **3**

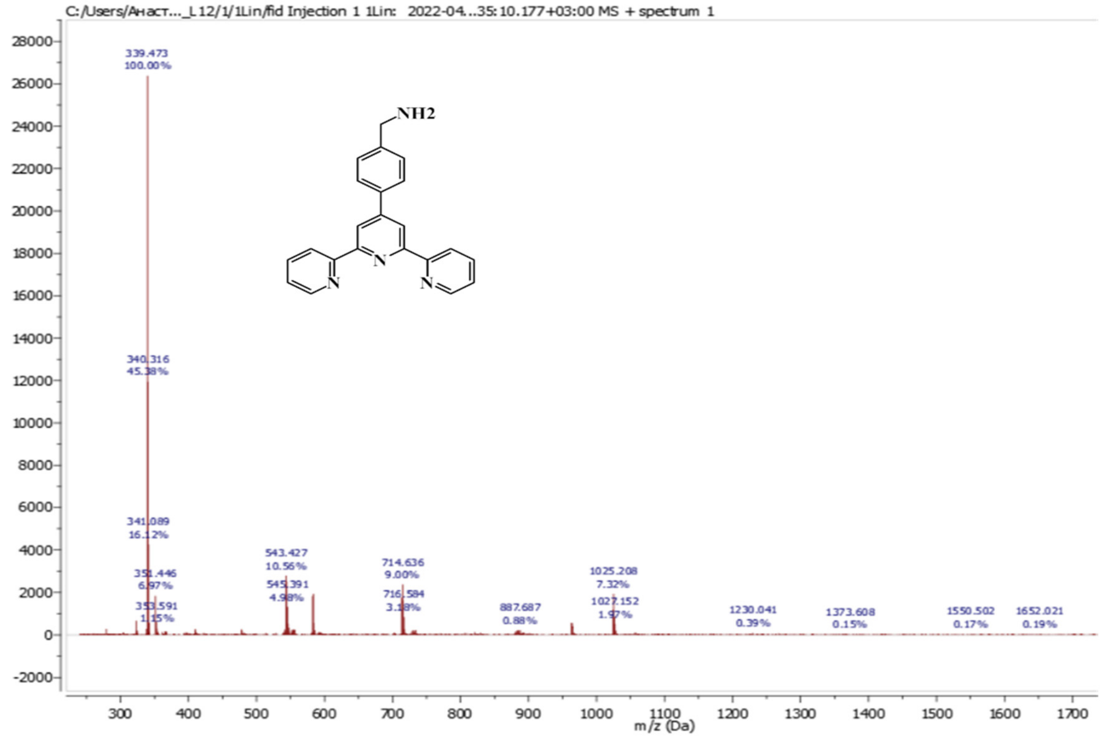

Figure S12. MALDI – TOF Mass-spectrum of compound **3**

^{1}H NMR, ^{13}C NMR, mass-spectra data for porphyrins 4-11 are illustrated below (see Figures S13-35).

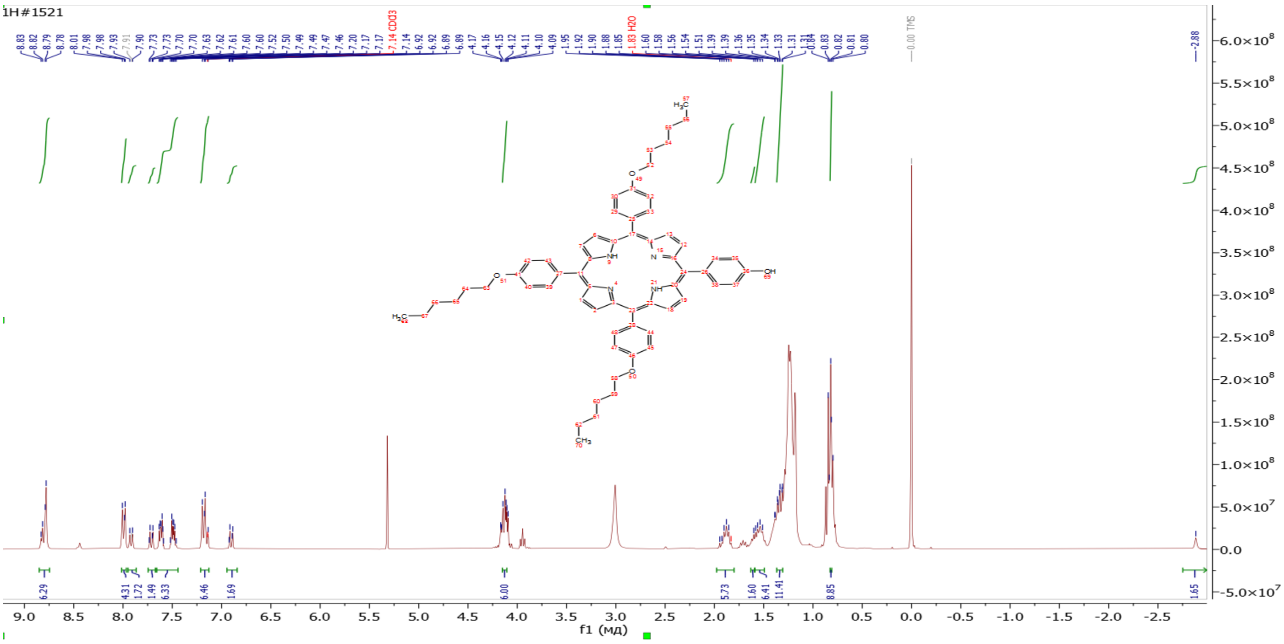

Figure S13. ^{1}H-NMR spectrum ($CDCl_3$) of compound **4**

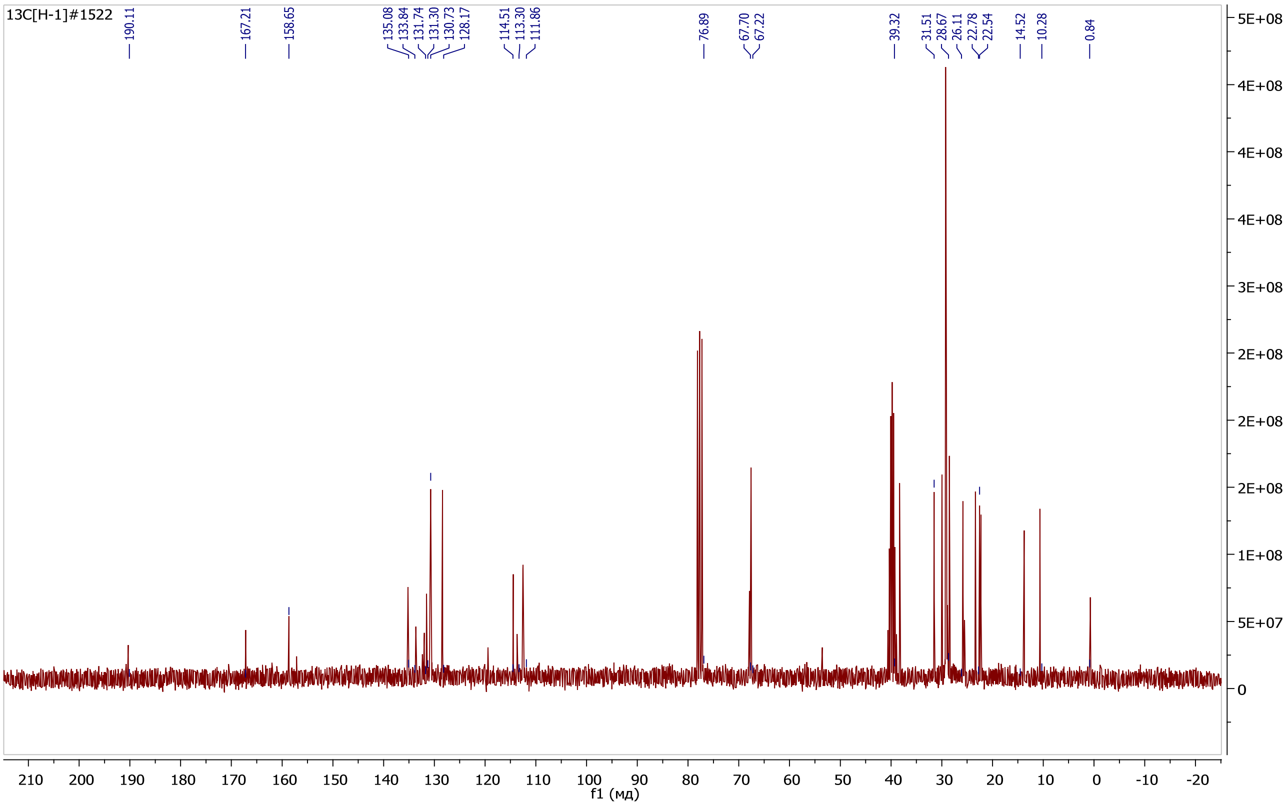

Figure S14.^{13}C-NMR spectrum ($CDCl_3$) of compound **4**

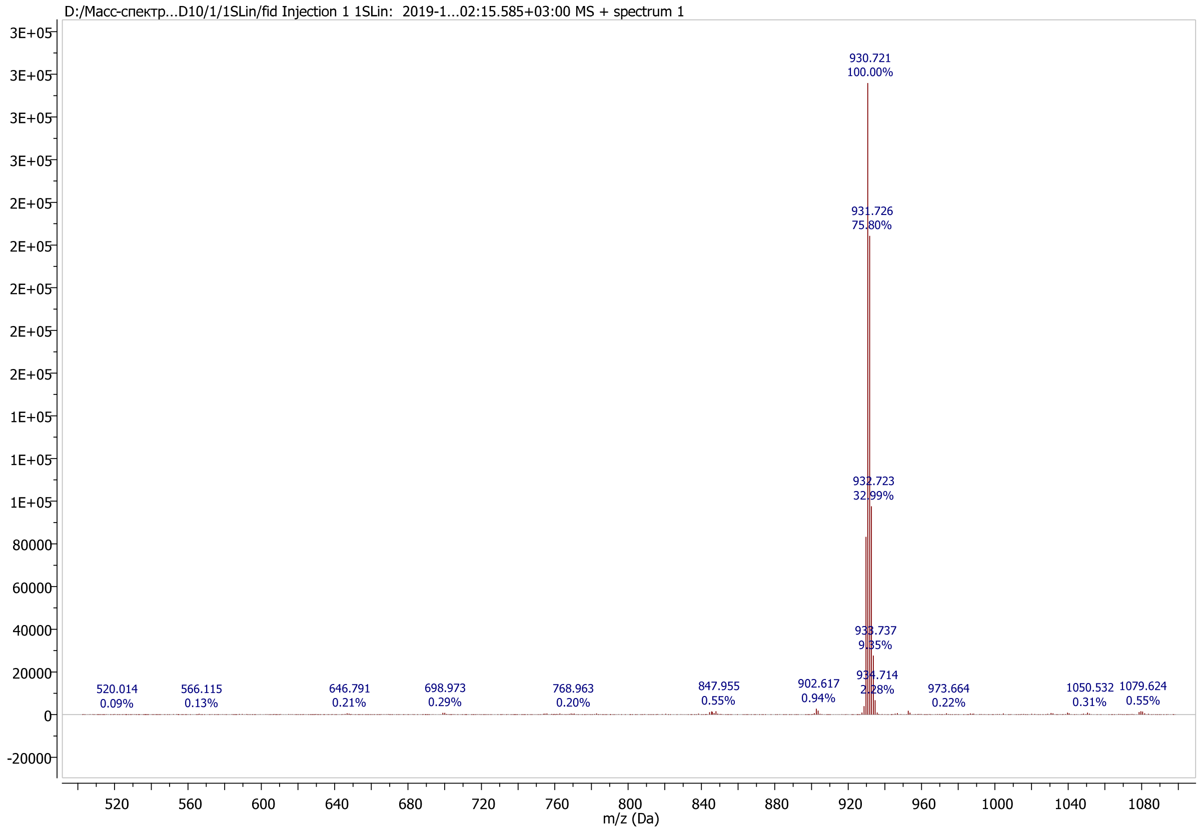

Figure S15. MALDI – TOF Mass-spectrum of compound **4**

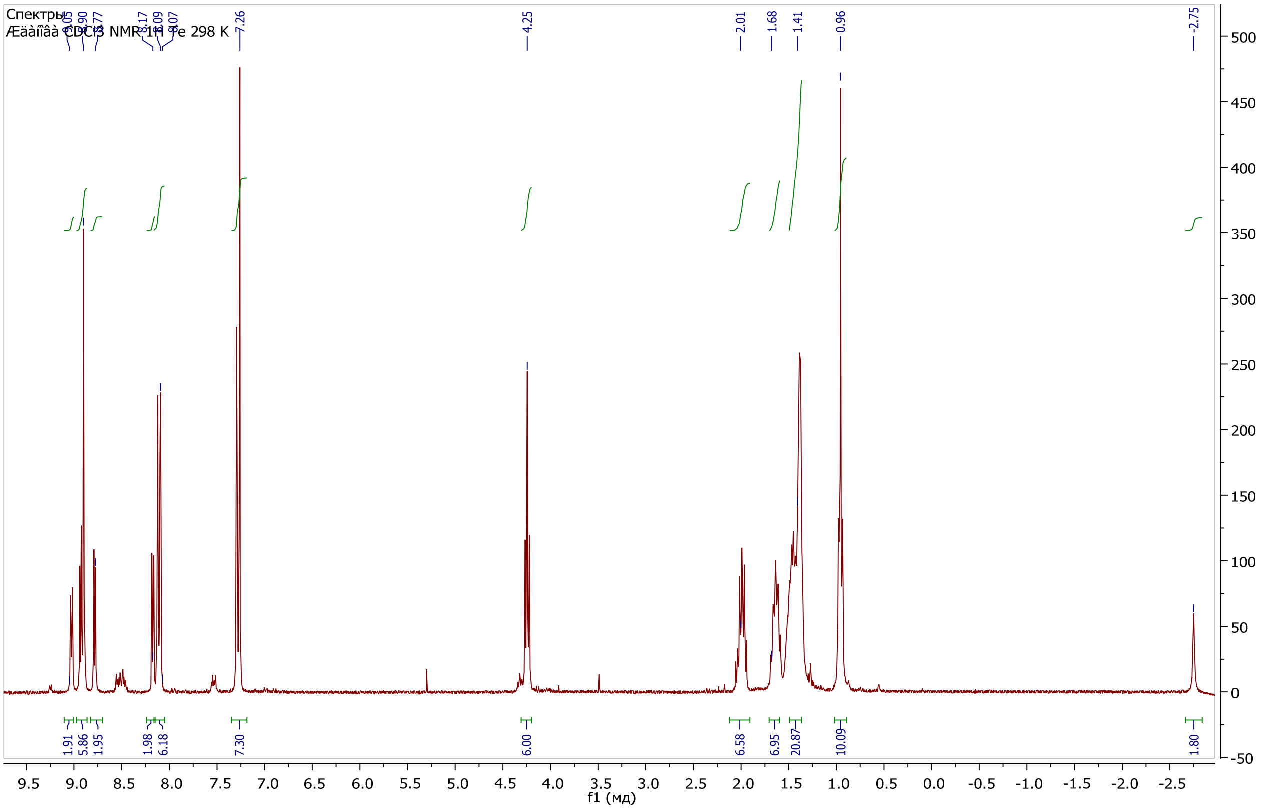

Figure S16. ^{1}H-NMR spectrum ($CDCl_3$) of compound **5**

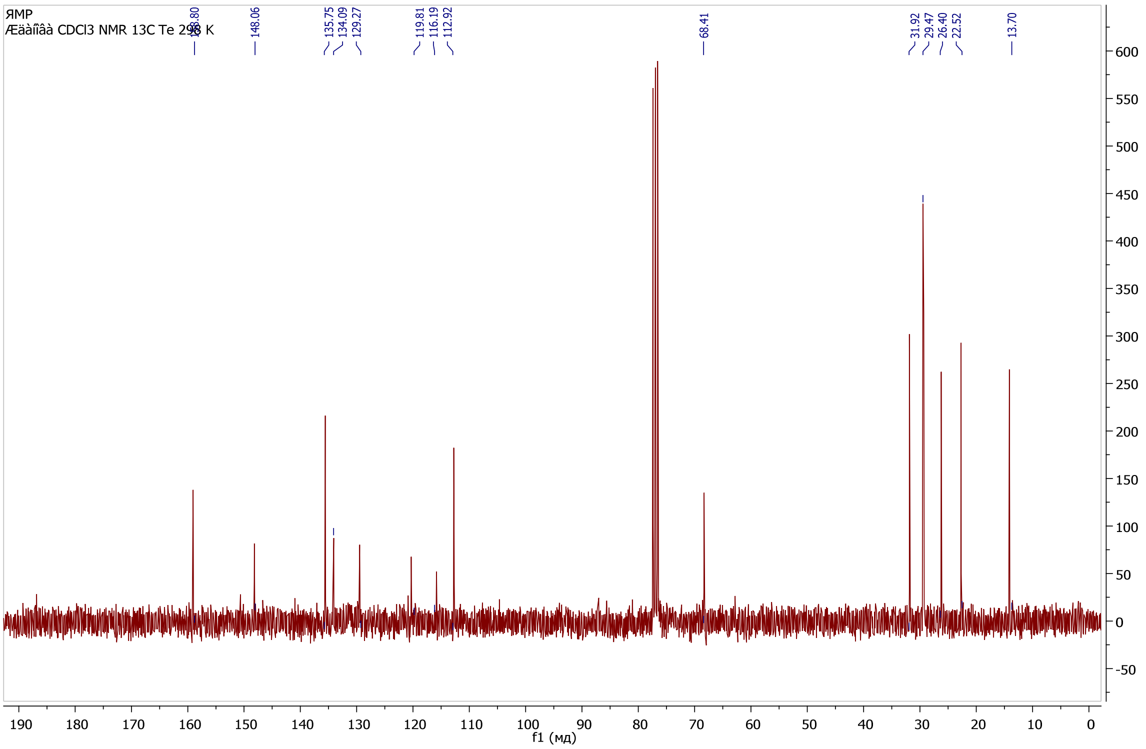

Figure S17.^{13}C-NMR spectrum ($CDCl_3$) of compound **5**

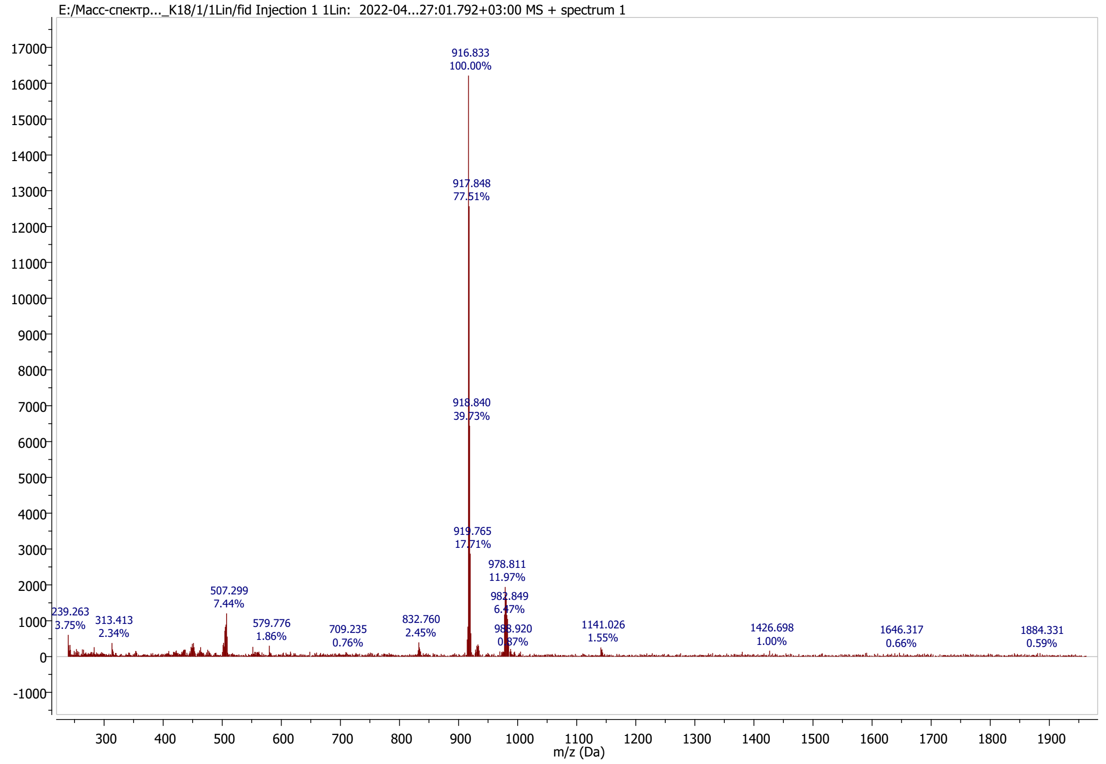

Figure S18. MALDI – TOF Mass-spectrum of compound **5**

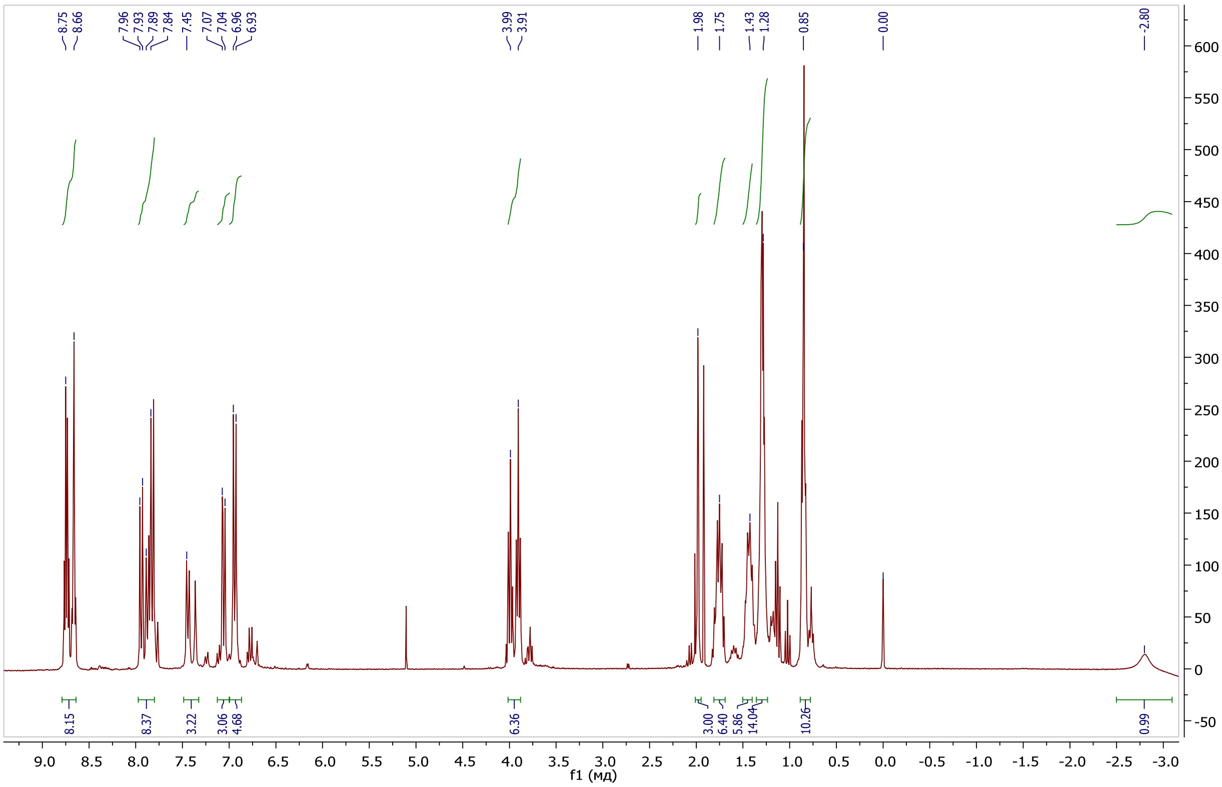

Figure S19. ^{1}H-NMR spectrum ($CDCl_3$) of 5-(4-Acetamidophenyl)-10,15,20-tris(4-hexyloxyphenyl)porphyrin

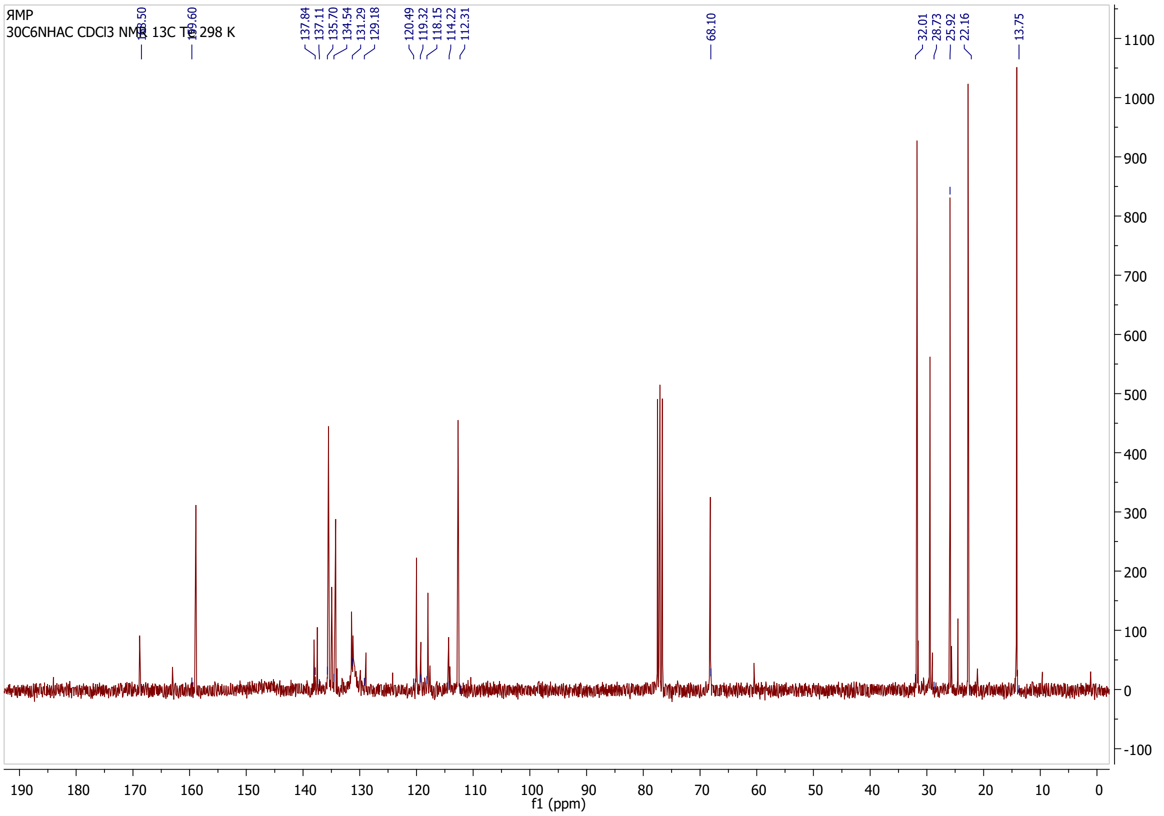

Figure S20. ^{13}C-NMR spectrum ($CDCl_3$) of 5-(4-Acetamidophenyl)-10,15,20-tri(4-hexyloxyphenyl)porphyrin

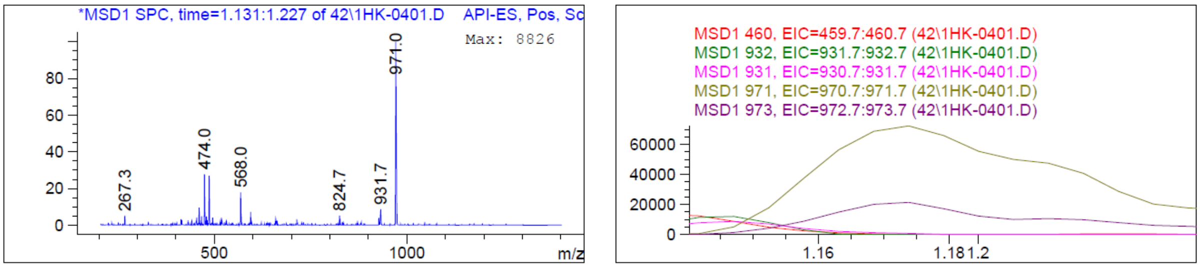

Figure S21. APCI mass spectrum of 5-(4-Acetamidophenyl)-10,15,20-tri(4-hexyloxyphenyl)porphyrin

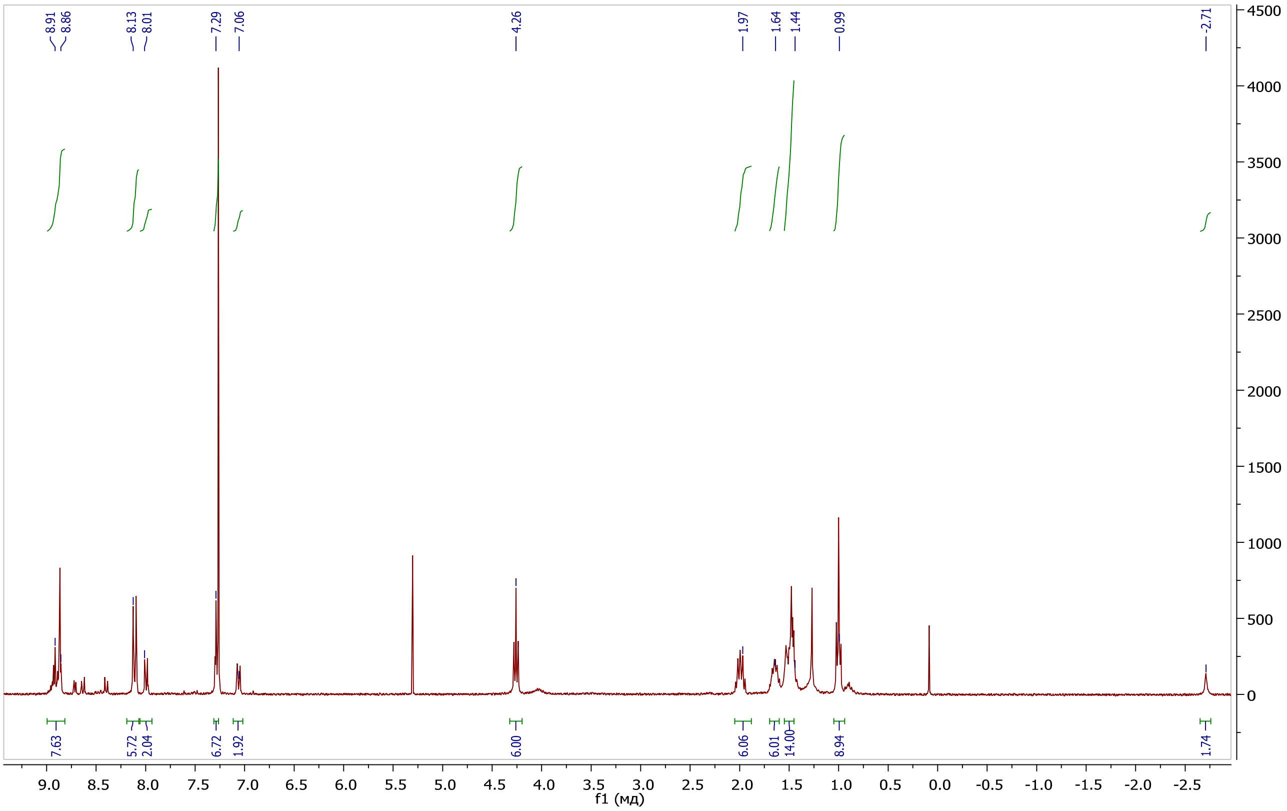

Figure S22. ^{1}H-NMR spectrum ($CDCl_3$) of compound **6**

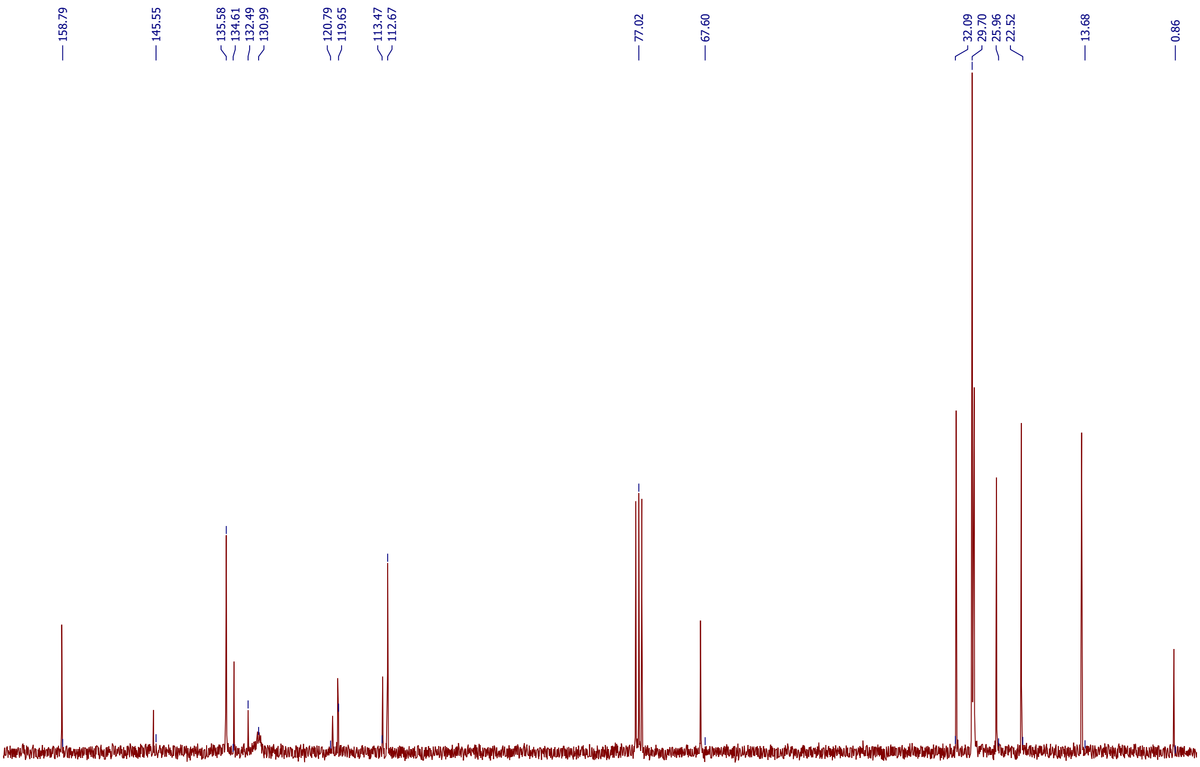

Figure S23. ^{13}C-NMR spectrum ($CDCl_3$) of compound **6**

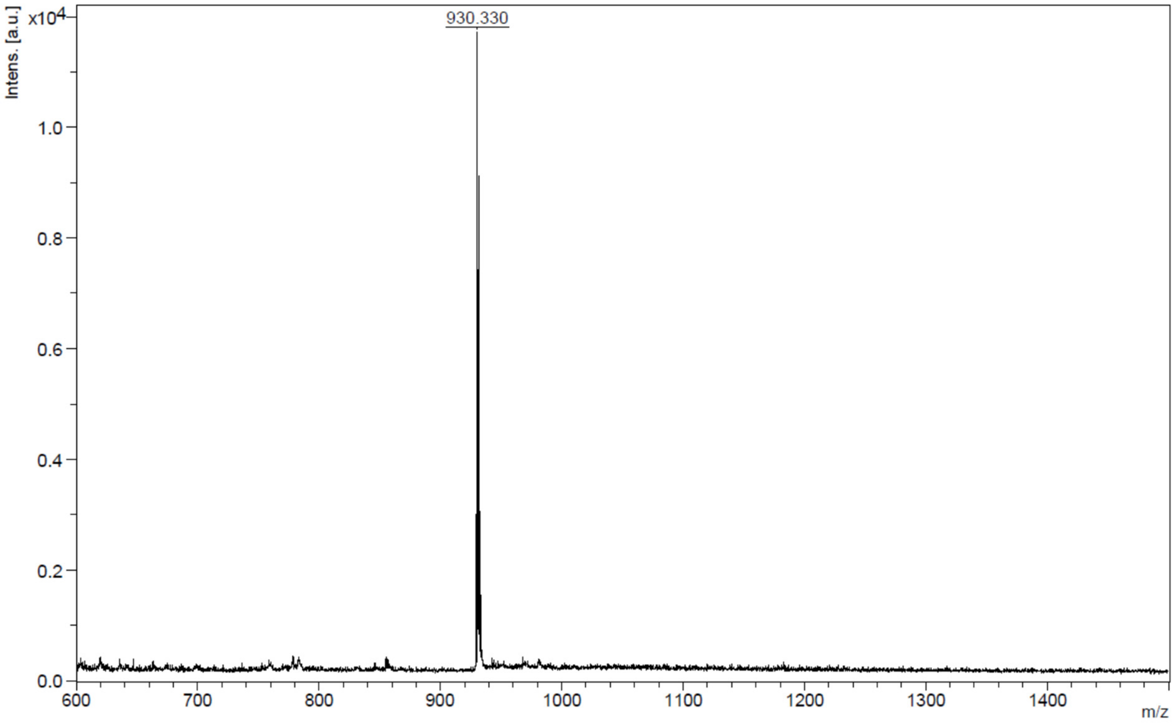

Figure S24. MALDI – TOF Mass-spectrum of compound **6**

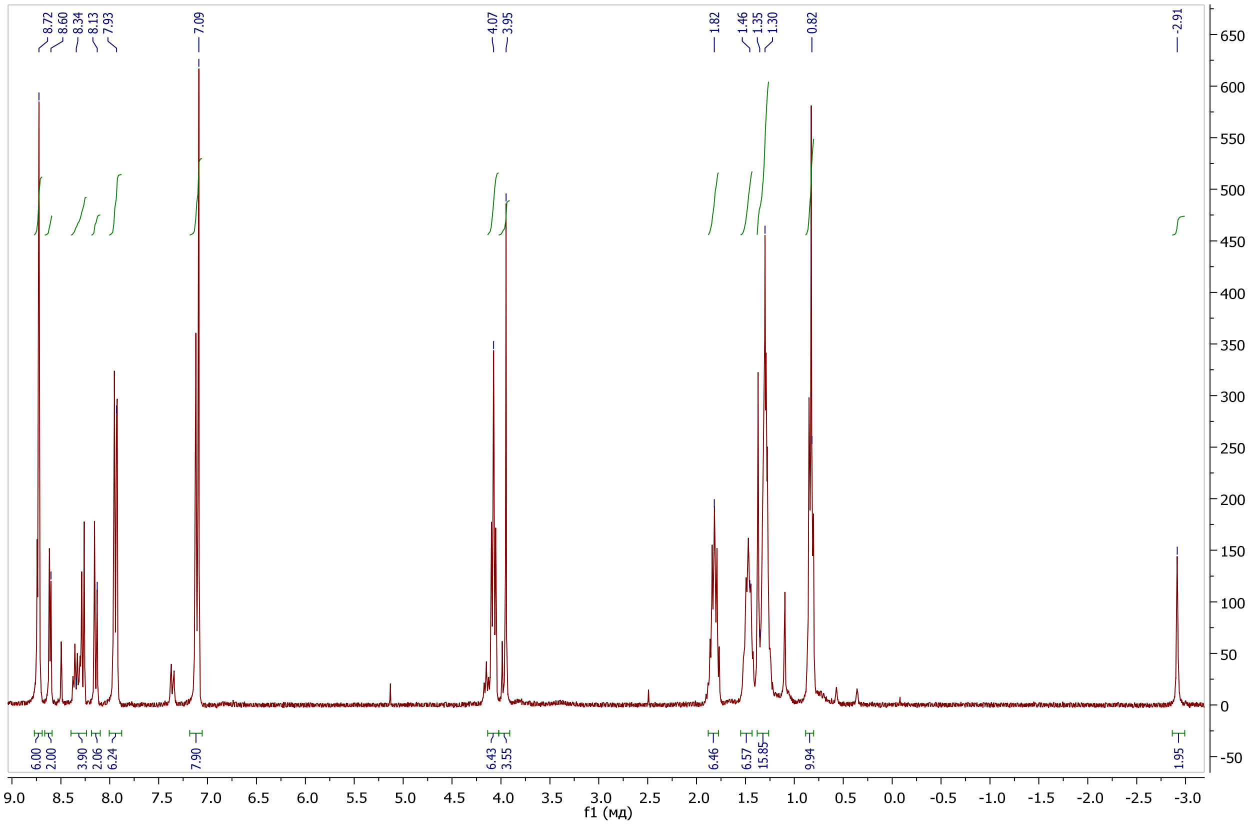

Figure S25. ^{1}H-NMR spectrum ($CDCl_3$) of compound **7**

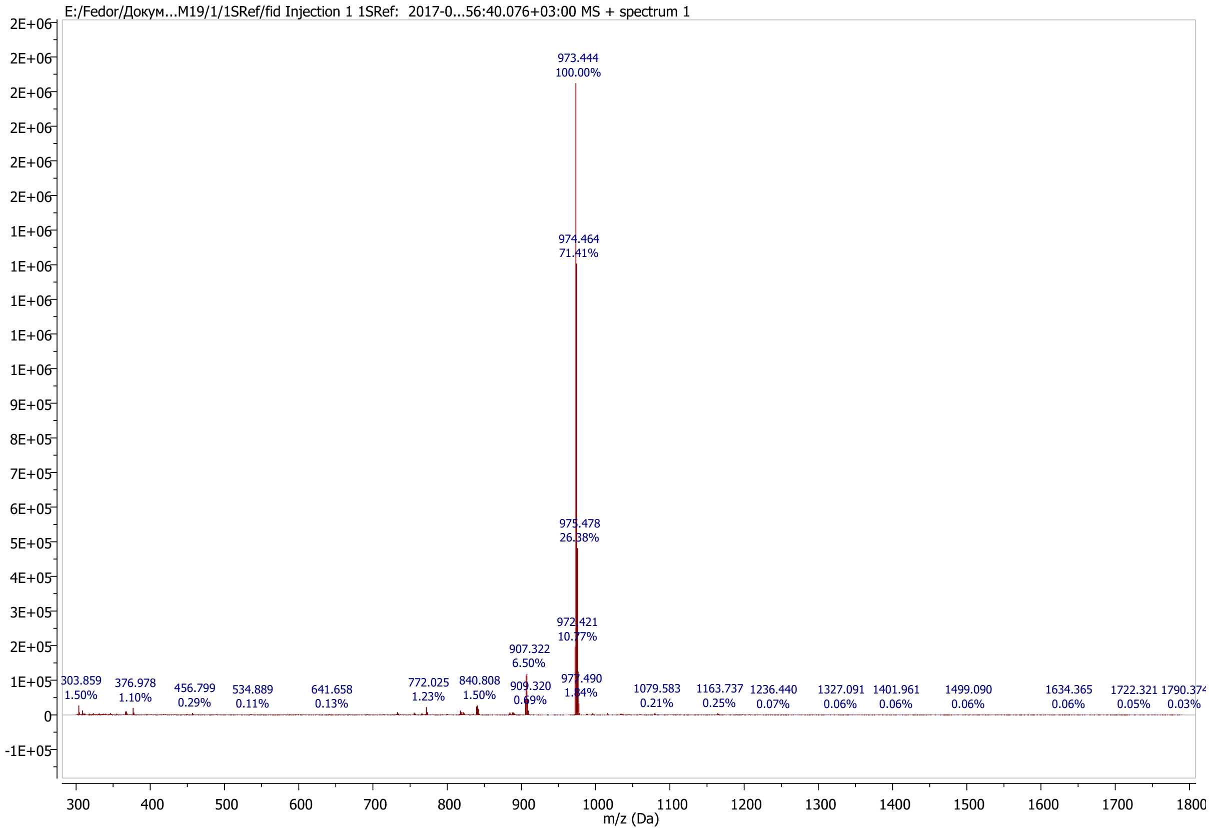

Figure S26. MALDI – TOF Mass-spectrum of compound **7**

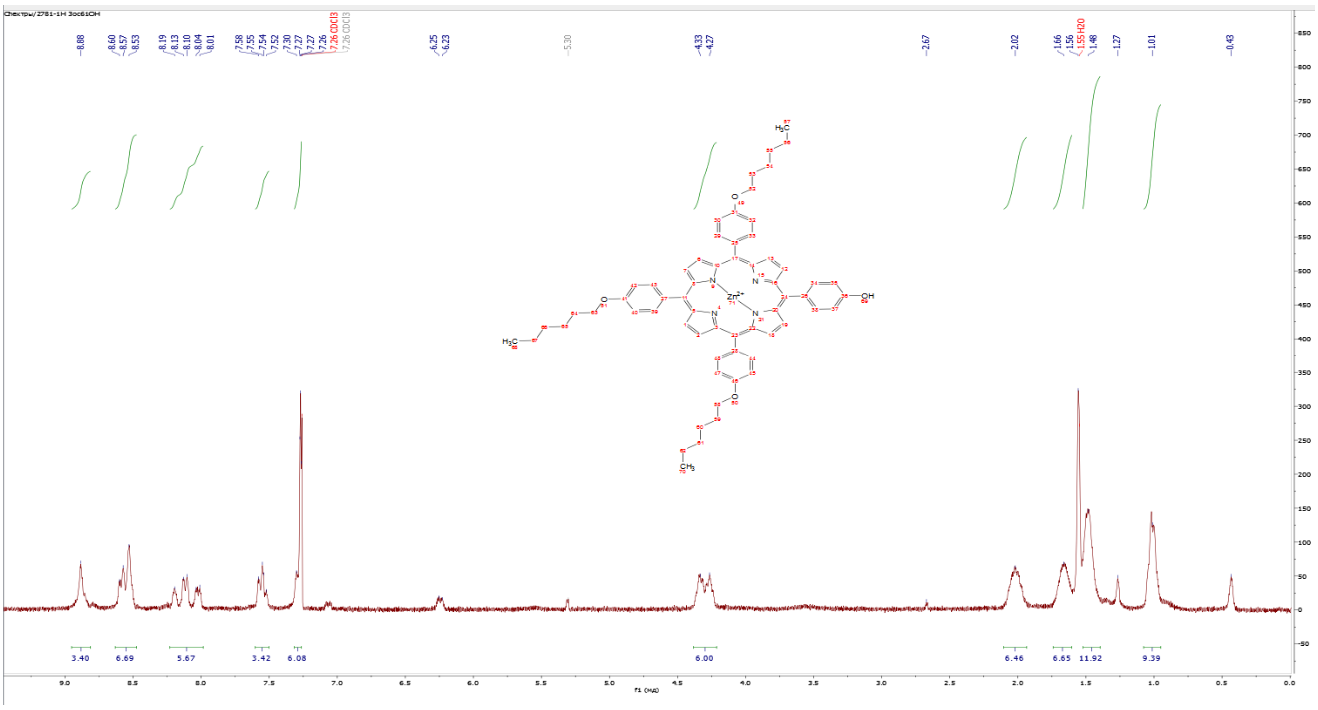

Figure S27. ^{1}H-NMR spectrum ($CDCl_3$) of compound **8**

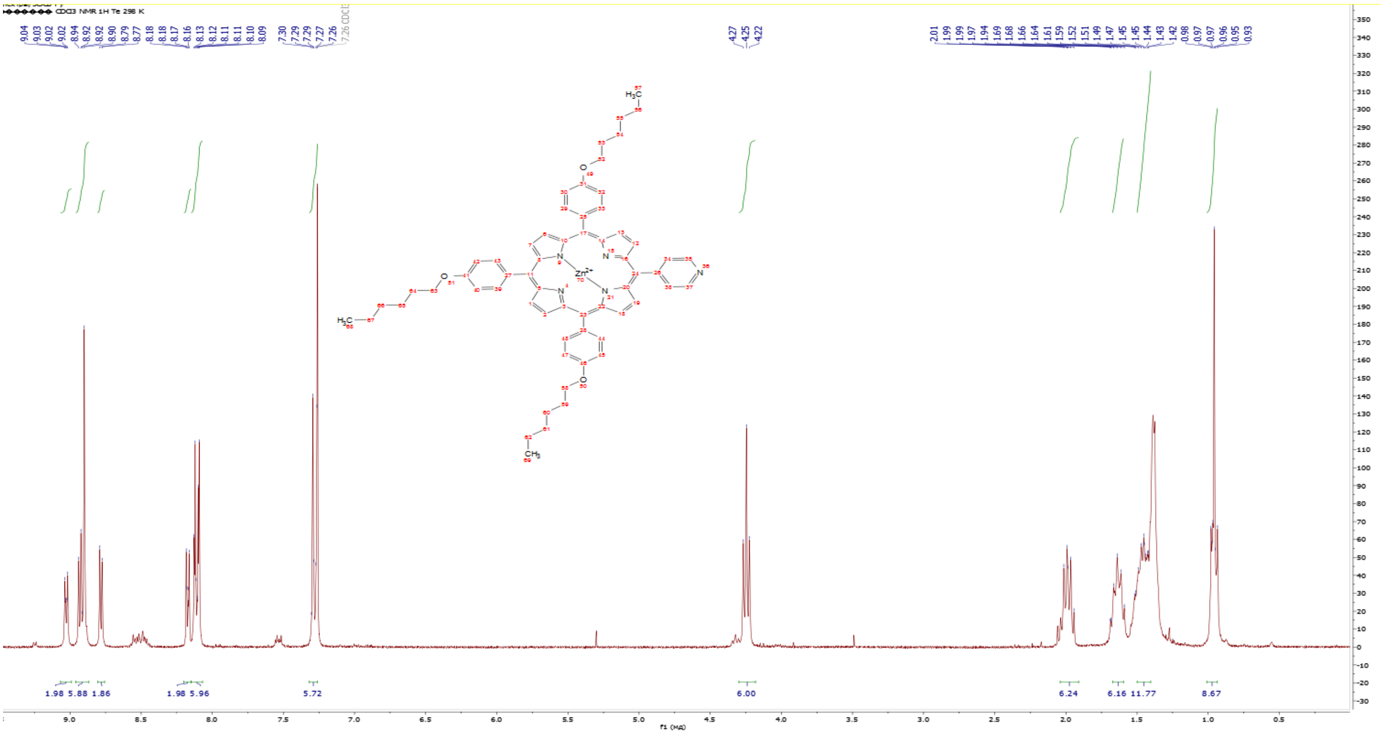

Figure S28. ^{1}H-NMR spectrum ($CDCl_3$) of compound **9**

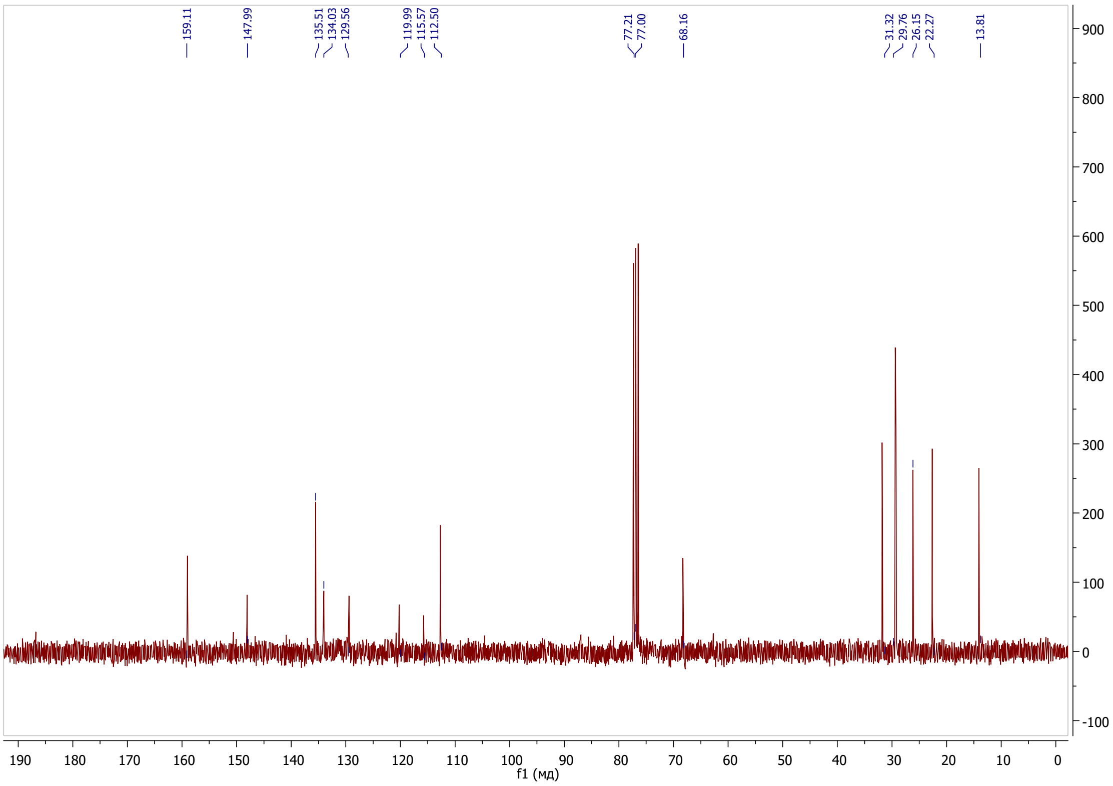

Figure S29. ^{13}C-NMR spectrum ($CDCl_3$) of compound **9**

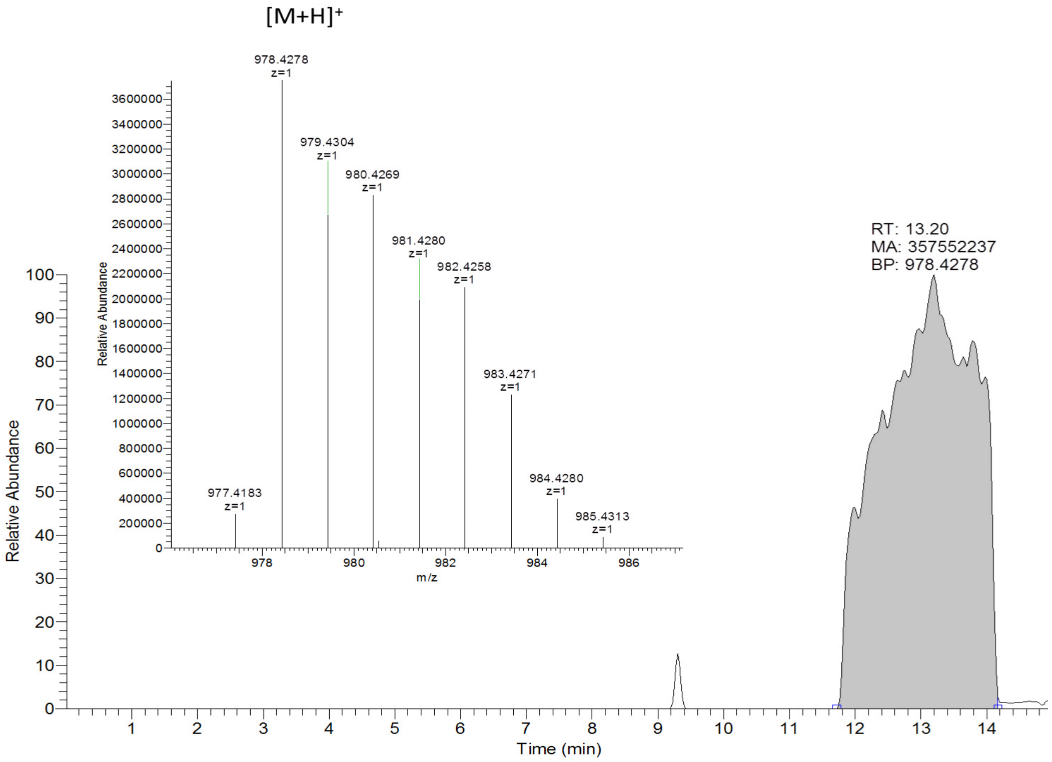

Figure S30. HRMS Mass-spectrum of compound **9**.

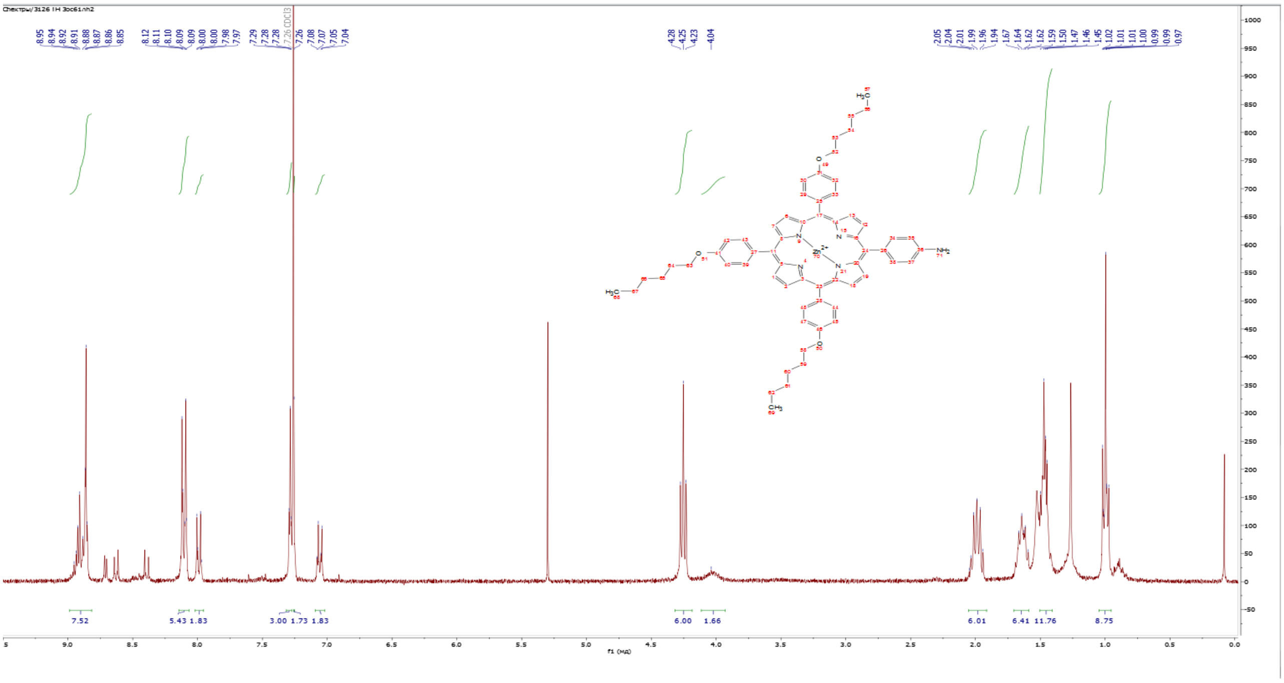

Figure S31. ^{1}H-NMR spectrum ($CDCl_3$) of compound **10**

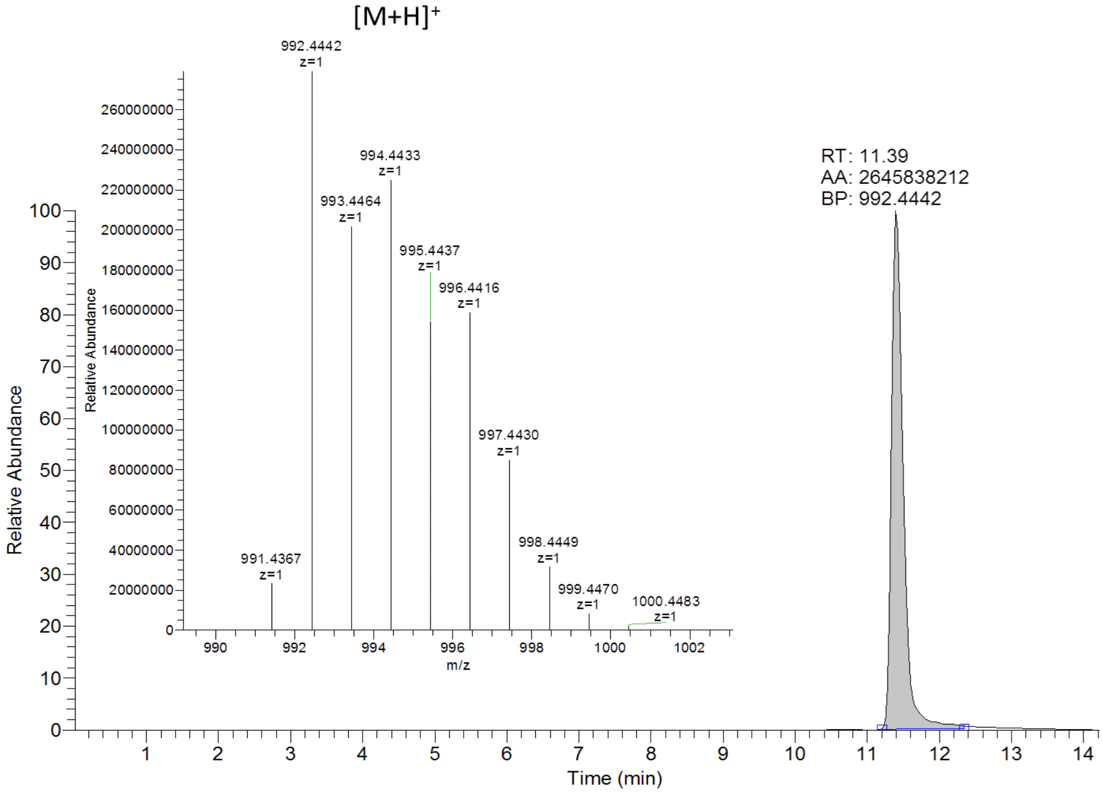

Figure S32. MALDI – TOF Mass-spectrum of compound **10**.

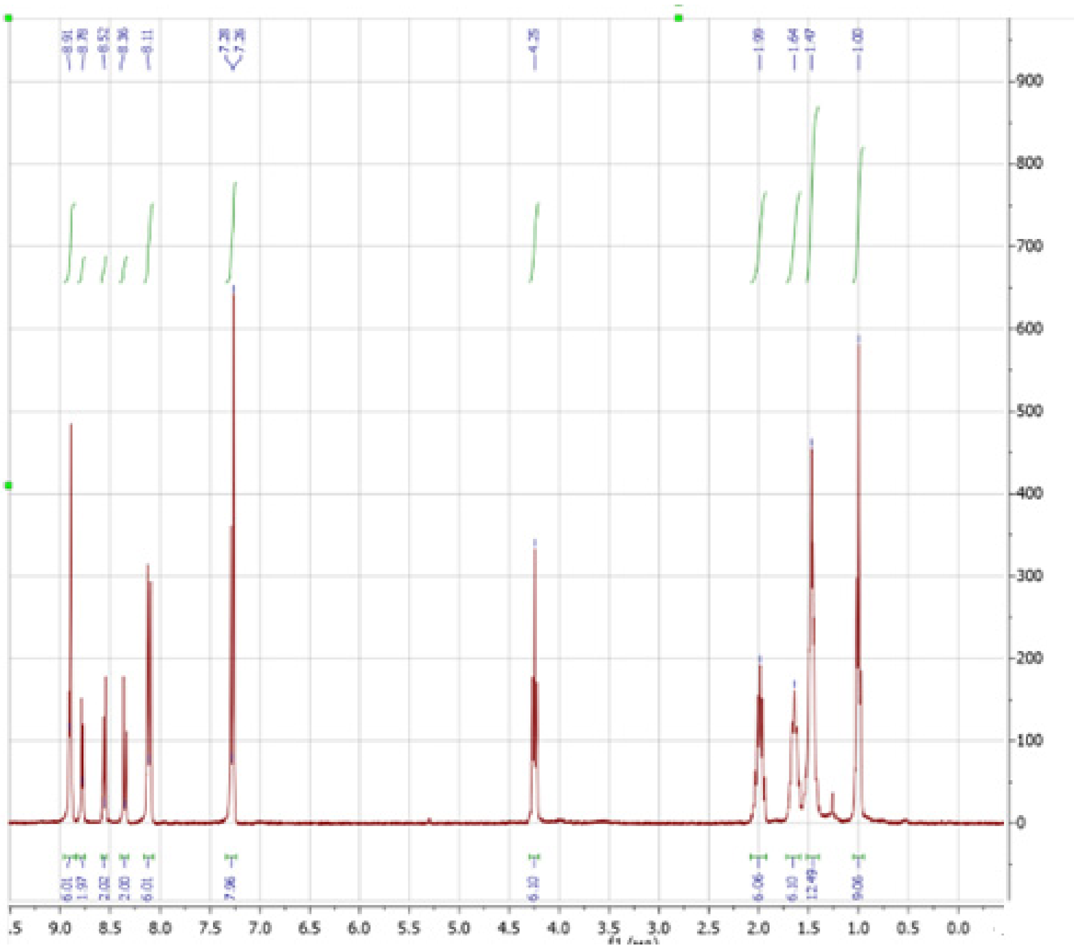

Figure S33. ^{1}H-NMR spectrum ($CDCl_3$) of compound 11

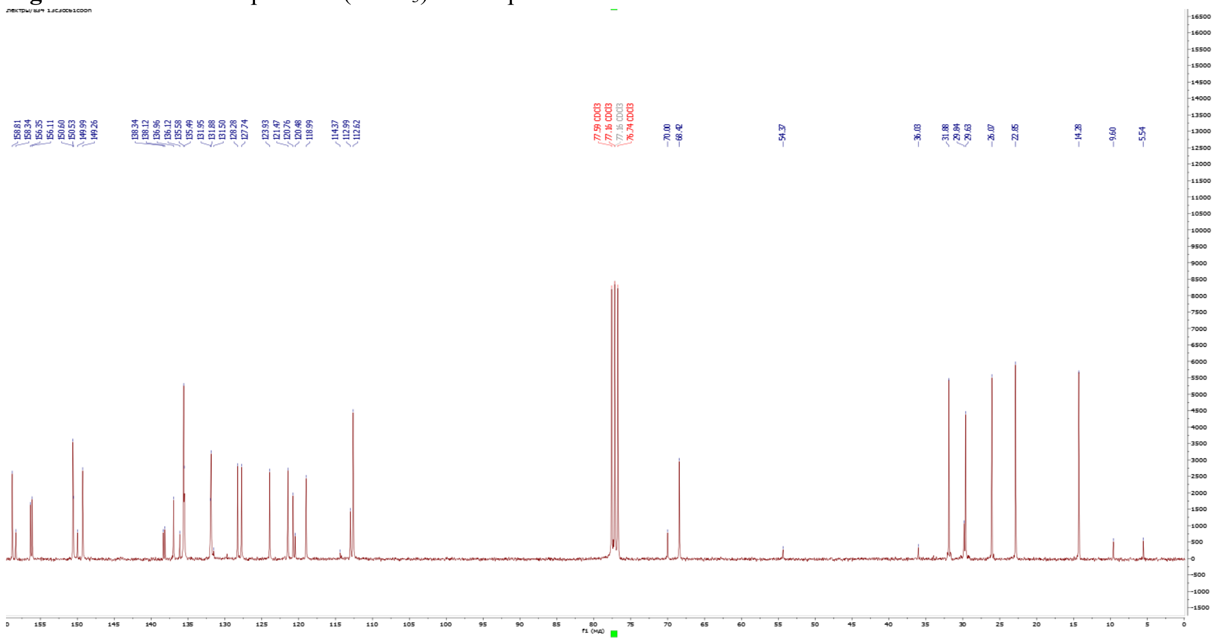

Figure S34. ^{13}C-NMR spectrum ($CDCl_3$) of compound **11**

Acquisition Parameter

Source Type	ESI	Ion Polarity	Positive	Set Nebulizer	0.4 Bar
Focus	Not active			Set Dry Heater	180 °C
Scan Begin	50 m/z	Set Capillary	4500 V	Set Dry Gas	4.0 l/min
Scan End	3000 m/z	Set End Plate Offset	-500 V	Set Divert Valve	Waste

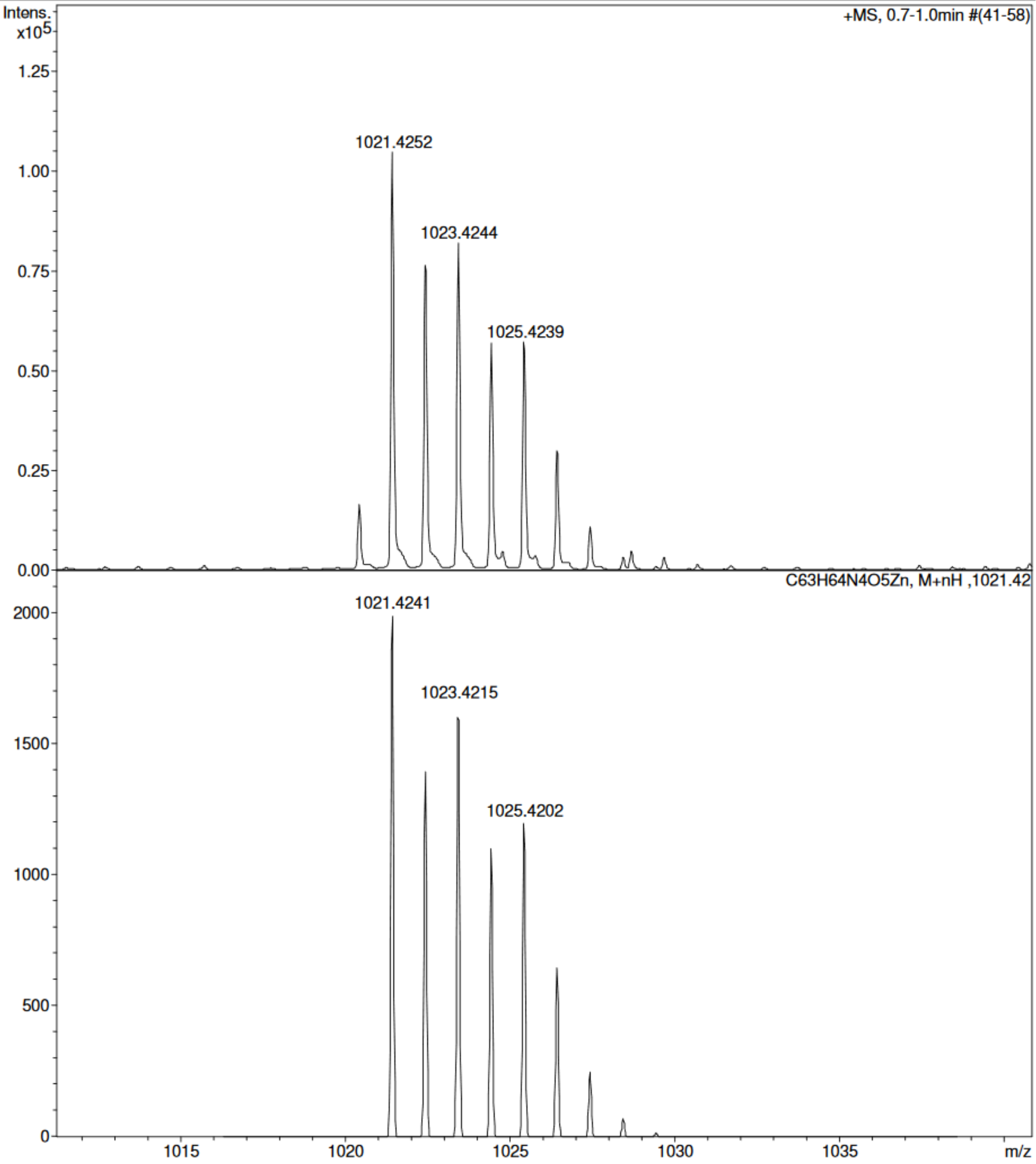

Figure S35. Experimental (upper) and predicted (lower) HRMS spectra of compound **11**.

^{1}H NMR, ^{13}C NMR, mass-spectra data for conjugates 12-15 are illustrated below (see Figures S36-S48).

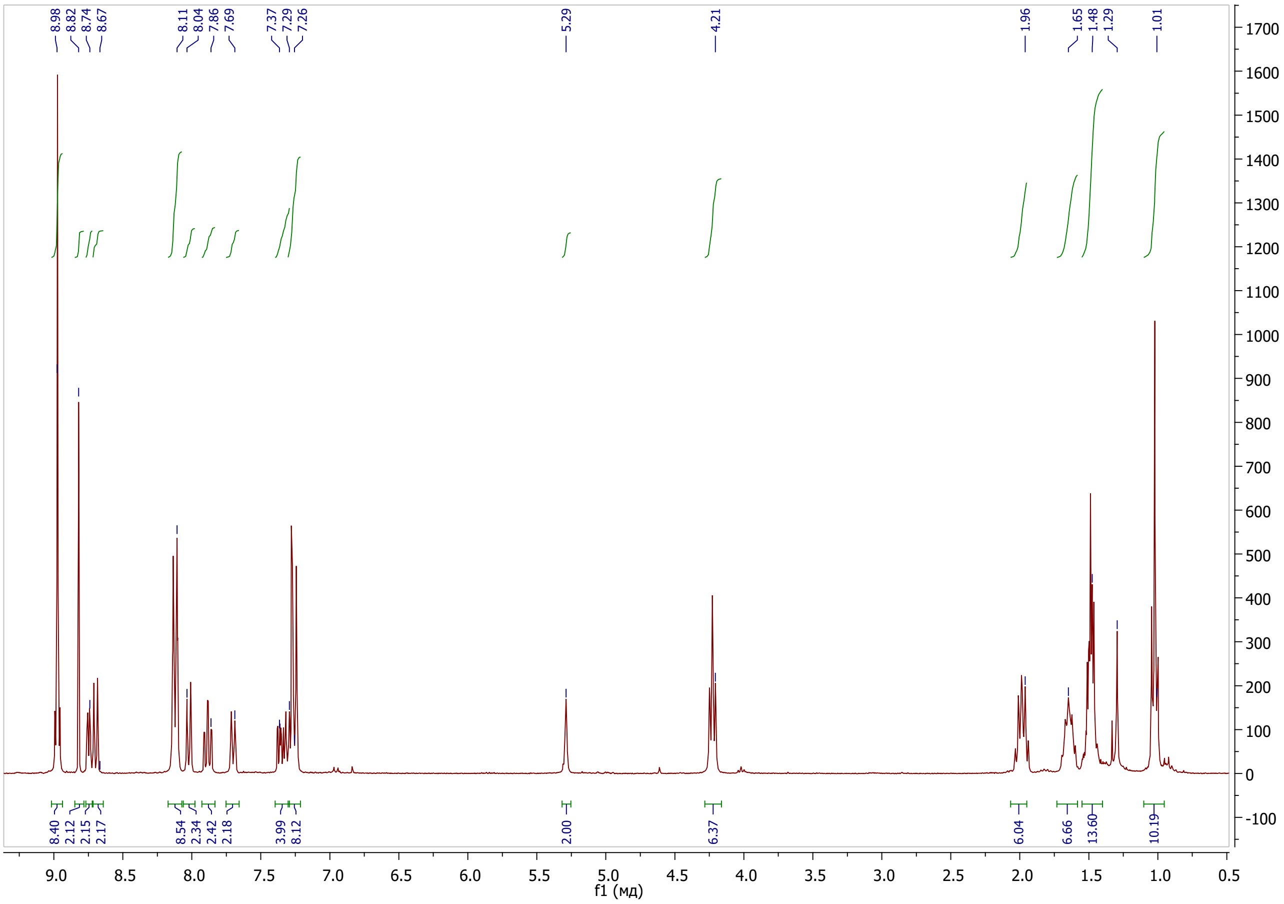

Figure S36. ^{1}H-NMR spectrum ($CDCl_3$) of compound **12.**

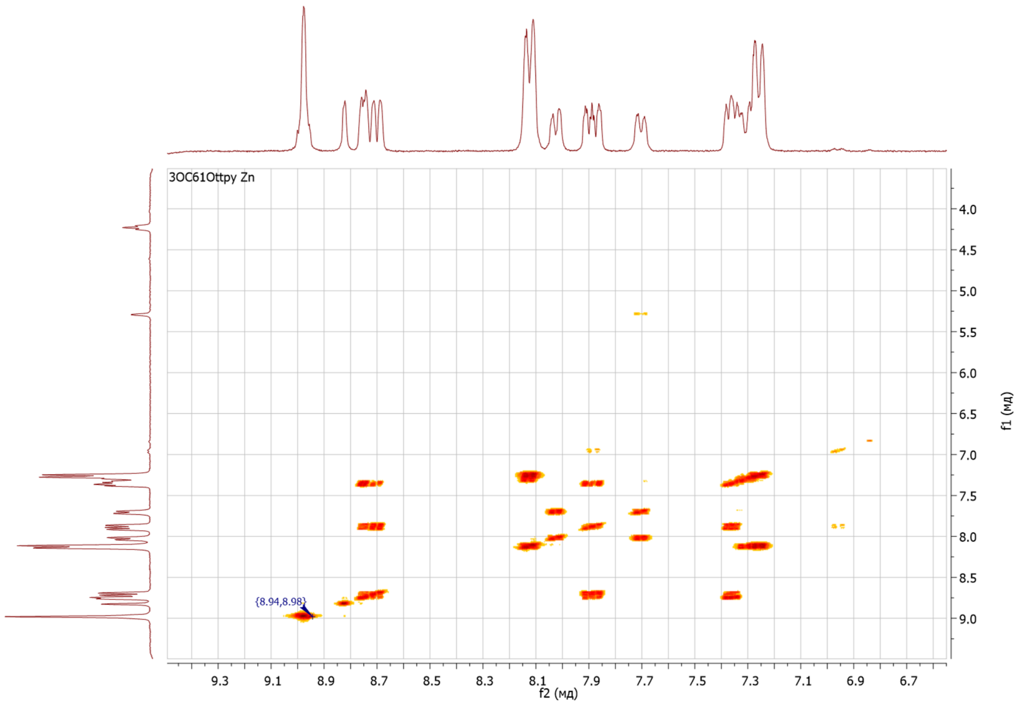

Figure S37. ^{1}H-^{1}H COSY NMR spectrum ($CDCl_3$) of compound **12**.

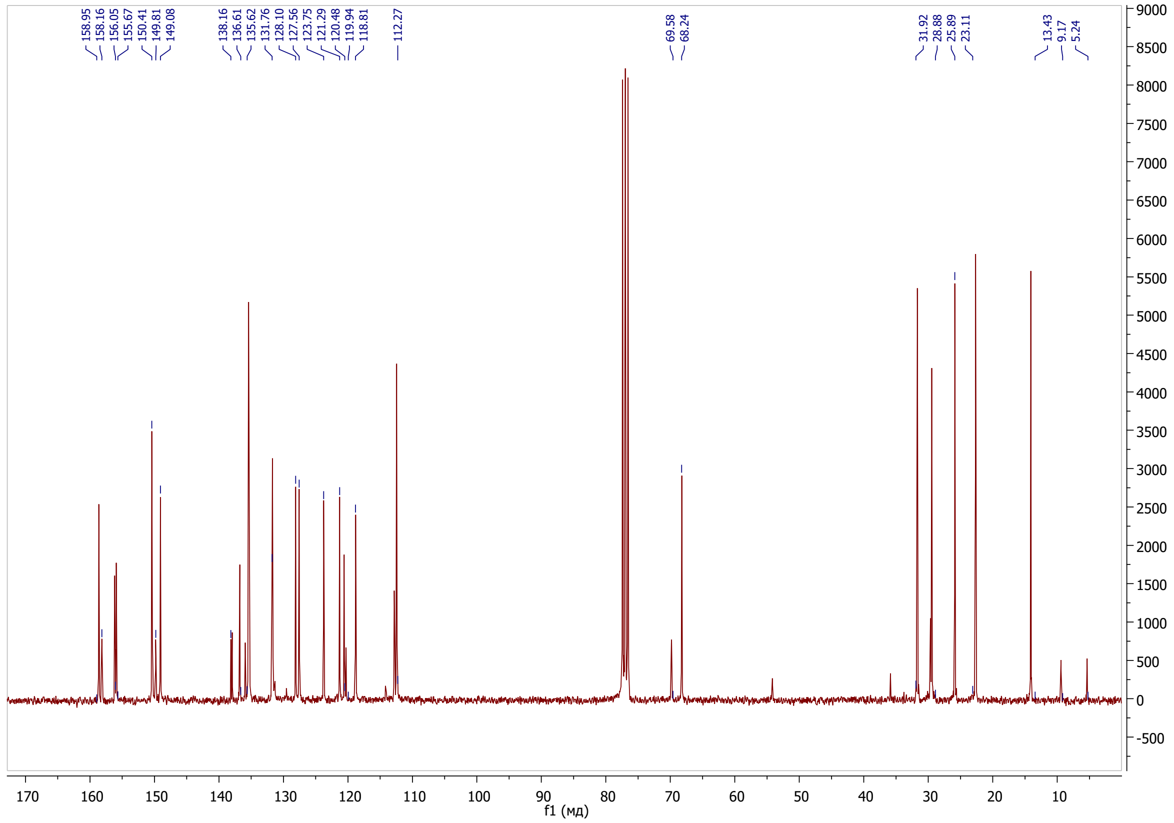

Figure S38. ^{13}C-NMR spectrum ($CDCl_3$) of compound **12.**

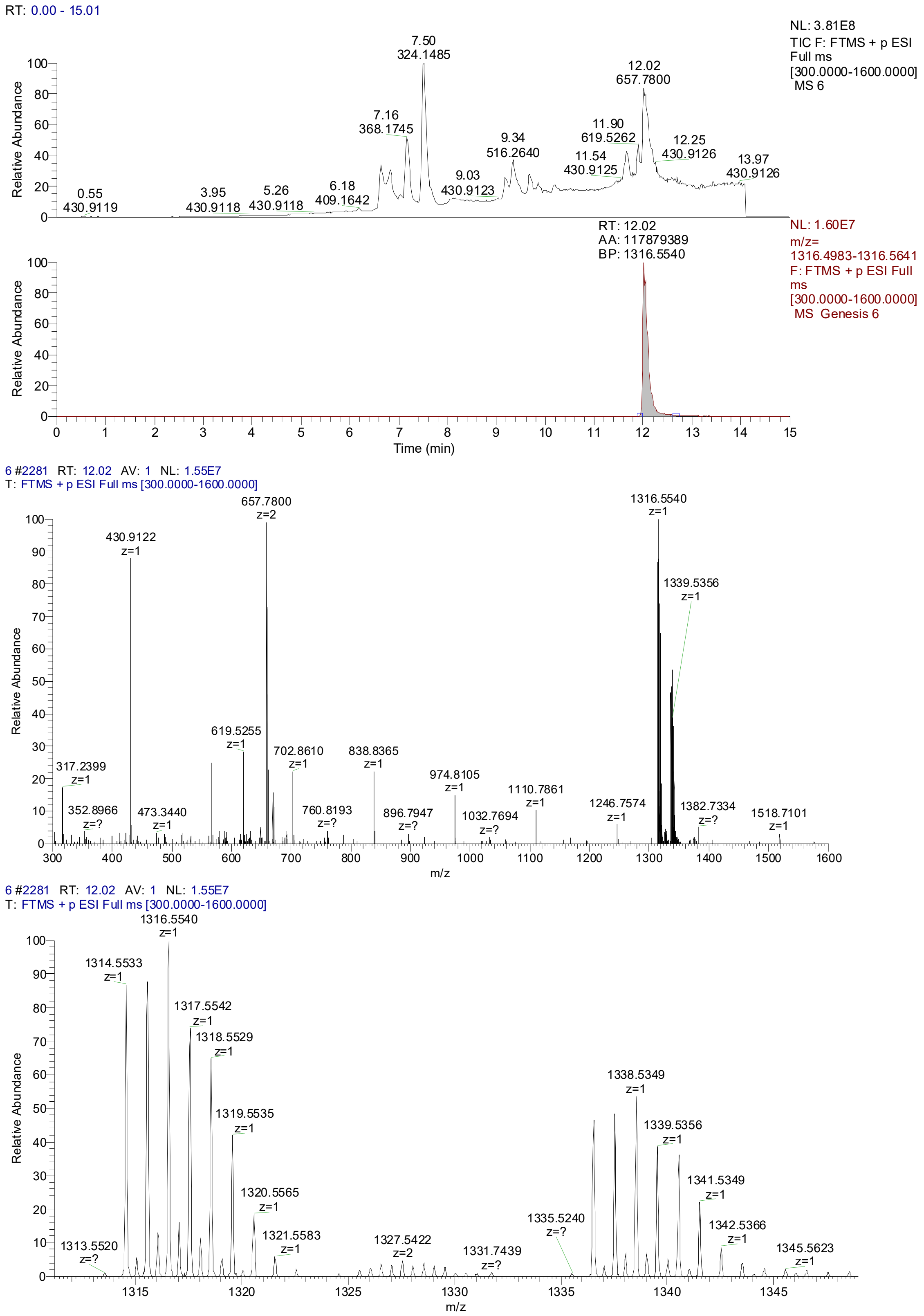

Figure S39. HRMS ESI of compound **12**

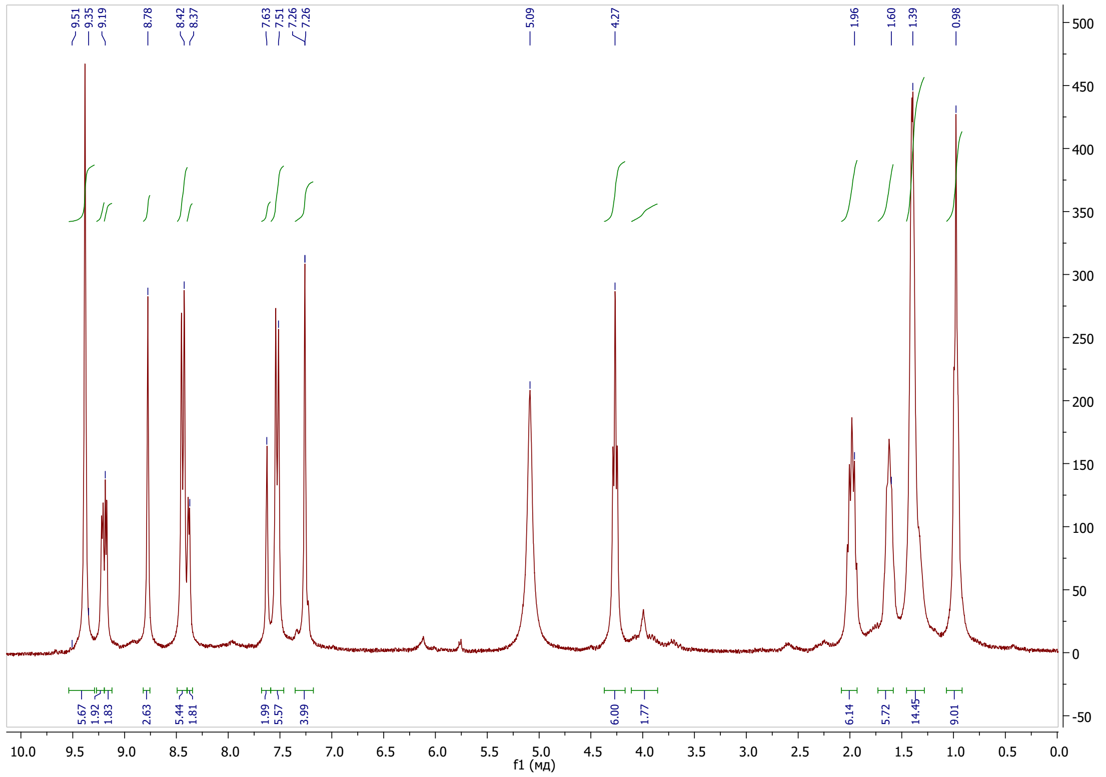

Figure S40. ^{1}H-NMR spectrum ($CDCl_3$) of compound **13**

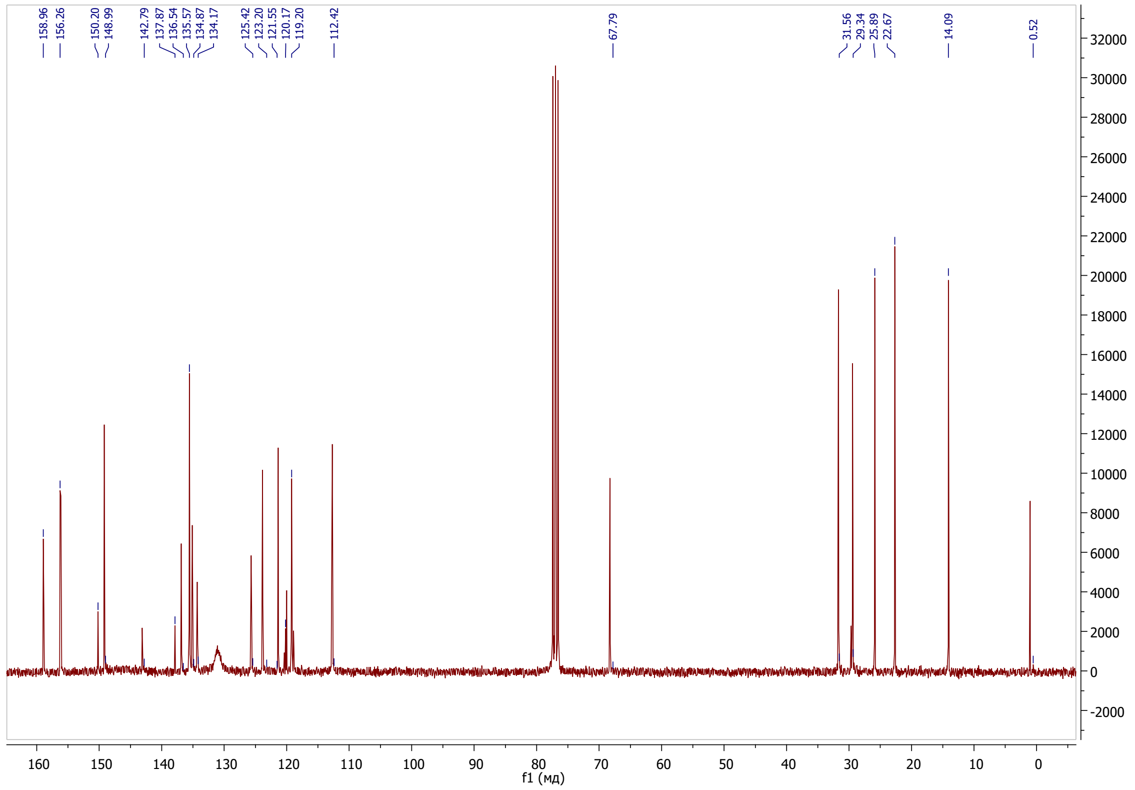

Figure S41. ^{13}C-NMR spectrum ($CDCl_3$) of compound **13**

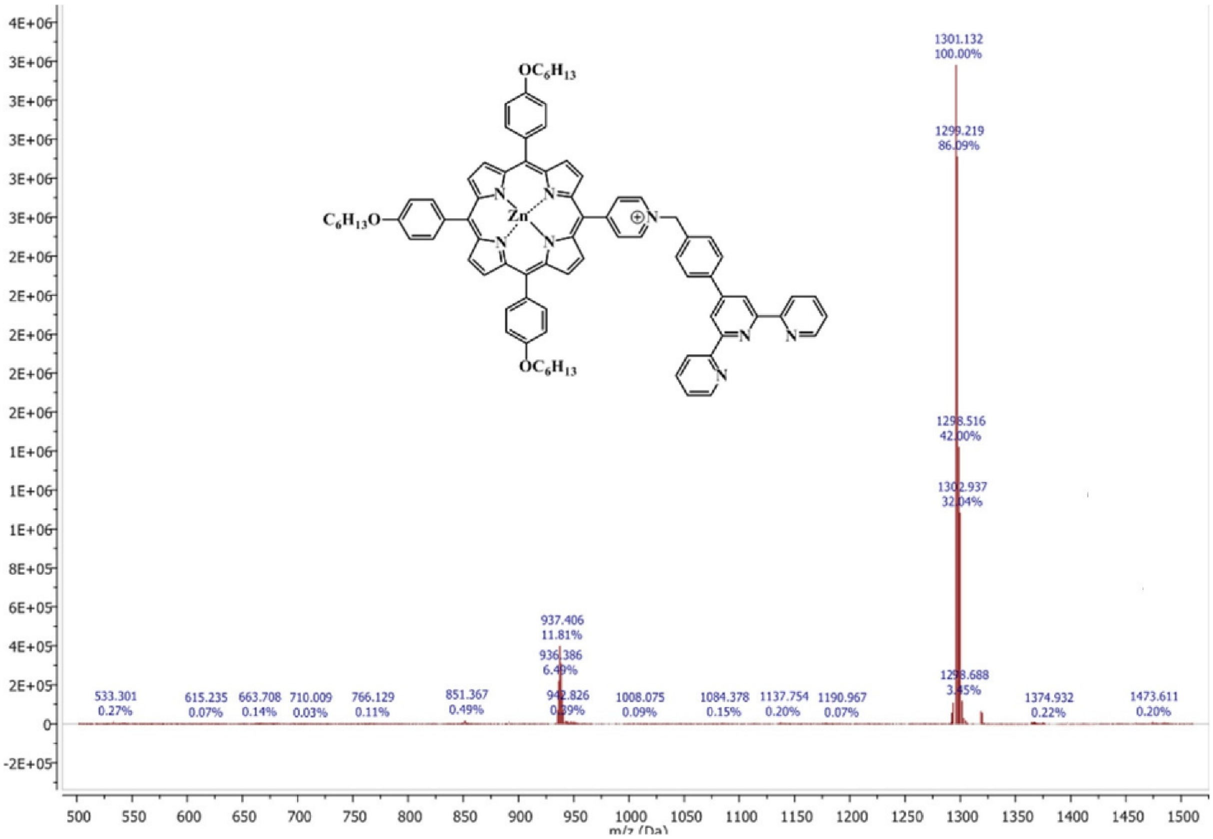

Figure S42. Experimental (upper) and predicted (lower) HRMS spectra of compound **11** $[M+Na]^{+}$.

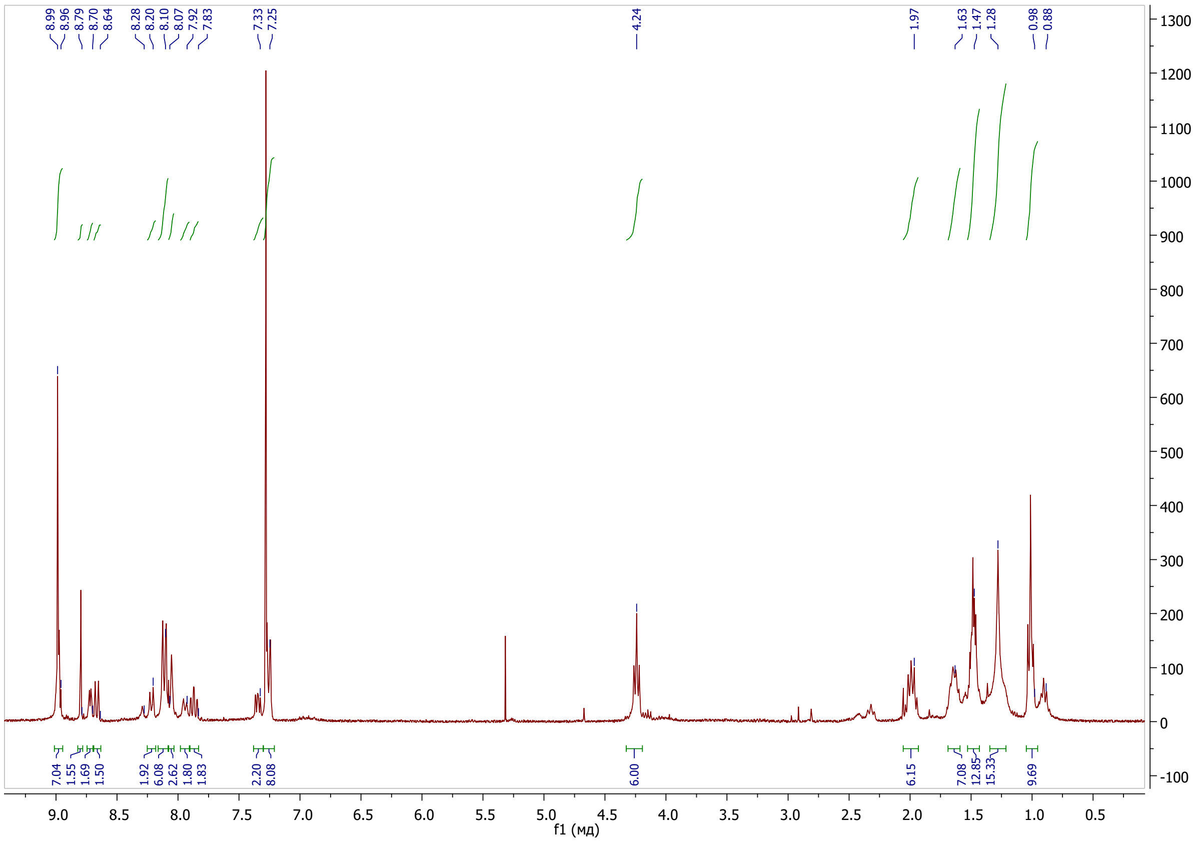

Figure S43. ^{1}H-NMR spectrum ($CDCl_3$) of compound **14**

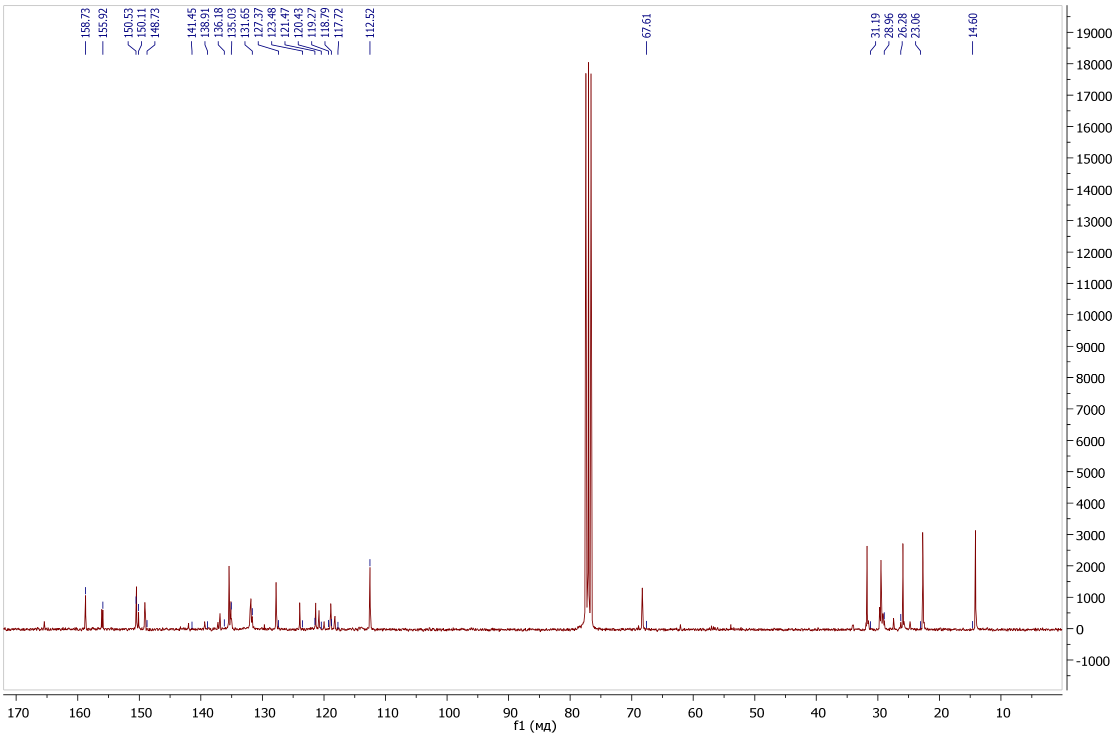

Figure S44. ^{13}C-NMR spectrum (CDCl3) of compound **14**

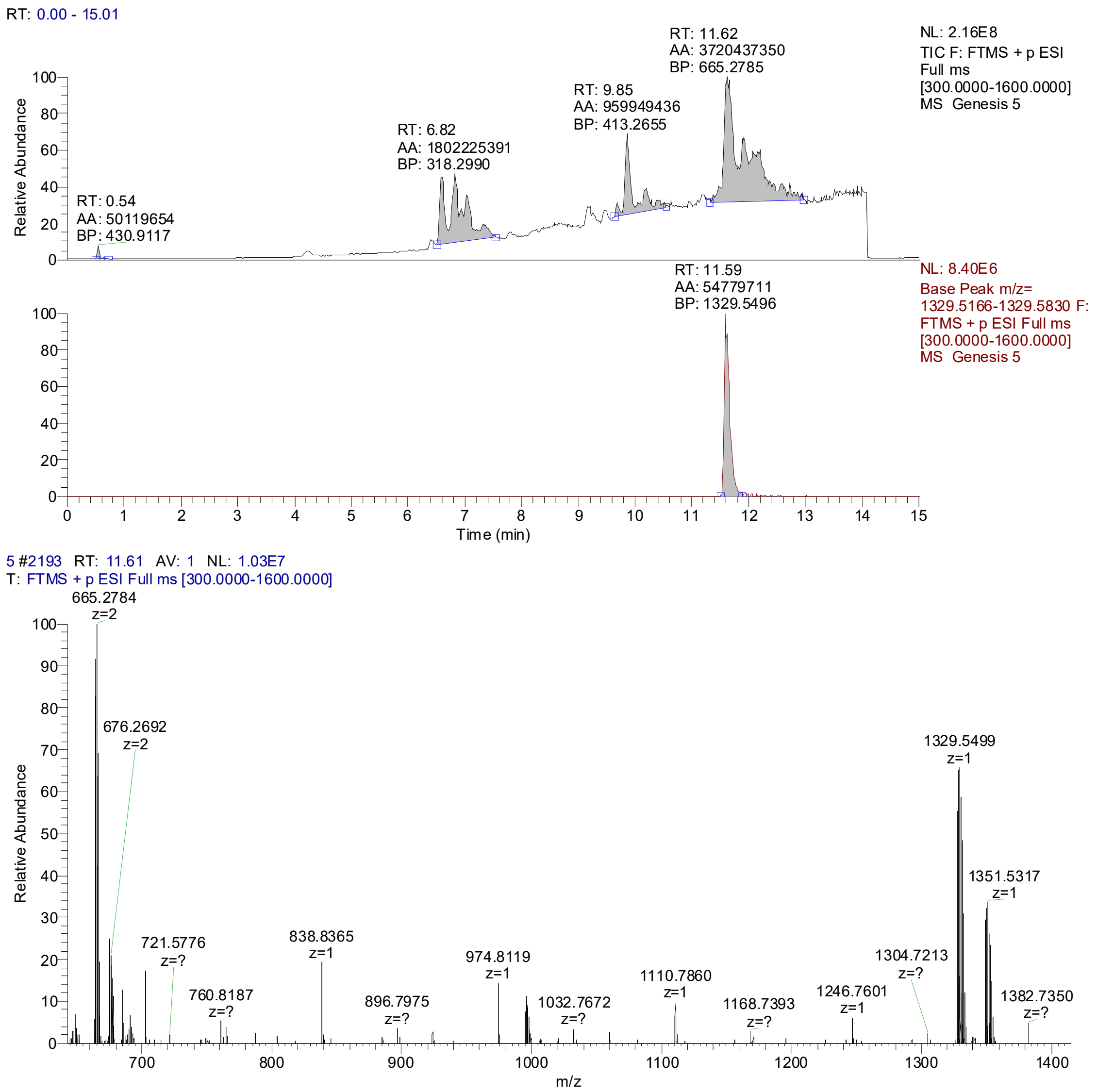
RT: 0.00 - 15.01
RT: 11.62
AA: 3720437350
BP: 665.2785
NL: 2.16E8
TIC F: FTMS + p ESI
Full ms
[300.0000-1600.0000]
MS Genesis 5
RT: 9.85
AA: 959949436
BP: 413.2655
RT: 6.82
AA: 1802225391
BP: 318.2990
RT: 0.54
AA: 50119654
BP: 430.9117
Relative Abundance
RT: 11.59
AA: 54779711
BP: 1329.5496
NL: 8.40E6
Base Peak m/z=
1329.5166-1329.5830 F:
FTMS + p ESI Full ms
[300.0000-1600.0000]
MS Genesis 5
Time (min)
5 #2193 RT: 11.61 AV: 1 NL: 1.03E7
T: FTMS + p ESI Full ms [300.0000-1600.0000]
665.2784
z=2
676.2692
z=2
721.5776
z=?
760.8187
z=?
838.8365
z=1
896.7975
z=?
974.8119
z=1
1032.7672
z=?
1110.7860
z=1
1168.7393
z=?
1246.7601
z=1
1304.7213
z=?
1329.5499
z=1
1351.5317
z=1
1382.7350
z=?
Relative Abundance
m/z

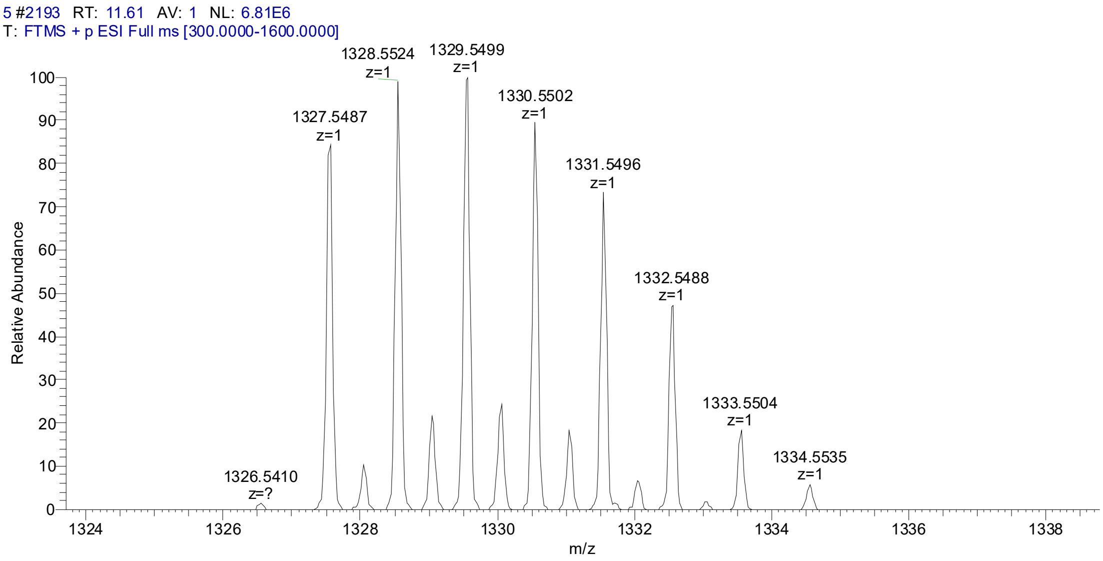

Figure S45. HRMS-ESI of compound **14**

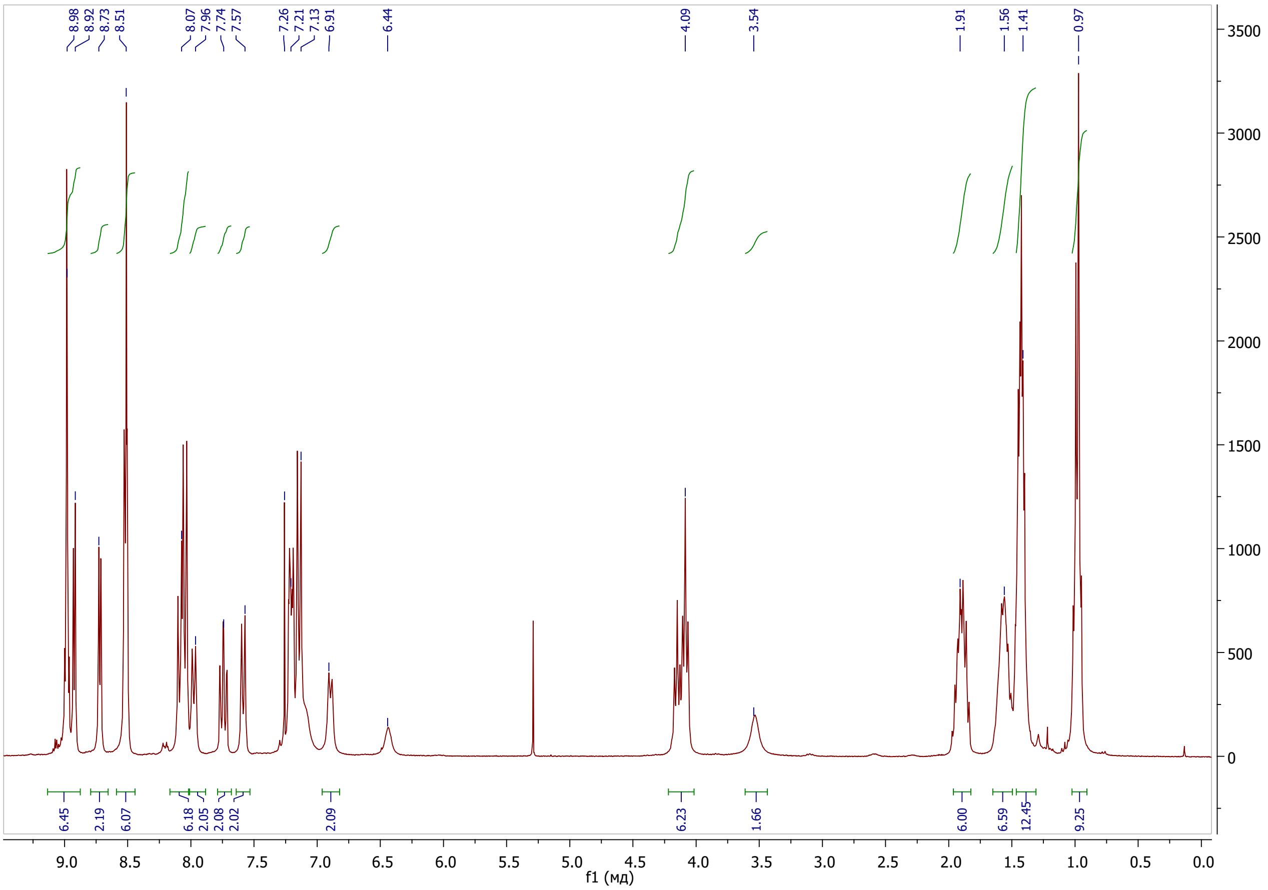

Figure S46. ^{1}H-NMR spectrum ($CDCl_3$) of compound **15**

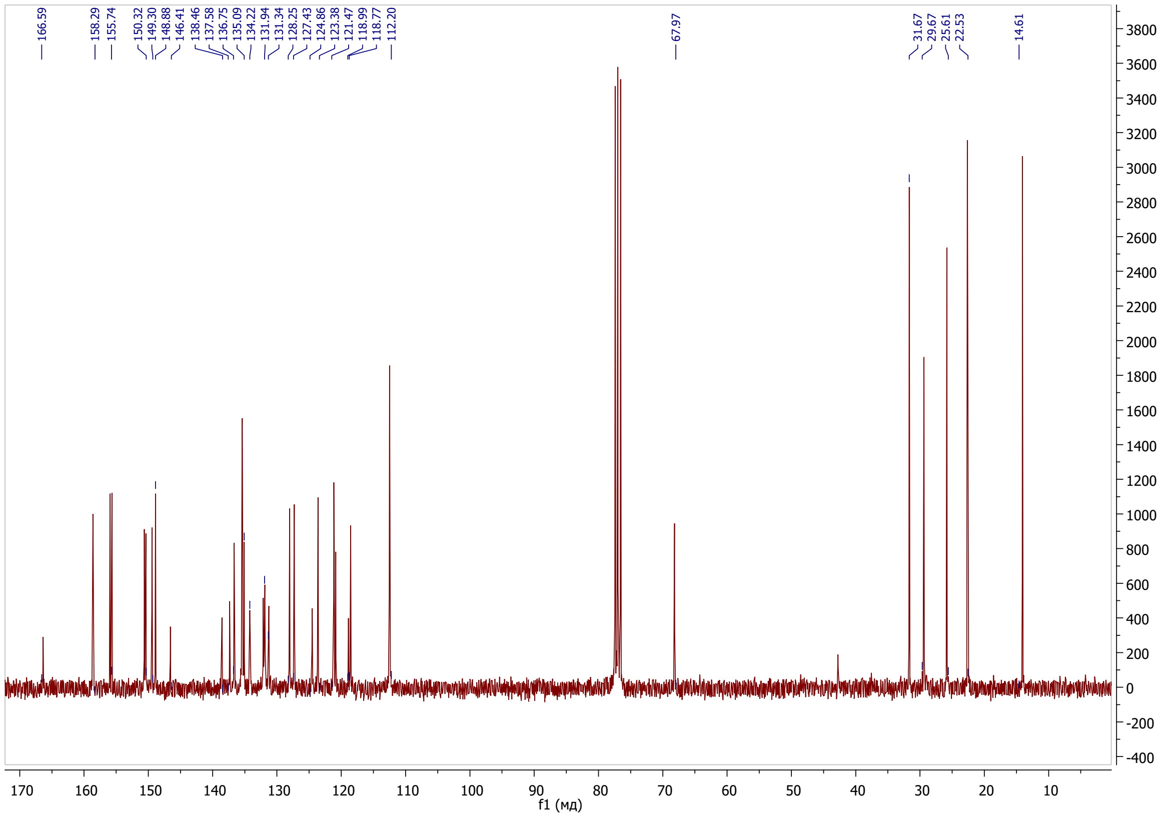

Figure S47. ^{13}C-NMR spectrum ($CDCl_3$) of compound **15**

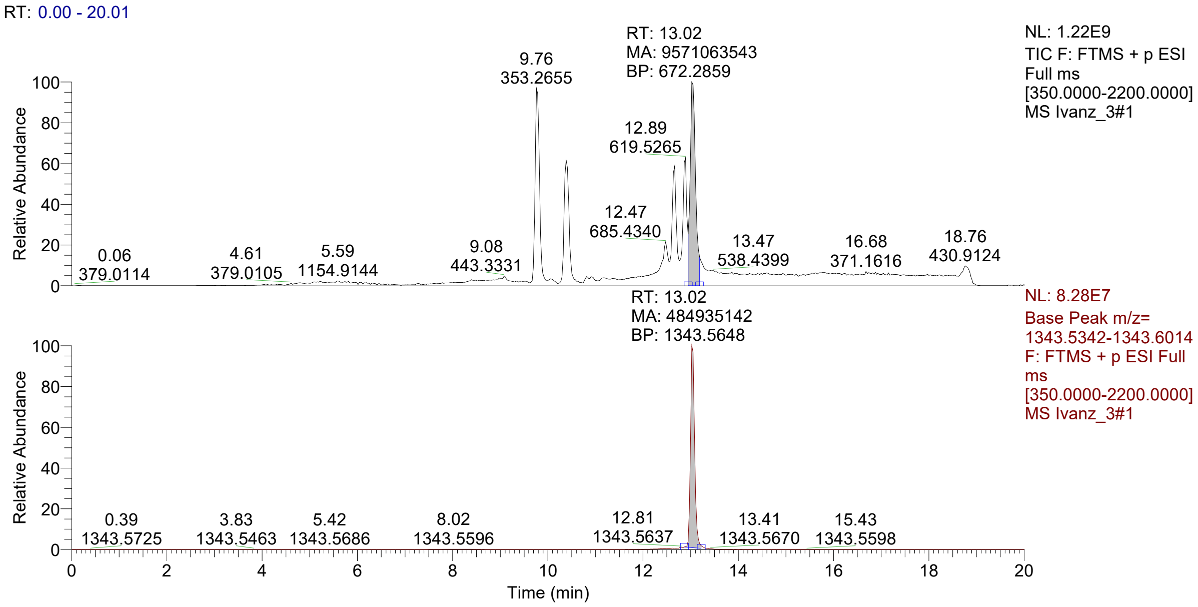

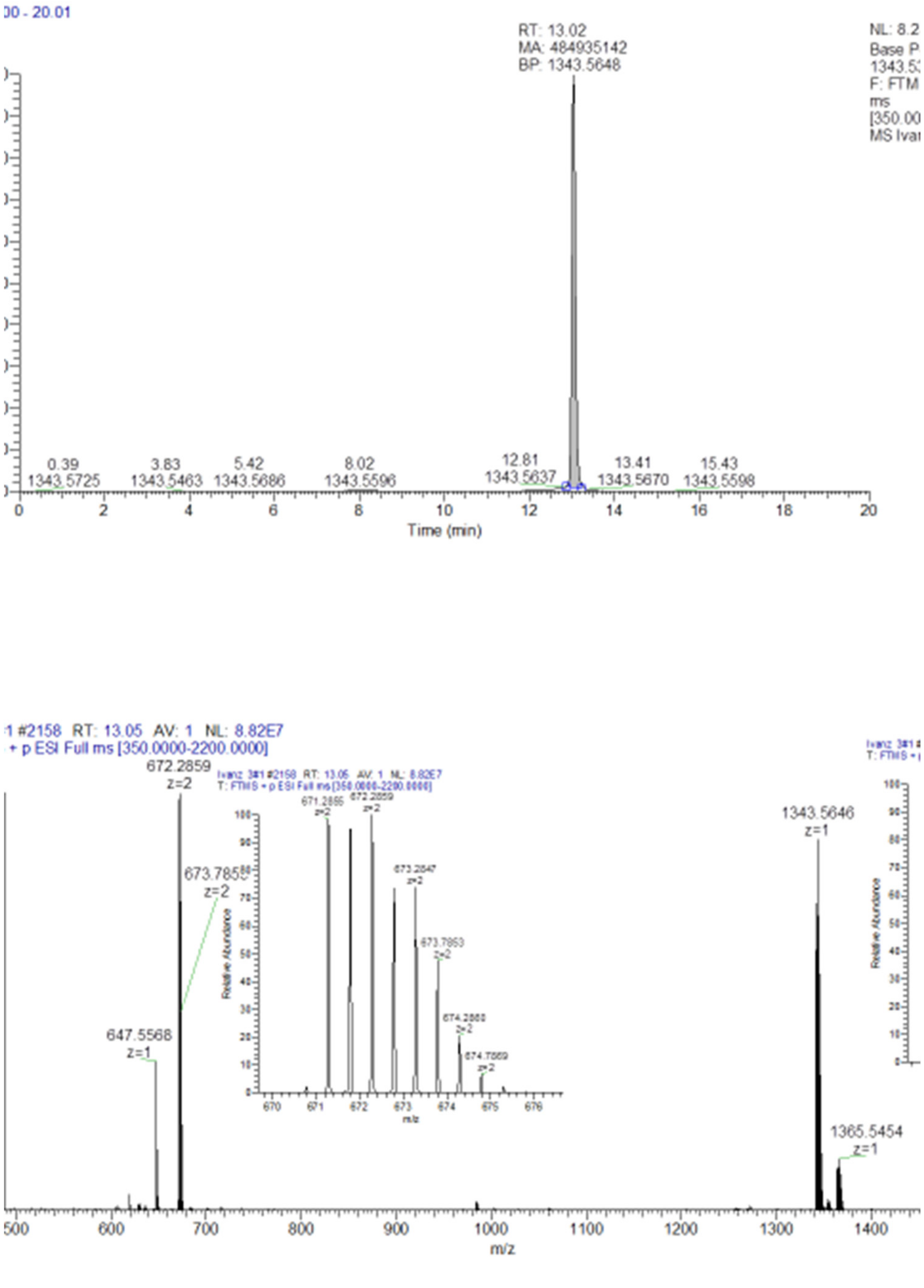

Figure S48. HRMS-ESI of compound **15.**

Mass-spectra of complexes with paramagnetic metals S49-S56 are shown below

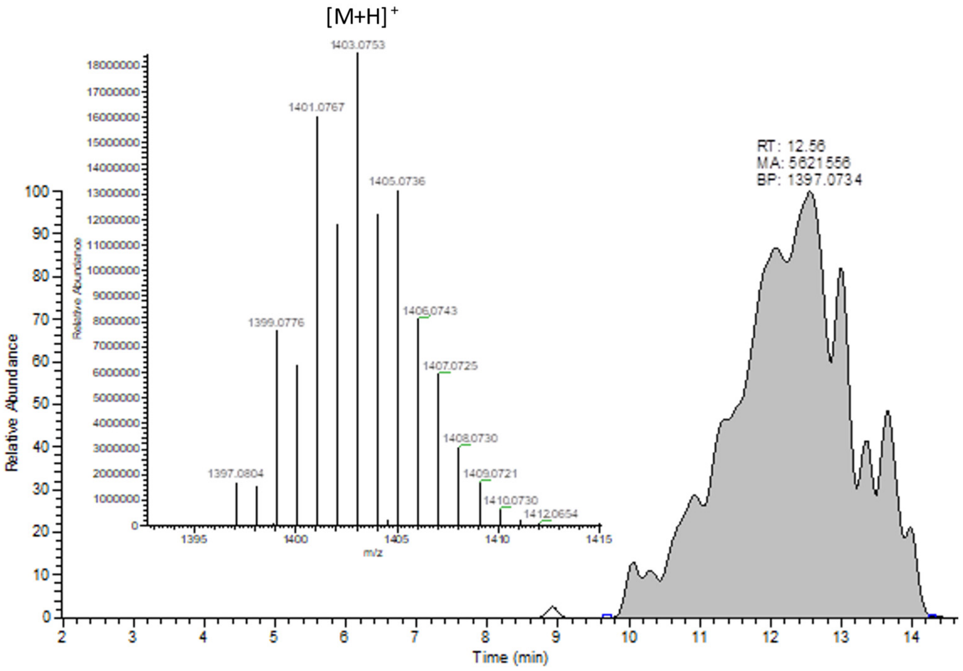

Figure S49. HRMS mass spectrum of compound **12a[1]**.

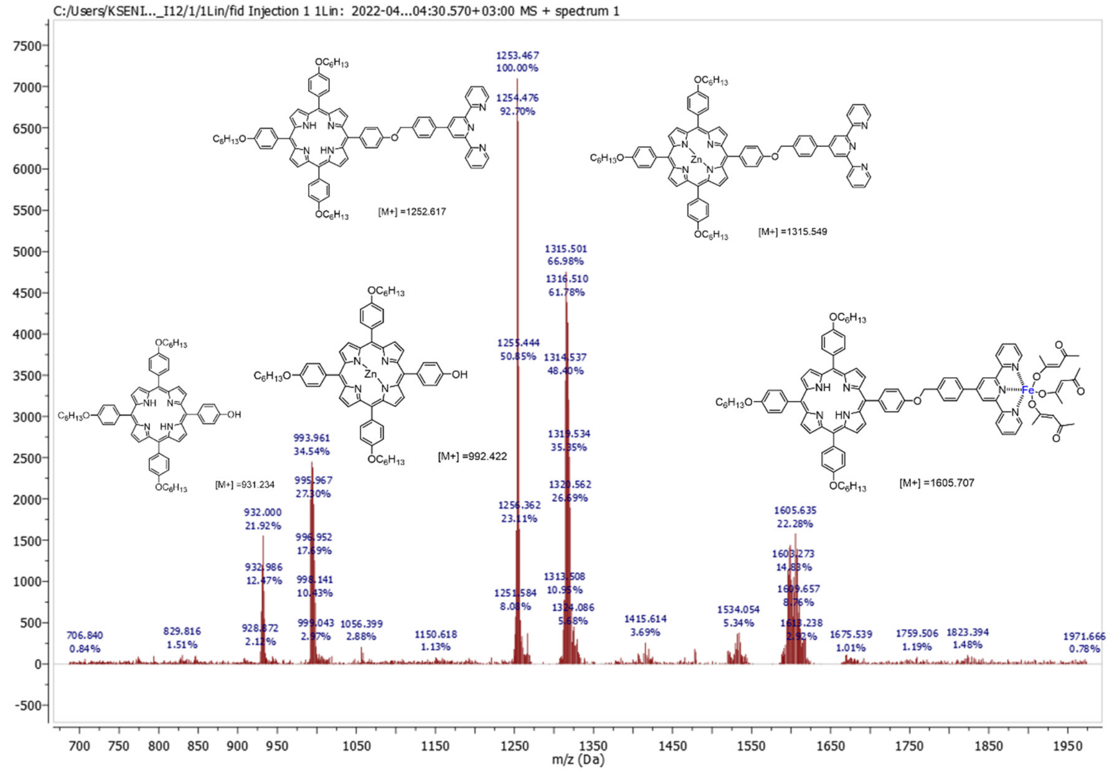

Figure S50. MALDI – TOF Mass-spectrum of compound **12a[2]**.

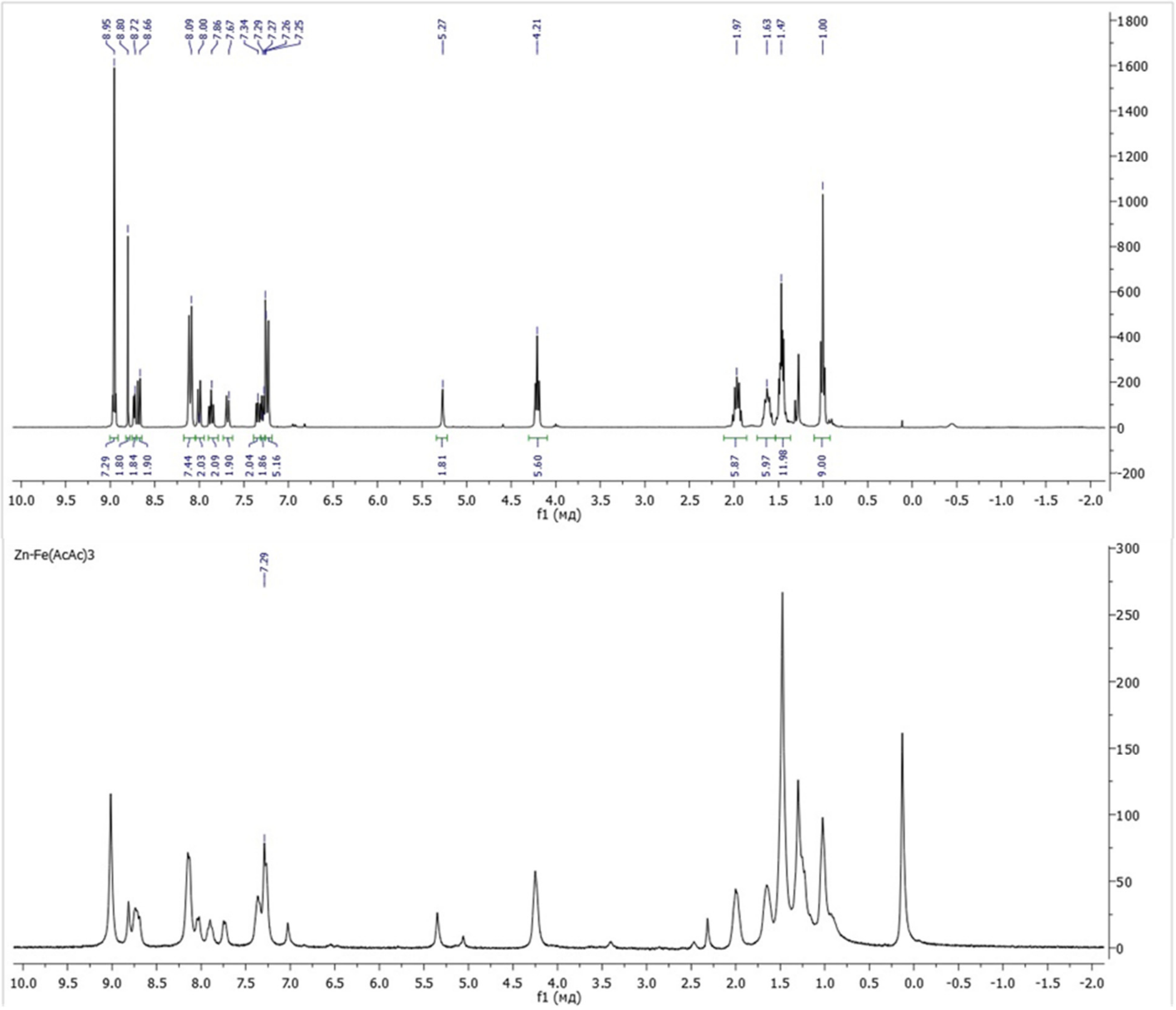

Figure S51. ^{1}H NMR spectra of conjugate 12 (upper) and its complex with $Fe(AcAc)_3$ (lower)

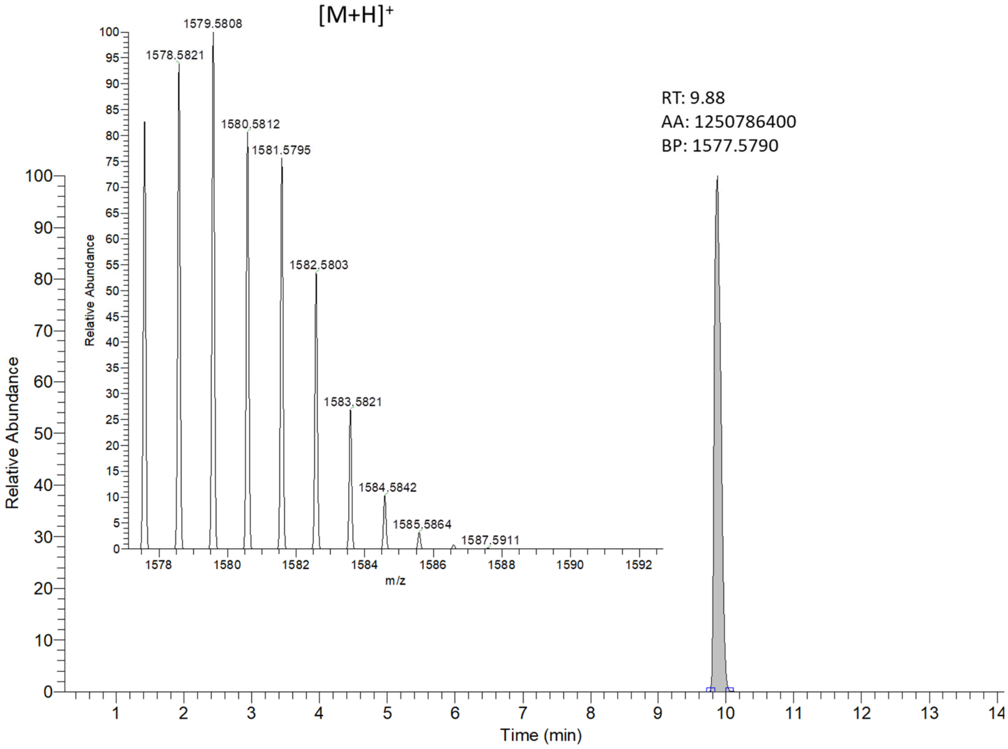

Figure S52. HRMS-ESI of compound **12b[1].**

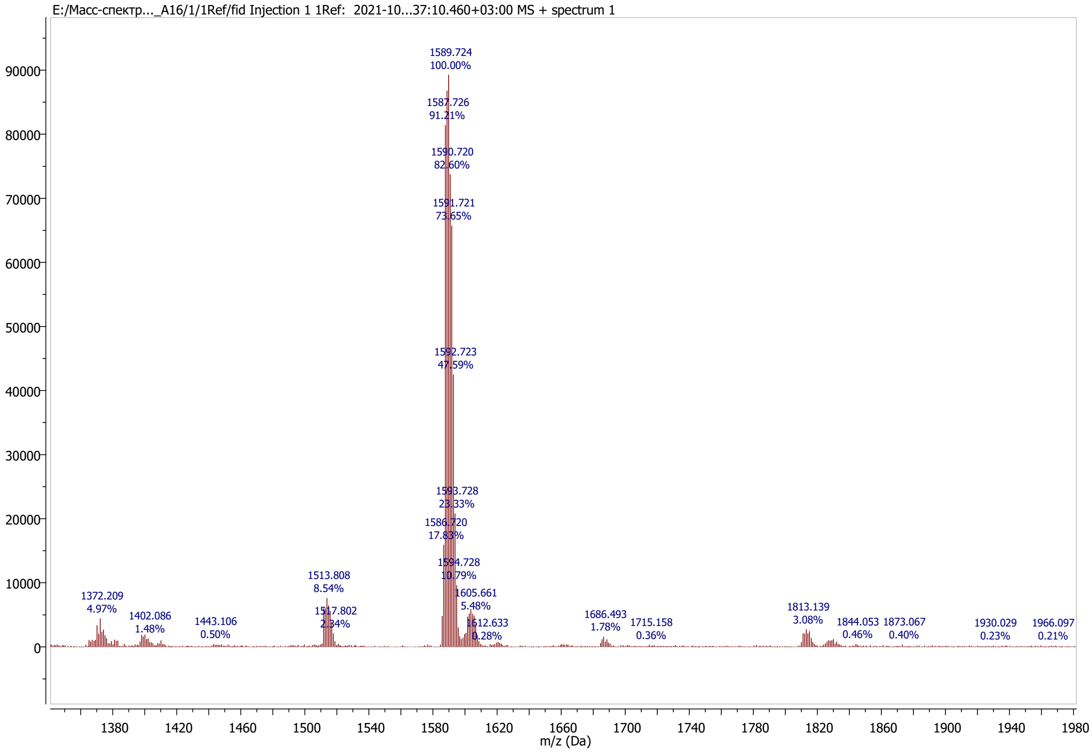

Figure S53. MALDI – TOF Mass-spectrum of compound **13b[1]**.

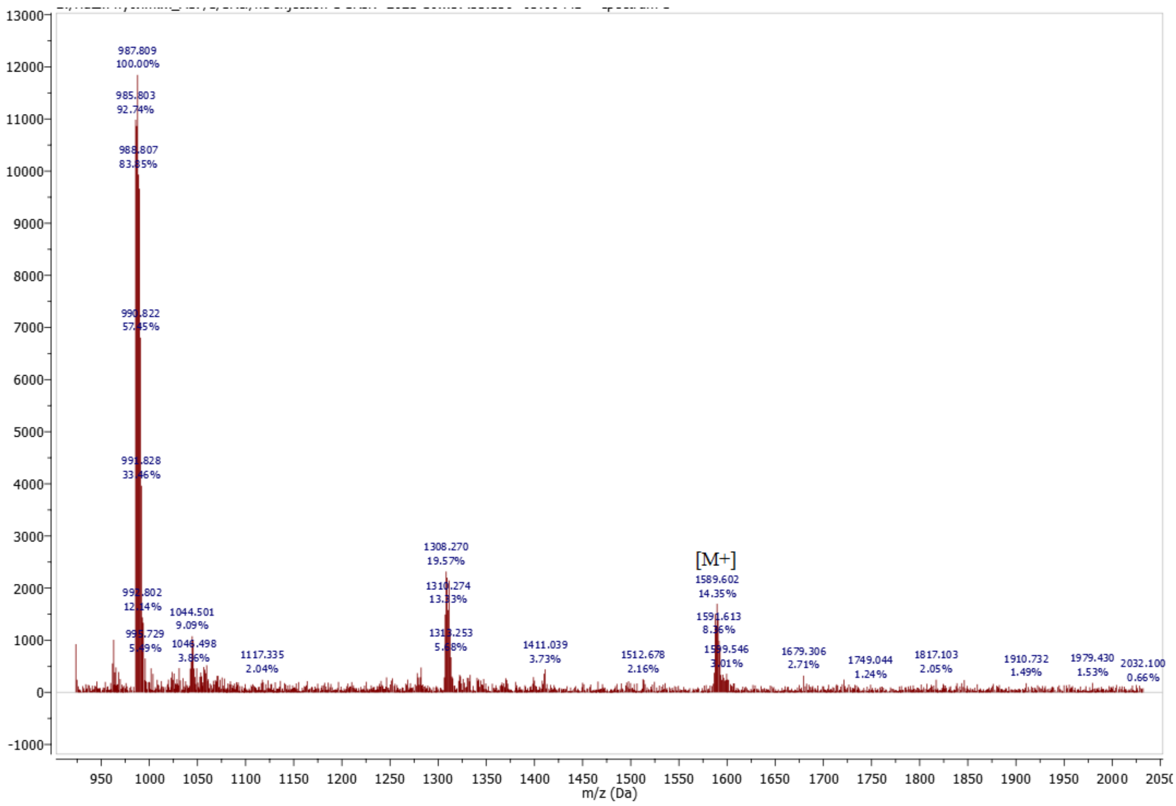

Figure S54. MALDI – TOF Mass-spectrum of compound **14b**[1].

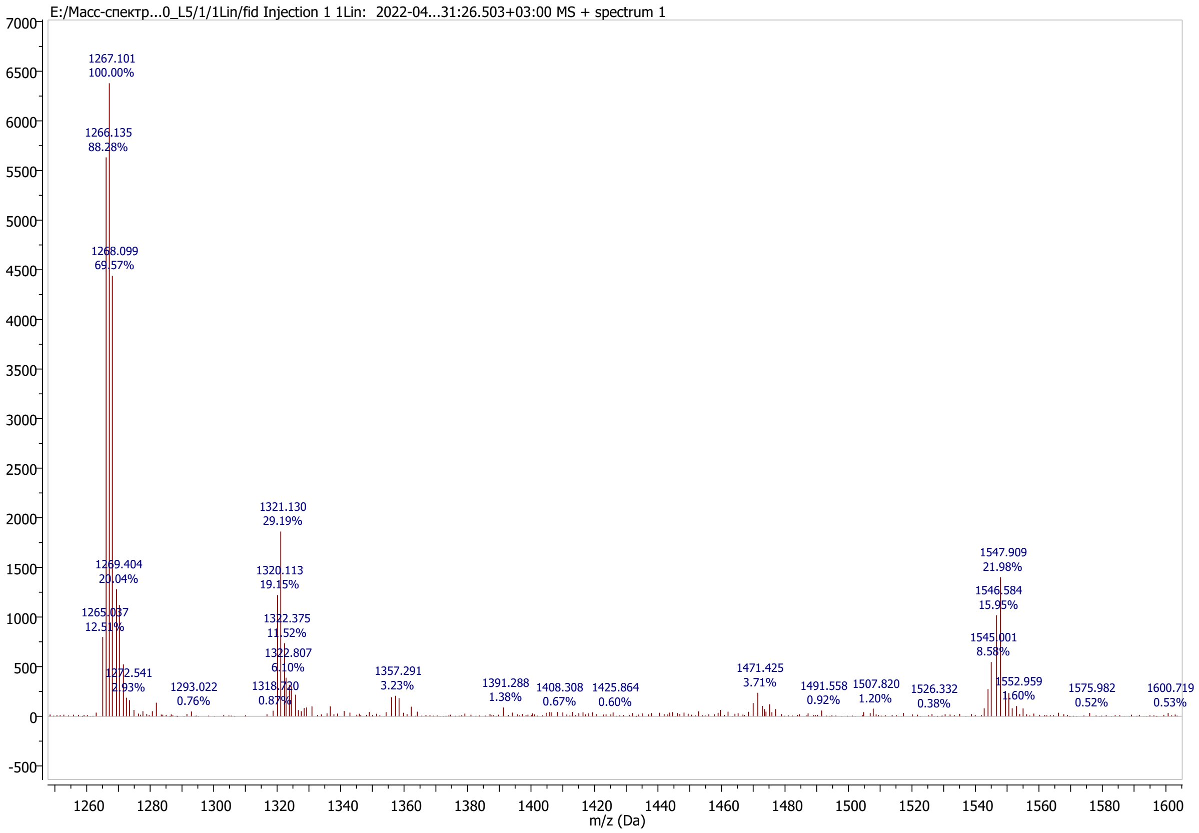

Figure S55. MALDI – TOF Mass-spectrum of compound **15b**[1].

Particle size distribution

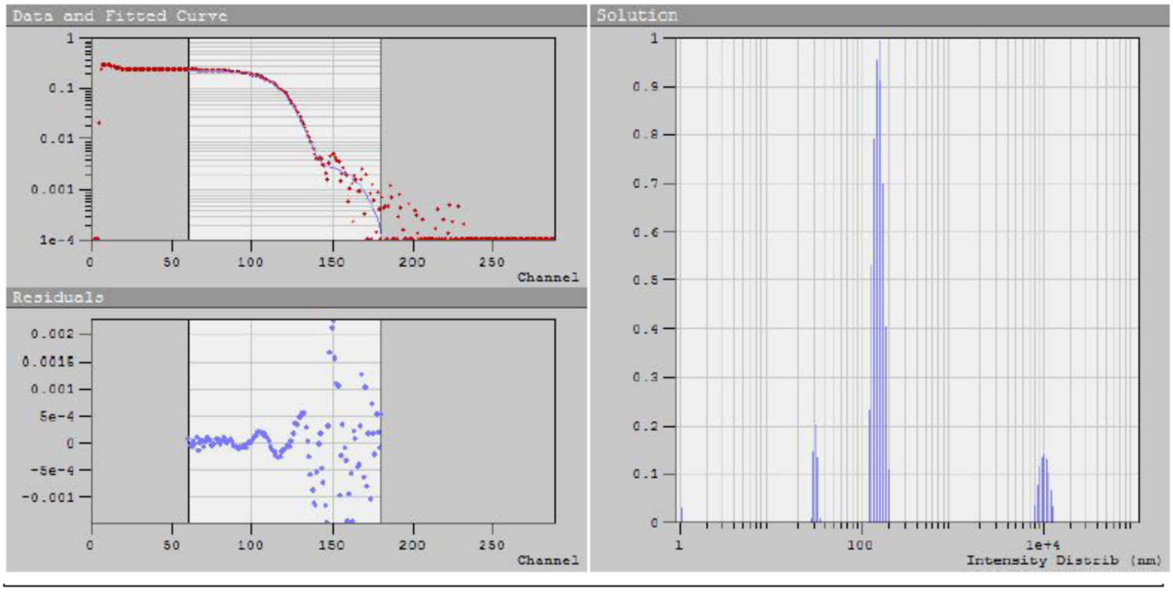

Figure S56. Particle size distribition of conjugate **12**.

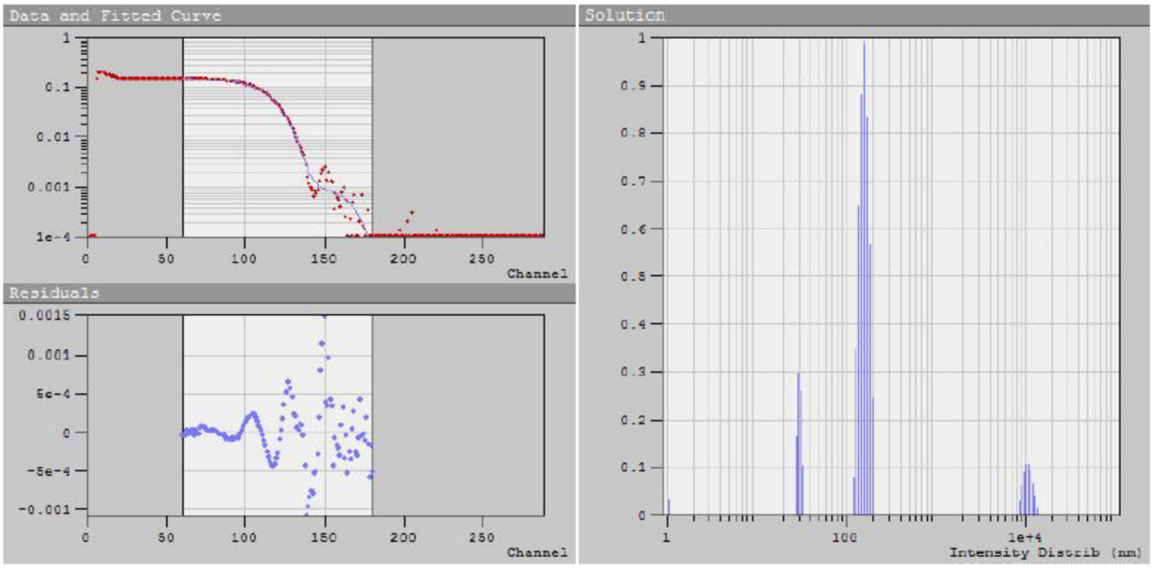

Figure S57. Particle size distribition of conjugate **14**.

Cell viability study (MTT - test) on NKE cells.

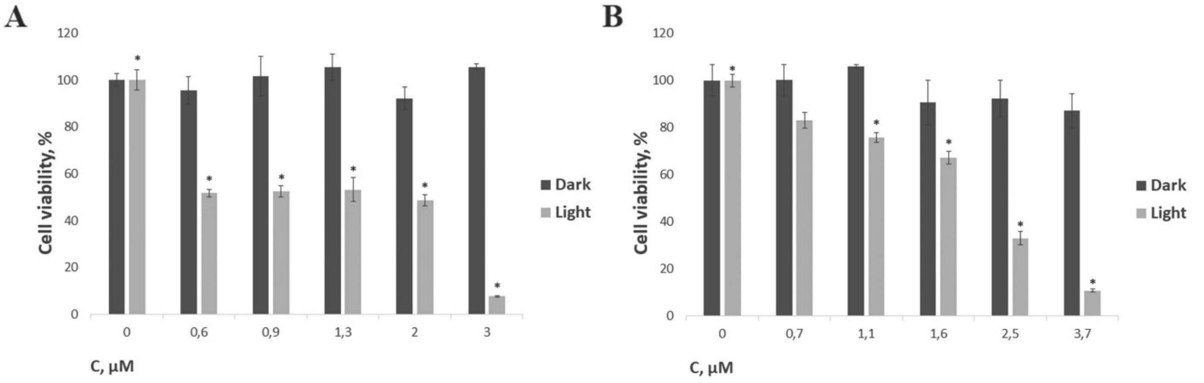

Figure S58. Effect of compounds on the viability of NKE cell line. Cells were irradiated for 90 minutes using the Medical Therapy Philips TL 20W/52 lamp (irradiation dose of 8.073 J/cm^2). Incubation of cells with the compound without irradiation in the dark for 90 minutes. * statistically significant differences in cell survival relative to values with zero concentration of the compound (p<0.01) were noted. A) conjugate **12**; B) conjugate **14**.

Influence on the time/dose irradiation

As part of the optimization of experimental conditions, the effect of irradiation on the viability of the Hep2 cell line was evaluated. A medical lamp Therapy Philips TL 20W/52 lamp (wavelength 400-500 nm), power 2.3 mW was used.

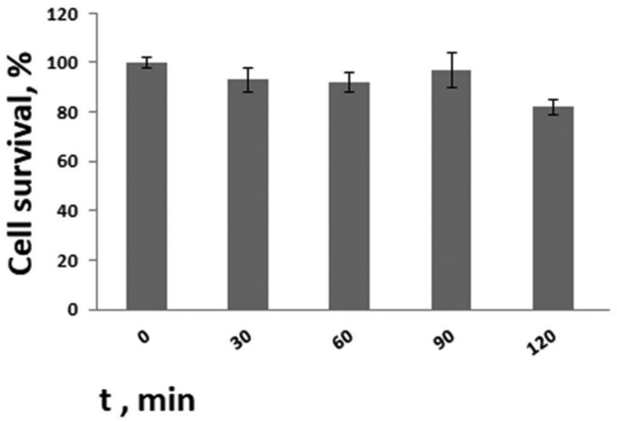

Figure S59. Effect of irradiation time/dose of the Medical Therapy Philips TLW/52 lamp on the viability of the Hep2 cell line. 0 min - irradiation dose of 0 J/cm^2, 30 min - irradiation dose of 2.691 J/cm^2, 60 min - irradiation dose of 5.382 J/cm^2, 90 min - irradiation dose of 8.073 J/cm^2, 120 min - irradiation dose of 10.764 J/cm^2.

Gap junction-mediated intercellular communication test of compound 14.

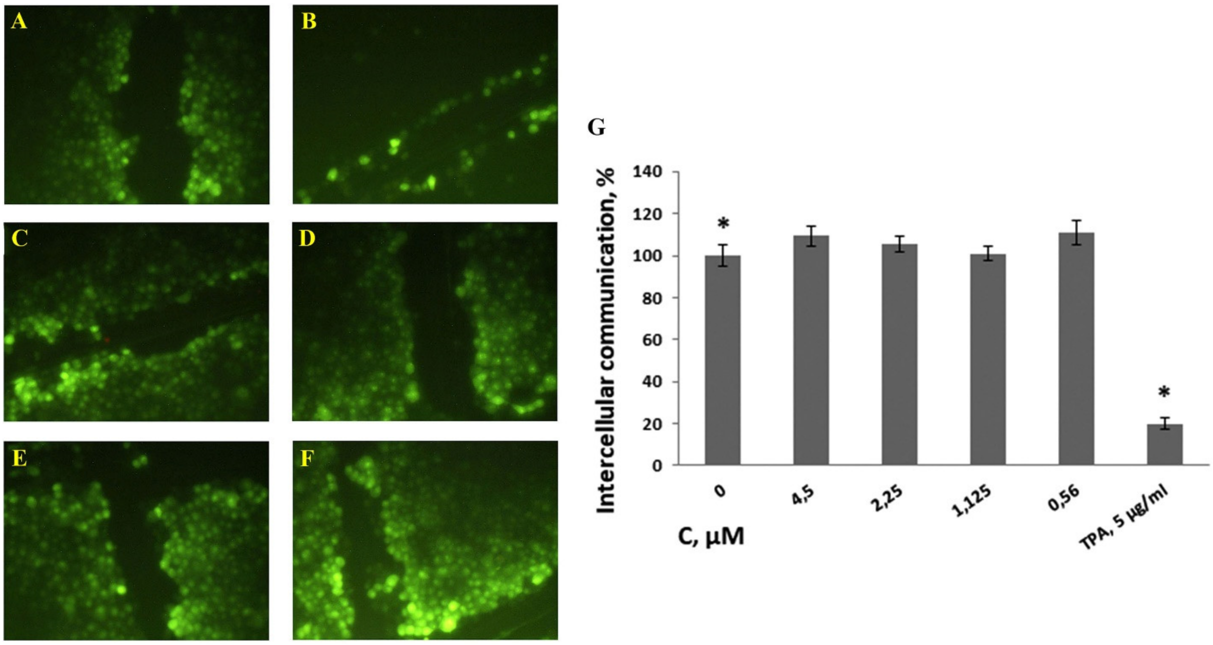

Figure S60. Micrographs of the cell monolayer in the scratch area of control and experimental samples. A. Negative control (dH_2O). B. Positive control (TPA, 5 µg/ml). Compound **14**: 4.5 µM (C); 2.25 µM (D); 1.125 µM (E) and 0.56 µM (F). LY propagation length in 4 min.
